# Hepatitis B and C prevalence and incidence in key population groups with multiple risk factors in the EU/EEA: a systematic review

**DOI:** 10.2807/1560-7917.ES.2019.24.30.1800614

**Published:** 2019-07-25

**Authors:** Lauren MK Mason, Erika Duffell, Irene K Veldhuijzen, Uarda Petriti, Eveline M Bunge, Lara Tavoschi

**Affiliations:** 1 Pallas Health Research and Consultancy B.V., Rotterdam, Netherlands; 2 European Centre for Disease Prevention and Control, Stockholm, Sweden; 3 National Institute for Public Health and the Environment, Bilthoven, Netherlands; 4 Current affiliation: University of Pisa, Pisa, Italy

**Keywords:** hepatitis B, hepatitis C, epidemiology, PLHIV, prisoners

## Abstract

**Background:**

People living with HIV (PLHIV) and people in prison are population groups with a potentially high risk and/or prevalence of hepatitis B virus (HBV) and hepatitis C virus (HCV) infection.

**Aim:**

We conducted a systematic review in order to find prevalence and incidence estimates in these populations in the European Union/European Economic Area (EU/EEA).

**Methods:**

Original research articles published between January 2005 and February 2017 were retrieved from PubMed and Embase in February 2017.

**Results:**

Fifty-two articles were included, providing 97 estimates of HBV/HCV infection prevalence or incidence. Estimates of HBV infection prevalence ranged between 2.9% and43.4% in PLHIV and 0.0% and 25.2% in people in prison. Estimates of HCV infection prevalence ranged from 2.9% to 43.4% in PLHIV and 0.0% to 25.2% in people in prison. Incidence estimates ranged between 0.0 and 2.5 cases per 100 person-years for HBV infection in PLHIV. No such data was available for people in prison. HCV infection incidence ranged between 0.3 and 0.9 cases per 100 person-years in PLHIV and between 1 and 1.2 cases per 100 person-years in people in prison. Prevalence estimates were generally higher than in the general population, especially for HCV infection and among groups with multiple risk factors.

**Conclusions:**

PLHIV, people in prison and groups with multiple risk factors, have a high prevalence of HBV and HCV and may be at ongoing risk of infection. These groups should be among the populations prioritised and targeted for active case finding and prevention programmes in the EU/EEA.

## Introduction

Worldwide, an estimated 248 million [1] and 71.1 million [2] people are chronically infected with the hepatitis B virus (HBV) and hepatitis C virus (HCV), respectively. Across the European Union/European Economic Area (EU/EEA) this is estimated at 4.7 million for HBV infection and 3.9 million for HCV infection [3]. HBV and HCV can cause acute and chronic hepatitis, and can potentially lead to the development of cirrhosis, liver cancer or even death [4,5]. Since onset of disease and initial development of liver damage are often asymptomatic [6-8], HBV and HCV infection may go undetected for many years [9]. Recent estimates indicate that the majority of the chronically infected population remains undiagnosed [10-12].

Transmission of HBV and HCV can occur via blood-blood contact, including during intravenous drug use or nosocomial transmission, or vertically, mother-to-child. It can also be transmitted sexually. People living with HIV (PLHIV) and people in prison are key population groups potentially at high risk for being infected with HBV and HCV [13]. As HIV shares transmission routes with HBV and HCV, PLHIV may have been exposed or have ongoing exposure to HBV and HCV. Moreover, the transmission efficiency of HCV is increased in the presence of HIV [14]. Spontaneous clearance of HBV or HCV is less likely in PLHIV, with higher viral loads and more rapid and severe disease progression [15,16]. Both a history of intravenous drug use and continuing intravenous drug use are common among people in prison [17,18]. Tattooing in prison settings may pose an additional risk of HBV/HCV transmission [19]. Furthermore, unsafe sexual behaviour in this setting with a common lack of access to condoms may be a risk factor for infection. Subpopulations with multiple, overlapping risk factors, such as people in prison living with HIV, people who inject drugs (PWID) or men who have sex with men (MSM) in either population may have an even higher risk or prevalence of disease.

Treatment of chronic hepatitis B is highly effective and leads to viral suppression in 90% of cases [20]. Recently, new drug therapies have been introduced for HCV infection which achieve cure rates of over 95% [21]. The existence of effective treatment options for HBV and HCV infection, and effective vaccination against hepatitis B places emphasis on public health organisations to step up their responses to these diseases. In 2016, the World Health Organization (WHO) formulated an action plan to eliminate viral hepatitis as a public health threat in the European Region by 2030, aiming for 50% of people with chronic HBV/HCV infections to be diagnosed by 2020, and 90% by 2030 [22]. Scale-up of testing programmes is needed to decrease the undiagnosed fraction, particularly among the most affected populations, and to fast track elimination [22]. To establish the scale of the public health problem and inform the development of effective local strategies that target key risk groups, reliable prevalence and incidence estimates are needed of HBV and HCV infection in PLHIV and people in prison.

The aim of this systematic review was to retrieve recent EU/EEA-wide data on the prevalence and incidence of HBV and HCV infection in PLHIV and people in prison, as well as other key populations within these groups, such as PWID, to better understand the epidemiological situation. This review was conducted as part of a larger project to develop a European testing guidance for HBV and HCV, coordinated by the European Centre for Disease Prevention and Control (ECDC).

## Methods

### Search strategy and selection criteria

Original research articles were retrieved from PubMed and Embase databases in February 2017. Search strategies combined controlled (MeSH/Emtree terms) and natural vocabulary on terms for disease (HBV, HCV), terms for occurrence (incidence, prevalence), population subgroups (including PLHIV, people in prison) and geographic terms (EU/EEA) (see Supplement S1: Search Strings). The search strategy implemented encompassed a range of populations potentially at risk. However, for the purposes of this article, methods and results applying to PLHIV and people in prison are presented only. Results for other population groups are published elsewhere [23]. For articles on PLHIV, the search was limited to records published from 1 January 2005 to 14 February 2017. For articles on people in prison, prevalence data were retrieved from a previous systematic review on hepatitis B and C prevalence in the EU/EEA conducted by the ECDC. This included records published between 1 January 2005 and 31 December 2014 [24] and was updated in the current search with articles with data on prevalence published from 1 January 2015 to 14 February 2017, articles with data on incidence published from 1 Januray 2005 onwards, and articles with data on prevalence or incidence among people in prison with multiple risk factors from 1 January 2005 onwards. Articles in all EU/EEA languages were included.

Only articles reporting data from EU/EEA countries were included. Articles reporting prevalence of markers other than HBsAg, anti-HCV, HBV DNA or HCV RNA, unspecified markers, or self-reported infections were excluded.

Because of the large number of studies on PLHIV, an algorithm for study inclusion was developed. Where multiple studies existed for one country, all large, multicentre studies were included and studies conducted in single centres were excluded. If only smaller, less representative studies existed for a certain country, these were all included. This exclusion criterion was not applicable for PLHIV subgroups with multiple risks, e.g. MSM living with HIV. Estimates from populations with multiple risks reported by larger studies on PLHIV or people in prison were included if the sample size was greater than 50 or if they were from populations for which limited sample sizes are expected such as transgender persons, sex workers and intranasal drug users. The full inclusion and exclusion criteria are listed in Table 1.

**Table 1:** Inclusion and exclusion criteria for a systematic review of hepatitis B and C virus prevalence and incidence in key population groups with multiple risk factors, EU/EEA, 2017

Category	Inclusion criteria	Exclusion criteria
Study design/type	• Surveillance studies • RCTs • Non-randomised, prospective comparative studies • Prospective observational studies • Retrospective observational studies • Cross-sectional studies • Meta-analysis or systematic review	• Narrative review • Case reports, outbreak investigations • Non-pertinent publication types (e.g. expert opinions, letters to the editor, editorials, comments) • Animal studies • Laboratory studies (e.g. genetic, biochemistry or molecular studies) • Mathematical modelling studies
Country	• EU/EEA countries	• Other countries
Study subject	• Hepatitis B or C infection	• Other or unspecified hepatitis
Study population	• PLHIV in representative studies^a^ • People in prison • PLHIV or people in prison with additional risk factors (e.g. MSM living with HIV)	• Other populations • PLHIV if studies with more representative PLHIV populations exist for that country^a^ • Populations with multiple risks as part of a larger study within a single risk group, if the sample size was less than 50^b^ • Populations for which data on the same outcomes is available in a more recent publication
Outcomes	• Prevalence/incidence/proportion/ transmission rate of HBV/HCV in subpopulation or OR/RR of infection in subpopulation compared with general population • Outcome based on HBsAg, anti-HCV, HBV DNA or HCV RNA measurement in study population	• Other outcomes not related to risk of acquiring HBV/HCV or prevalence of disease • Outcome based on measurement of other virological markers, or if markers were not specified, self-reported infections

EU/EEA: European Union/European Economic Area; HBV: hepatitis B virus; HCV: hepatitis C virus; MSM: men who have sex with men; PLHIV: people living with HIV; OR: odds ratio; RCT: randomised controlled trial; RR: relative risk.

^a^ Because of the large number of studies performed in PLHIV populations, an algorithm for study inclusion was developed. Where multiple studies existed for one country, only large, representative nationwide studies were included. If only smaller, less representative studies existed for a country, these were included. This exclusion criterion was not applicable for PLHIV subgroups with multiple risks.

^b^ This criterion was not applicable to the subgroups for which limited sample sizes were expected, such as transgender persons, sex workers and intranasal drug users.

For the search in Embase, publication type was limited to ‘review’, ‘article’ and ‘article in press’. Only original research articles, i.e. not reviews, were included in this review, however, the reference lists of relevant systematic reviews retrieved in the literature search were checked manually for additional original articles not captured by the literature search. Additional articles captured were subject to the same inclusion and exclusion listed above.

Two reviewers, LM and UP, independently reviewed the title and abstract of retrieved publications. First, a random sample of 5% was screened in duplicate, the results were compared and used to refine the inclusion and exclusion criteria. Further rounds of duplicate review were conducted until a level of concordance of more than 95% was achieved, after which the remaining publications were divided between reviewers and screening continued independently in EndNote. The full texts of selected articles were subsequently screened by two reviewers, LM and UP, of which a random sample of 20% were screened in duplicate and which reached more than 95% concordance. The remaining 80% of publications were divided between reviewers and screened independently. For both screening steps, when cases of uncertainty about inclusion or exclusion were not resolved after discussion, articles were included.

### Definitions

HBV infection was defined as the presence of HBsAg or HBV DNA in serum, dried blood spot or saliva samples. For HCV infection, selected biomarkers were anti-HCV or HCV RNA. PLHIV were defined as people infected with HIV-1 or HIV-2, including PLHIV naive to treatment or currently/previously on treatment. People in prison were defined as people who are in any form of detention or penitentiary facility, including people in pre-trial centres, prison for convicted crimes, centres for juvenile offenders and other correctional facilities, excluding migrant detention centres. Individuals with a history of imprisonment, ex-prisoners, were also included.

### Data extraction and quality assessment

Relevant data were extracted from included articles and recorded in a data extraction file in Microsoft Excel. A predefined set of variables covering study characteristics, sampling, laboratory testing, study population details and outcomes was extracted per study. The complete list of variables is provided in Supplementary Table S1. Prevalence data on people in prison from the previous systematic review on hepatitis B and C prevalence in the EU/EEA were extracted directly into summary tables. No other variables were extracted.

The unit for data extraction was study, not article. A study is defined as a report of prevalence and/or incidence data on HBV infection and/or HCV infection for a defined population group, in a defined country, over a discrete period of time. If a study was captured by two different articles, the study was extracted once and the article with the most detail used as a reference.

The quality of all included articles was assessed. None of the included articles concerned studies with designs that can be critically appraised using standard checklists such as those available from the Scottish Intercollegiate Guidelines Network (SIGN) [25]. For this reason, relevant aspects from standard checklists were used to compile a yes/no checklist that was applied to each study (Supplementary Table S2) regarding the level of detail and clarity of the study, appropriateness of study population, data collection and denominator, and the representativeness of the sample. Because it was not possible to calculate an overall quality score for studies, all relevant articles were included regardless of their quality. However, all results of the quality assessment were taken into consideration in interpreting the results. Articles were excluded when the methods and/or results provided an insufficient level of details making it impossible to accurately extract data.

A set of detailed summary tables were developed per population group of interest, virus and outcome (Supplementary Tables S3–S9), and contained the following information: study reference, country, study period, sampling approach, study design, population subgroup, study population and sample size, results (prevalence/incidence), critical appraisal and comments.

### Data analysis

HBsAg and anti-HCV prevalence data were analysed qualitatively and compared with prevalence estimates in the general population and/or pregnant women (estimates derived from antenatal screening were considered proxy for general population in the absence of other data) of EU/EEA countries, reported in a recent systematic review conducted by ECDC [3], as well as to the 2% threshold for intermediate/high prevalence proposed by the WHO [26]. No meta-analysis was performed due to the heterogeneity of included studies.

## Results

The literature search retrieved 5,511 records. A total of 545 full text articles were assessed for eligibility after the title and abstract screening, of which 52 with data on PLHIV or people in prison were included (Figure). The included articles generated a total of 176 estimates of prevalence or incidence of HBV or HCV infection in EU/EEA countries. Detailed information on each included study is presented in Supplementary Tables S3–S9.

**Figure:**
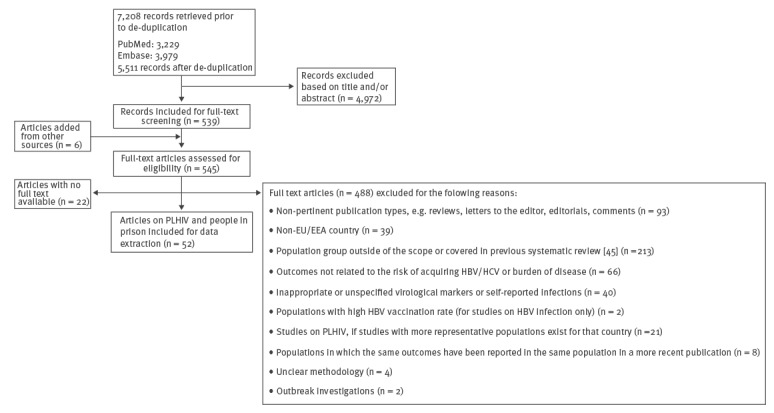
PRISMA flow diagram for the systematic review of hepatitis B and C virus prevalence and incidence in key population groups with multiple risk factors, EU/EEA, 2017

EU/EEA: European Union/European Economic Area; HBV: hepatitis B virus; HCV: hepatitis C virus; PLHIV: people living with HIV; PRISMA: Preferred Reporting Items for Systematic Reviews and Meta-Analyses.

### People living with HIV

The systematic literature search yielded 97 estimates in total on PLHIV providing data on HBV infection from nine EU/EEA countries and data on HCV infection from 14 EU/EEA countries. The majority of studies had at least one quality issue, with the most common issues being doubts as to whether the study population was representative of the source population, a lack of detail regarding selection of the study population and limited clarity and depth of methods, in particular regarding serological testing.

#### Prevalence estimates

Twenty-four HBV prevalence estimates were retrieved (Table 2). Of these, 11 were estimates on a broad group of PLHIV, ranging from 2.9% to 43.4%; five on MSM living with HIV, ranging from 1.7% to 17.2%; three on PWID living with HIV, ranging from 7.5% to 20.6%; three on PLHIV in prison, ranging from 6.8% to 16.9%; one on migrants living with HIV of 5.4% and one on haemophiliacs living with HIV of 5.9%. Each estimate was higher than the general population prevalence estimates where they were available for that country.

**Table 2:** HBV infection prevalence among people living with HIV by EU/EEA country and risk category, 2017

Country	PLHIV			MSM living with HIV			PWID living with HIV			PLHIV in prison			Migrants living with HIV			Haemophiliacs living with HIV		
	Number of studies	Prevalence (%)	Ref	Number of studies	Prevalence (%)	Ref	Number of studies	Prevalence (%)	Ref	Number of studies	Prevalence (%)	Ref	Number of studies	Prevalence (%)	Ref	Number of studies	Prevalence (%)	Ref
Bulgaria	1	10.4	[59]	1	8.4	[59]	1	20.6	[59]	1	16.9	[59]	0	NA	NA	0	NA	NA
Denmark	1	3	[60]	0	NA	NA	0	NA	NA	0	NA	NA	0	NA	NA	0	NA	NA
France	1	7	[61]	1	9.2	[61]	1	7.5	[61]	0	NA	NA	0	NA	NA	1	5.9	[61]
Germany	0	NA	NA	1	1.7	[62]	0	NA	NA	0	NA	NA	0	NA	NA	0	NA	NA
Greece	1	12.1	[63]	1	17.2	[63]	0	NA	NA	0	NA	NA	0	NA	NA	0	NA	NA
Italy	1	3.7	[64]	0	NA	NA	0	NA	NA	2	6.8–8.8	[65,66]	0	NA	NA	0	NA	NA
Netherlands	1	5	[67]	0	NA	NA	0	NA	NA	0	NA	NA	0	NA	NA	0	NA	NA
Romania	1	43.4	[27]	0	NA	NA	0	NA	NA	0	NA	NA	0	NA	NA	0	NA	NA
Spain	3	2.9–5.8	[68-70]	1	5.8	[68]	1	7.8	[68]	0	NA	NA	1	5.4	[71]	0	NA	NA
United Kingdom	1	5.1	[72]	0	NA	NA	0	NA	NA	0	NA	NA	0	NA	NA	0	NA	NA
**EU/EEA**	**11**	**2.9–43.4**	**–**	**5**	**1.7–17.2**	**–**	**3**	**7.5–20.6**	**–**	**3**	**6.8–16.9**	**–**	**1**	**5.4**	**–**	**1**	**5.9**	**–**

EU/EEA: European Union/European Economic Area; HBV: hepatitis B virus; MSM: men who have sex with men; NA: not available; PLHIV: people living with HIV; PWID: people who inject drugs; Ref: reference.

Where several studies were available, respective prevalence ranges are presented.

A total of 47 HCV prevalence estimates were retrieved (Table 3). Of these, 17 were on a broad group of PLHIV, ranging from 1.8% to 71.1%; 16 on MSM living with HIV, ranging from 0.9% to 25%; eight on PWID living with HIV, ranging from 38.3% to 98%; four on PLHIV in prison, ranging from 55.9% to 93.5%; one on migrants living with HIV of 7.7% and one on haemophiliacs living with HIV of 47.1%. Each estimate was higher than the general population estimate for that country, with two exceptions: one study on HIV-positive adolescents from Romania [27] and one study on MSM living with HIV from the United Kingdom (UK) [28]. In the Romanian study, the reported prevalence in HIV-positive adolescents was higher than the prevalence measured in HIV-negative controls (1.8% vs 0.8%), but both were lower than the general population estimate of 3.2% [27]. The study from the UK reported 0.9% anti-HCV positivity in HIV-positive MSM unaware of their HCV status, which was comparable to the general population estimate of 0.9% [28].

**Table 3:** HCV infection prevalence among people living with HIV by EU/EEA country and risk category, 2017

Country	PLHIV			MSM living with HIV			PWID living with HIV			PLHIV in prison			Migrants living with HIV			Haemophiliacs living with HIV		
	Number of studies	Prevalence (%)	Ref	Number of studies	Prevalence (%)	Ref	Number of studies	Prevalence (%)	Ref	Number of studies	Prevalence (%)	Ref	Number of studies	Prevalence (%)	Ref	Number of studies	Prevalence (%)	Ref
Bulgaria	1	25.6	[59]	1	3	[59]	1	87.4	[59]	1	82	[59]	0	NA	NA	0	NA	NA
Denmark	1	7	[60]	1	4	[60]	0	NA	NA	0	NA	NA	0	NA	NA	0	NA	NA
France	2	5.7–24.3	[61,73]	1	3.1	[61]	1	92.8	[61]	0	NA	NA	0	NA	NA	1	47.1	[61]
Germany	0	NA	NA	1	8.2	[62]	0	NA	NA	0	NA	NA	0	NA	NA	0	NA	NA
Greece	1	8.2	[63]	1	8.6	[63]	0	NA	NA	0	NA	NA	0	NA	NA	0	NA	NA
Ireland	1	26	[74]	0	NA	NA	0	NA	NA	0	NA	NA	0	NA	NA	0	NA	NA
Italy	1	40.7	[64]	0	NA	NA	0	NA	NA	2	55.9–78.3	[65,66]	0	NA	NA	0	NA	NA
Netherlands	1	3.7	[67]	5^a^	10.3–25	[75,76]	0	NA	NA	0	NA	NA	0	NA	NA	0	NA	NA
Poland	1	71.1	[67]	0	NA	NA	1	97.7	[77]	1	93.5	[77]	0	NA	NA	0	NA	NA
Romania	1	1.8	[27]	0	NA	NA	0	NA	NA	0	NA	NA	0	NA	NA	0	NA	NA
Slovenia	1	7.6	[78]	0	NA	NA	0	NA	NA	0	NA	NA	0	NA	NA	0	NA	NA
Spain	4	20.5–61	[68-70,79]	2	3.5–4.3	[68,70]	2	89.5–91.4	[68,70]	0	NA	NA	1	7.7	[71]	0	NA	NA
Sweden	1	14	[80]	1	3.7	[80]	1	98	[80]	0	NA	NA	0	NA	NA	0	NA	NA
United Kingdom	1	8.9	[81]	3	0.9–7.2	[28,81,82]	2	38.3–83.7	[81,83]	0	NA	NA	0	NA	NA	0	NA	NA
**EU/EEA**	**17**	**1.8–71.1**	**–**	**16**	**0.9–25**	**–**	**8**	**38.3–98**	**–**	**4**	**55.9–93.5**	**–**	**1**	**7.7**	**–**	**1**	**47.1**	**–**

EU/EEA: European Union/European Economic Area; HCV: hepatitis C virus; MSM: men who have sex with men; NA: not available; PLHIV: people living with HIV; PWID: people who inject drugs; Ref: reference.

^a^ Including one study of MSM with chlamydia living with HIV (prevalence: 25%) and one study of MSM sex workers living with HIV (prevalence: 13%).

Where several studies were available, respective prevalence ranges are presented.

#### Incidence estimates

Eight HBV infection incidence estimates were retrieved (Table 4). These included four on a broad group of PLHIV, ranging from 0.0 to 2.5 cases per 100 person-years (PY); three on MSM living with HIV, ranging from 1.1 to 2.5 cases per 100 PY and one on sexually transmitted infection (STI)-infected PLHIV of 1.3 cases per 100 PY. For HCV infection, eighteen incidence estimates were retrieved (Table 5). This included five on a broad group of PLHIV, which ranged from 0.3 to 0.9 cases per 100 PY; 12 on MSM living with HIV, ranging from 0.7 to 2.4 cases per 100 PY and one on PWID living with HIV of 7.2 cases per 100 PY.

**Table 4:** HBV infection incidence among people living with HIV by EU/EEA country and risk category, cases per 100 person-years, 2017

Country	PLHIV			MSM living with HIV			STI infected PLHIV		
	Number of studies	Incidence (cases/100 PY)	Ref	Number of studies	Incidence (cases/100 PY)	Ref	Number of studies	Incidence (cases/100 PY)	Ref
Denmark	1	0.0	[60]	0	NA	NA	0	NA	NA
Germany	0	NA	NA	1	2.5	[62]	0	NA	NA
Italy	1	1.2	[84]	1	1.7	[84]	1	1.3	[84]
Netherlands	0	NA	NA	1	1.1	[85]	0	NA	NA
Romania	1	2.5	[27]	0	NA	NA	0	NA	NA
United Kingdom	1	1.7	[72]	0	NA	NA	0	NA	NA
**EU/EEA**	**4**	**0.0–2.5**	**–**	**3**	**1.1–2.5**	**–**	**1**	**1.3**	**–**

EU/EEA: European Union/European Economic Area; HBV: hepatitis B virus; MSM: men who have sex with men; NA: not available; PLHIV: people living with HIV; PY: person-years; Ref: reference; STI: sexually transmitted infection.

Where several studies were available, respective incidence ranges are presented.

**Table 5:** HCV infection incidence among people living with HIV by EU/EEA country and risk category, cases per 100 person-years, 2017

Country	PLHIV			MSM living with HIV			PWID living with HIV		
	Number of studies	Incidence (cases/100 PY)	Ref	Number of studies	Incidence (cases/100 PY)	Ref	Number of studies	Incidence (cases/100 PY)	Ref
Belgium	0	NA	NA	2	1.4**^a^**	[86,87]	0	NA	NA
Denmark	1	0.3	[60]	1	^b^	[88]	0	NA	NA
France	1	0.4	[73]	1	^c^	[89]	0	NA	NA
Germany	0	NA	NA	1	1.5	[62]	0	NA	NA
Italy	1	0.6	[90]	1	0.7	[90]	1	7.2	[90]
Netherlands	0	NA	NA	2	1.1–2.4	[76,91]	0	NA	NA
Spain	1	0.9	[92]	1	0.8	[92]	0	NA	NA
United Kingdom	0	NA	NA	3	0.9–1.1	[93-95]	0	NA	NA
**EU/EEA**	**5^d^**	**0.3–0.9**	**[** 96 **]**	**12**	**0.7–2.4**	**–**	**1**	**7.2**	**–**

EU/EEA: European Union/European Economic Area; HCV: hepatitis C virus; MSM: men who have sex with men; NA: not available; PLHIV: people living with HIV; PWID: people who inject drugs; PY: person-years; Ref: reference.

**^a^** One of the two studies from Belgium provided an estimate of 2.3–2.9% per year [87].

^b^ The study from Denmark provided an estimate of 0.4% per year [88].

^c^ The study from France provided an estimate of 0.4% per year [89].

^d^ An additional study gives an estimate for an EU/EEA-wide, 12-year cumulative incidence of 4.4% [96].

Where several studies were available, respective incidence ranges are presented.

### People in prison

Fifty-eight estimates on people in prison were extracted from the previous ECDC systematic review on hepatitis B and C prevalence in the EU/EEA [24]; 15 on HBV infection prevalence from 12 EU/EEA countries and 43 on HCV infection prevalence from 12 EU/EEA countries. Twenty-eight estimates of prevalence or incidence were retrieved in the current systematic literature search, including eight on HBV infection from four EU/EEA countries and 20 on HCV infection from eight EU/EEA countries. Quality issues were noted for a majority of studies. The most commonly noted issues were doubts as to whether the study population was representative of the source population and a lack of detail describing the selection of the study population.

#### Prevalence estimates

Twenty-three HBV infection prevalence estimates were retrieved in total (Table 6); 16 on a broad group of people in prison (ranging from 0.0% to 25.2%); three on PLHIV in prison (ranging from 6.8% to 16.9%); one on PWID in prison (1.4%); two on people in prison with tattoos (1.4% and 2.3%); and one among people in prison who had unprotected sex (1.4%). For the broad group of people in prison, prevalence estimates from Hungary, Italy, Spain, the UK and Romania were slightly higher than those of general population. Estimates from Croatia, France, Ireland and the UK were in line with general population estimates.

**Table 6:** HBV infection prevalence among people in prison by EU/EEA country and risk category, 2017

Country	People in prison			PLHIV in prison			PWID in prison			People in prison with tattoos			People in prison who have had unprotected sex		
	Number of studies	Prevalence (%)	Ref	Number of studies	Prevalence (%)	Ref	Number of studies	Prevalence (%)	Ref	Number of studies	Prevalence (%)	Ref	Number of studies	Prevalence (%)	Ref
Bulgaria	1	25.2	[24]	1	16.9	[59]	0	NA	NA	0	NA	NA	0	NA	NA
Croatia	3	1.3–1.4	[24]	0	NA	NA	0	NA	NA	0	NA	NA	0	NA	NA
Finland	1	0.5	[24]	0	NA	NA	0	NA	NA	0	NA	NA	0	NA	NA
France	2	0.6	[24,97]	0	NA	NA	0	NA	NA	0	NA	NA	0	NA	NA
Hungary	1	1.5	[24]	0	NA	NA	1	1.4	[98]	2^a^	1.4–2.3	[98]	1	1.4	[98]
Ireland	1	0.3	[24]	0	NA	NA	0	NA	NA	0	NA	NA	0	NA	NA
Italy	1	6.7	[24]	2	6.8–8.8	[65,66]	0	NA	NA	0	NA	NA	0	NA	NA
Luxembourg	1	7.0	[24]	0	NA	NA	0	NA	NA	0	NA	NA	0	NA	NA
Portugal	1	10.8	[24]	0	NA	NA	0	NA	NA	0	NA	NA	0	NA	NA
Romania	1	10.7	[24]	0	NA	NA	0	NA	NA	0	NA	NA	0	NA	NA
Spain	1	2.6	[24]	0	NA	NA	0	NA	NA	0	NA	NA	0	NA	NA
United Kingdom	2	0.0–2.0	[24]	0	NA	NA	0	NA	NA	0	NA	NA	0	NA	NA
**EU/EEA**	**16**	**0.0–25.2**	**–**	**3**	**6.8–16.9**	**–**	**1**	**1.4**	**–**	**2**	**1.4–2.3**	**–**	**1**	**1.4**	**–**

EU/EEA: European Union/European Economic Area; HBV: hepatitis B virus; NA: Not available; PLHIV: people living with HIV; PWID: people who inject drugs; Ref: reference.

^a^ Including one study on people who got a tattoo in prison (prevalence: 2.3%) and one study on people in prison with tattoos (prevalence: 1.4%).

Where several studies were available, respective prevalence ranges are presented.

A total of 59 estimates were retrieved on HCV infection prevalence (Table 7); 44 on a broad group of people in prison, ranging from 1.3% to 86.3%; five on PWID in prison, ranging from 22.5% to 86%; four on PLHIV in prison, ranging from 55.9% to 93.5%; three on people in prison with tattoos, ranging from 4.5% to 51.2%; two on people in prison who have had unprotected sex of 4.2% and 43.2% and one on people in prison who have had blood transfusions of 48.7%. Each estimate was higher than the general population prevalence estimates where they were available for that country.

**Table 7:** HCV infection prevalence among people in prison by EU/EEA country and risk category, 2017

Country	People in prison			PLHIV in prison			PWID in prison			People in prison with tattoos			People in prison who have had unprotected sex			People in prison who have had transfusions		
	Number of studies	Prevalence (%)	Ref	Number of studies	Prevalence (%)	Ref	Number of studies	Prevalence (%)	Ref	Number of studies	Prevalence (%)	Ref	Number of studies	Prevalence (%)	Ref	Number of studies	Prevalence (%)	Ref
Bulgaria	3	20.5–28.6	[24]	1	82	[59]	0	NA	NA	0	NA	NA	0	NA	NA	0	NA	NA
Croatia	3	4.3–14.2	[24]	0	NA	NA	0	NA	NA	0	NA	NA	0	NA	NA	0	NA	NA
Finland	1	45.8	[24]	0	NA	NA	0	NA	NA	0	NA	NA	0	NA	NA	0	NA	NA
France	8	3.8–6.8	[24,97]	0	NA	NA	0	NA	NA	0	NA	NA	0	NA	NA	0	NA	NA
Germany	4	8.6–84.9	[24]	0	NA	NA	0	NA	NA	0	NA	NA	0	NA	NA	0	NA	NA
Hungary	1	4.9	[24]	0	NA	NA	1	22.5	[98]	2	4.5–4.6	[98]	1	4.2	[98]	0	NA	NA
Ireland	1	12.9	[24]	0	NA	NA	0	NA	NA	0	NA	NA	0	NA	NA	0	NA	NA
Italy	2	37.4–38	[24]	2	55.9–78.3	[66]	1	74.7	[99]	1	51.2	[99]	1	43.2	[99]	1	48.7	[99]
Luxembourg	1	86.3	[24]	0	NA	NA	0	NA	NA	0	NA	NA	0	NA	NA	0	NA	NA
Norway	0	NA	NA	0	NA	NA	1	86	[100]	0	NA	NA	0	NA	NA	0	NA	NA
Poland	0	NA	NA	1	93.5	[77]	0	NA	NA	0	NA	NA	0	NA	NA	0	NA	NA
Portugal	2	10.8–34.4	[24]	0	NA	NA	0	NA	NA	0	NA	NA	0	NA	NA	0	NA	NA
Spain	13	14.7–44.9	[24]	0	NA	NA	1	84.9	[101]	0	NA	NA	0	NA	NA	0	NA	NA
United Kingdom	5	1.3–19.2	[24]	0	NA	NA	1	49.3	[30]	0	NA	NA	0	NA	NA	0	NA	NA
**EU/EEA**	**44**	**1.3–86.3**	**–**	**4**	**55.9–93.5**	**–**	**5**	**22.5–86**	**–**	**3**	**4.5–51.2**	**–**	**2**	**4.2–43.2**	**–**	**1**	**48.7**	**–**

EU/EEA: European Union/European Economic Area; HCV: hepatitis C virus; MSM: men who have sex with men; NA: not available; PLHIV: people living with HIV; PWID: people who inject drugs; Ref: reference.

Where several studies were available, respective prevalence ranges are presented.

#### Incidence estimates

Four incidence estimates were retrieved, two from Spain and the UK, respectively, all of which were on HCV infection. In Spain, the reported incidence was 1.2 cases per 100 PY in people in prison [29] and 6.7 cases per 100 PY in people in prison with a history of intravenous drug use [29]. In the UK, the reported incidence was 1 case per 100 PY among people in prison [30] and 2.0–2.9% among people in prison who inject drugs [30].

## Discussion

In this systematic review, evidence on HBV/HCV infection prevalence and incidence in PLHIV and people in prison in EU/EEA countries was compiled and compared with estimates from the general population in order to assess the extent of risk and prevalence that these groups have of HBV and HCV infection. To our knowledge, this is the first systematic review covering both HBV and HCV epidemiology among these groups in the EU/EEA.

Estimates of HBV infection prevalence were higher for PLHIV, including all PLHIV groups with multiple risks, than those reported for the general population [3] in every country for which data were available. The highest estimates were from Romania and Greece, which are among EU/EEA countries with the highest HBV infection prevalence among the general population [3]. Although there are no recent estimates of prevalence in the general population for Bulgaria, given the high prevalence of 3.2% HBsAg among first time blood donors noted by Hofstraat et al., the prevalence in the general population is likely to be higher than the 0.9% prevalence of the EU/EEA as a whole [3]. This may explain the high prevalence reported in PLHIV, 10.4%. Similarly, estimates of HCV infection prevalence were higher for PLHIV, including all PLHIV groups with multiple risks, than general population estimates [3] in every country with data on both, with the exception of the two estimates among HIV-positive adolescents from Romania [27], and among HIV positive MSM unaware of their HCV status in the UK, which was similar to the general population estimate [28]. This evidence indicates that a high prevalence of HBV and HCV infection exists in PLHIV across the EU/EEA. In general, HCV infection prevalence estimates were much higher than those for HBV infection, most strikingly among PWID living with HIV. This is possibly because of increased transmission efficiency of HCV in the presence of HIV [14], increased transmission efficiency through injecting drug use, and/or reflecting the impact of HBV vaccination programmes. However, data on HBV vaccination coverage among PWID or PLHIV are hardly available in the EU/EEA, with few exceptions [31,32], making it hard to assess the relative effect of this prevention measure across countries and different birth cohorts.

The evidence retrieved on HBV infection prevalence for people in prison was more heterogeneous. Although a number of studies (from Bulgaria, Italy, Luxembourg, Portugal and Romania) reported estimates of prevalence considerably higher than that of the general population, many estimates were more in line with countries’ general population estimates [3]. Differences in prevalence estimates between studies may be because of variations in the demographic composition of the study populations such as the proportion of PWID or PLHIV or prisoners born in endemic countries [18,33]. Vaccination policies in different countries or prisons are also likely to impact the prevalence of HBV infection. Universal routine HBV vaccination in childhood is currently implemented in the majority of EU countries, and increasing vaccine coverage in countries has been significantly associated with decreasing acute HBV notification rates [34]. However, depending on the year when a vaccination programme has been introduced in a country, this may have had a different impact on the cohorts of individuals entering into prison. Targeted vaccination for people in prison has been implemented in at least some EU/EEA countries [29]. A recent analysis of the impact of such a programme in Scotland has shown very promising results, with vaccination against HBV being associated with reduced odds of having an HBV infection among PWID [32]. In addition, there may be considerable variation in prevalence between individual prisons, because of, for example, the unique socio-demographic mix of the local prison population in terms of age distribution, vaccination coverage, and proportion and origin of migrant subpopulation.

HCV infection prevalence estimates for people in prison, including people in prison with multiple risks, were uniformly higher than that of the general population and were also high in countries for which no comparative general population prevalence data were available. This suggests that HCV infection prevalence is high in people in prison across the EU/EEA.

The highest levels of HCV infection prevalence were reported among population groups with multiple risk factors, especially among PWID living with HIV, PWID in prison and PLHIV in prison. In these studies, anti-HCV positivity was present in more than half of the population. Furthermore, all estimates were higher than those reported for the broader population groups of PLHIV or people in prison in the same country, indicating that a very high prevalence of disease exists in these intertwined groups with concomitant, multiple risks. Although fewer studies were retrieved with data on HBV infection prevalence in populations with multiple risk factors, for several countries, the available evidence suggests that the prevalence of HBV may also be slightly higher in PLHIV with multiple risks than the broader PLHIV population. The prevalence of HIV is high among PWID and people in prison, with the latter partly a reflection of the fact that PWID are over-represented among prison populations [35,36]. As transmission routes overlap between blood-borne viruses (BBVs), PWID or people in prison already infected with HIV are likely to have also been exposed to HBV and HCV. Continuing or initiating drug use is reported to occur in prisons and poses an extra risk of transmission because of a lack of access to sterile injecting equipment in most prisons in the EU/EEA [37-39].

Our findings on HCV infection prevalence in PLHIV are in agreement with previous systematic reviews on HCV infection in PLHIV. Platt et al. found evidence that HCV infection prevalence is greater among PLHIV than people without HIV in regions worldwide, including Europe. The greatest prevalence was found among PWID living with HIV [40]. Cacoub et al. reviewed results of surveys conducted on PLHIV in France between 2003 to 2012 and reported high HCV prevalence in this population, with the highest HCV prevalence reported among PWID living with HIV. However, HCV prevalence decreased over time from 22–24% to 16–18%, particularly among PWID living with HIV, although an increase in prevalence was reported among MSM living with HIV [41]. Our findings on HCV infection prevalence in people in prison are also consistent with previous systematic reviews that reported high prevalence in prisons in European regions or countries, and an overlap between HCV-positivity and injecting drug use among people in prison [37,42-45].

Less data were available for incidence than prevalence, especially for people in prison, for which only HCV infection incidence data were retrieved. This is likely due to the greater challenge of undertaking an incidence study. Incidence estimates from a number of countries indicated that new HBV and HCV infections are occurring in PLHIV, particularly in MSM living with HIV. The highest HBV infection incidence estimate among PLHIV was reported in a study conducted in Romania, which also had the highest reported HBV infection prevalence estimate. For people in prison, the retrieved evidence indicates that new HCV infections are occurring among people in prison, particularly among those using intravenous drugs. Other systematic reviews have found evidence of incident HCV infection occurring in prisons, particularly in prisoners with a history of injecting drugs [42,44,45].

The recently released WHO testing guidance for hepatitis B and C proposes a 2% threshold for HBV/HCV infection prevalence, above which testing scale-up is recommended [26]. All reported HBV and HCV infection prevalence estimates for PLHIV, excluding outliers, all but one HCV infection prevalence estimates for prisoners and approximately half of HBV infection prevalence estimates for people in prison retrieved by this review were above this threshold. This presents a strong case for increasing testing coverage in these populations. Diversifying testing approaches, providing decentralised hepatitis C care after diagnosis and extending it to community-based providers is essential to improving testing coverage and bridge gaps in the care cascade for affected populations. Particularly among those who belong to underserved communities this will ensure maximum benefit for individuals as well as at a public health level [46-48]. Alongside this, harm reduction initiatives such as safe injection sites and syringe exchange programmes are important strategies that can be implemented to reduce transmission of HBV and HCV in PWID living with HIV and/or in prison [49]. Harm reduction initiatives have been shown to be effective in preventing the spread of BBVs both in the community and in prison [50], although their impact is influenced by the level of coverage and quality of the services, which is less easily monitored [51].

At present, the policy on testing of PLHIV and people in prison varies across EU/EEA countries, with few reporting that testing is offered to all prisoners, and around a third reporting that testing is offered only on the basis of risk factors or medical reasons, with limited information on the level of coverage [10,52]. Recently released EU guidance on active case finding in prisons suggests that a diagnostic test for HBV, HCV and HIV should be actively offered to all people in detention [53]. The systematic implementation of such an approach, coupled with adequate linkage to care and treatment across the EU/EEA, would likely contribute to reducing transmission and burden of disease, as suggested by recent studies [32,54,55]. Monitoring testing efforts and their impact should be integrated in the national and regional initiatives to track progress towards the goals and targets outlined in the Global Health Sector Strategy on Viral Hepatitis and the WHO European Region action plan for the health sector response to viral hepatitis [22].

The high prevalence of co-infection with HBV and HCV among PLHIV is reflected in the existing recommendation by the European AIDS Clinical Society to test individuals diagnosed with HIV for HBV and HCV infection [56]. However, the level of implementation of co-infection testing approach across the EU/EEA may be suboptimal [57], making it likely that opportunities for earlier diagnosis are missed in a population group that is already in contact with the health services. This highlights the importance of promoting an integrated approach to prevention for those accessing services, one that brings clinical and public health/community services together focusing on the individual at the point of access to care to stem off further transmission. ECDC has recently launched public health guidance on integrated testing of HBV, HCV and HIV in the attempt to foster change in the EU/EEA [58].

This study has some limitations. Retrieved estimates were not distributed evenly throughout the EU/EEA, with a disproportional number of studies retrieved from Spain, Italy and the UK, while for several EU/EEA countries, no studies were found. Direct comparison of prevalence or incidence data was limited by the large degree of heterogeneity between studies and population groups. For example, the proportion of the population vaccinated for HBV varied between studies and often was not mentioned. Sampling methods, laboratory testing methods used and use of confirmatory tests differed between studies. In addition, many estimates were based on single centres or local rather than national data, particularly for studies on people in prison, and studies with small sample sizes. Some studies used convenience sampling which may have resulted in a biased result. We did not perform a formal risk of bias assessment, however, quality issues were recorded per study. Noted quality issues common to many retrieved studies included the lack of a representative study sample and insufficient detail reported on serology and sampling methods. We did not contact contact authors to try to obtain missing information. Differences between the studies retrieved with regard to HBV/HCV infection prevalence or incidence may be partly explained by these factors. For this reason, grouped data are presented here as ranges and no weighted or pooled average was calculated. In addition, general population prevalence data was partially based on estimates from pregnant women, which may not be a reliable proxy for the general population, and likely underestimates anti-HCV prevalence [3].

Robust epidemiological studies providing data around the prevalence of HBV and HCV infections are lacking. Prevalence and incidence estimates could not be retrieved for all EU/EEA countries and population groups of interest. However, studies are needed to provide estimates to inform the design of national and local programmes. The identification of groups and subgroups at increased risk is key to ensuring appropriate prioritisation for resource allocation and intervention implementation. There have been several initiatives to strengthen the abilty to identify such groups, including a programme of work to improve the quality of notification-based surveillance systems of newly diagnosed hepatitis infections and recently-released ECDC guidance on infectious disease prevention and control in PWID and in prison settings, and HIV/HBV/HCV testing to support countries in implementing adequate and evidence-based interventions [49,53,58].

In conclusion, our findings demonstrate that a high prevalence of HBV and HCV infection exists in PLHIV and people in prisons throughout the EU/EEA, particularly in those with multiple and overlapping risk factors. Furthermore, the evidence suggests that these populations may be at ongoing risk. This indicates that considerable gaps exist in prevention efforts and that there is a need to scale up testing in order to identify and link these cases with care, and to integrate efforts across HIV and hepatitis services.

## Supplementary Data

1280000 Supplementary Material
