# Supplementary material for: Hepatitis B and C prevalence and incidence in key population groups with multiple risk factors in the EU/EEA: a systematic review
Source: Euro Surveill. 2019 Jul 25;24(30):1800614. doi: 10.2807/1560-7917.ES.2019.24.30.1800614 (PMC6668290; doi:10.2807/1560-7917.ES.2019.24.30.1800614)
Supplement: Supplementary Material [file 1800614_MASON_HepBandC_Supplement.pdf]

Supplementary material

Hepatitis B and C prevalence and incidence in key population groups with multiple risk factors in the EU/EEA: a systematic review

This supplementary material is hosted by *Eurosurveillance* as supporting information alongside the article “Hepatitis B and C prevalence and incidence in key population groups with multiple risk factors in the EU/EEA: a systematic review” on behalf of the authors who remain responsible for the accuracy and appropriateness of the content. The same standards for ethics, copyright, attributions and permissions as for the article apply. *Eurosurveillance* is not responsible for the maintenance of any links or email addresses provided therein."

Supplement S1: Search strings

PubMed search strings

String for Hepatitis B and Hepatitis C virus

(Hepatitis B[MeSH] OR Hepatitis B virus[Mesh] OR Hepatitis B Antigens[Mesh] OR Hepatitis B Antibodies[Mesh] OR hepatitis b[tiab] OR hbv[tiab] OR Hep B[tiab] OR hbsag[tiab] OR “hbs ag”[tiab] OR Hepatitis C[MeSH] OR Hepacivirus[MeSH] OR Hepatitis C Antibodies[MeSH] OR Hepatitis C Antigens[Mesh] OR Hepatitis C[tiab] OR hepaciviru*[tiab] OR hcv[tiab] OR hep c[tiab] OR blood borne virus*[tiab] or bbv[tiab])

String for occurrence

(Seroepidemiologic Studies[Mesh] OR Prevalence[Mesh] OR Incidence[Mesh] OR seroprevalen*[tiab] OR prevalen*[tiab] OR inciden*[tiab] OR occurrence[tiab] OR positivity rate*[tiab] OR odds ratio*[tiab] OR relative risk*[tiab] OR transmission rate*[tiab] OR “at risk”[tiab])

String for risk groups not covered in previous reviews

(health care staff[tiab] OR healthcare staff[tiab] OR health staff[tiab] OR health worker*[tiab] OR health care worker*[tiab] OR healthcare worker*[tiab] OR health care provider*[tiab] OR healthcare provider*[tiab] OR medical staff[tiab] OR exposure prone procedure*[tiab] OR fieldworker*[tiab] OR safety worker*[tiab] OR police*[tiab] OR firefighter*[tiab] OR firemen[tiab] OR fireman[tiab] OR paramedic*[tiab] OR ambulance[tiab] OR corrections officer*[tiab] OR correctional officer*[tiab] OR prison guard*[tiab] OR waste worker*[tiab] OR waste dispos*[tiab] OR waste collect*[tiab] OR waste remov*[tiab] OR sewage worker*[tiab] OR rubbish men[tiab] OR rubbish man[tiab] OR rubbishm*[tiab] OR rubbish dispos*[tiab] OR rubbish collect*[tiab] OR rubbish remov*[tiab] OR bin man[tiab] OR bin men[tiab] OR dustbin man[tiab] OR dustbin men[tiab] OR garbage men[tiab] OR garbage man[tiab] OR garbagem*[tiab] OR garbage dispos*[tiab] OR garbage collect*[tiab] OR garbage worker*[tiab] OR garbage remov*[tiab] OR trash men[tiab] OR trash man[tiab] OR trashm*[tiab] OR trash dispos*[tiab] OR trash collect*[tiab] OR trash worker*[tiab] OR trash remov*[tiab] OR refuse dispos*[tiab] OR refuse collect*[tiab] OR refuse worker*[tiab] OR refuse remov*[tiab] OR HIV infected[tiab] OR HIV positive[tiab] OR HIV seropositive[tiab] OR HIV patient*[tiab] OR infected with HIV[tiab] OR STI patient*[tiab] OR STD patient*[tiab] OR STI infected[tiab] OR STD infected[tiab] OR intranasal drug*[tiab] OR snort drugs[tiab] OR inhale drugs[tiab] OR blood transfusion*[tiab] OR transplant*[tiab] OR dialys*[tiab] OR hemodialys*[tiab] OR haemodialys*[tiab] OR medical intervention*[tiab] OR dental[tiab] OR dentistry[tiab] OR surgery[tiab] OR medical procedure*[tiab] OR tattoo*[tiab] OR pierc*[tiab] OR scarifi*[tiab] OR body modification[tiab] OR acupunctur*[tiab] OR mesotherapy[tiab] OR care home*[tiab] OR institutionali*[tiab] OR residential care[tiab] OR mentally disabled[tiab] OR mental disabilit*[tiab] OR intellectual disabilit*[tiab] OR intellectually disabled[tiab] OR low intellect[tiab] OR learning disabilit*[tiab] OR retard*[tiab] OR cognitive disabilit*[tiab] OR intellectual impairment[tiab] OR mental deficiency[tiab] OR homeless*[tiab] OR vagrant*[tiab] OR partner*[tiab] OR couple*[tiab] OR spouse*[tiab] OR husband*[tiab] OR wife[tiab] OR wives[tiab] OR household[tiab] OR contact*[tiab] OR mother*[tiab] OR birth cohort[tiab] OR travel*[tiab] OR rape*[tiab] OR assault*[tiab] OR multiple sexual partners[tiab] OR multiple sex partners[tiab] OR multiple sexual contacts[tiab] OR sexually promiscuous[tiab] OR sexual promiscuity[tiab] OR diabet*[tiab] OR auto-inject*[tiab] OR transsexual*[tiab] OR transgender*[tiab] OR anabolic steroid*[tiab] OR performance enhancing[tiab] OR beauty[tiab] OR salon*[tiab] OR pedicur*[tiab] OR manicur*[tiab])

String for EU/EEA

(((((((Europe*[ad] OR Europa*[ad] OR EU[ad] OR EEA[ad] OR "EU/EEA"[ad] OR ECSC[ad] OR Euratom[ad] OR Eurozone[ad] OR EEC[ad] OR ec[ad] OR (Schengen[ad] AND (area[ad] OR countr*[ad] OR region*[ad] OR state[ad] OR states[ad])) OR Euroregion[ad] OR Euroregions[ad] OR Balkan[ad] OR Balkans[ad] OR Baltic[ad] OR (Mediterranean[ad] AND (area[ad] OR countr*[ad] OR region*[ad] OR state[ad] OR states[ad])) OR (Alpine[ad] AND (area[ad] OR countr*[ad] OR region*[ad] OR state[ad] OR states[ad])) OR Scandinavia[ad] OR Scandinavian[ad] OR "Nordic country"[ad] OR "Nordic countries"[ad] OR "Nordic state"[ad] OR "Nordic states"[ad] OR Danubian[ad] OR "Iberian peninsula"[ad] OR "Peninsula iberica"[ad] OR "Peninsule Iberique"[ad] OR "Iberiar Penintsula"[ad] OR Iberia[ad] OR Czechoslovakia[ad] OR "Czecho Slovakia"[ad] OR Ceskoslovensko[ad] OR "Cesko slovensko"[ad] OR Benelux[ad] OR Fennoscandia[ad] OR "Fenno Scandinavia"[ad] OR Fennoskandi*[ad] OR (Visegrad[ad] AND (Group[ad] OR Four[ad] OR Triangle[ad])) OR "Visegradska ctyrka"[ad] OR "Visegradska skupina"[ad] OR "Visegradi Egyuttmukodes"[ad] OR "Visegradi negyek"[ad] OR "Grupa Wyszehradzka"[ad] OR "Vysehradska skupina"[ad] OR "Vysehradska stvorka"[ad])) OR ("Iceland"[Mesh] OR Iceland[tw] OR Icelandic*[tw] OR islenska*[tw] OR Icelander*[tw] OR islendinga*[tw] OR Islendigar[tw] OR Inslenska[tw] OR Reykjavik[tw] OR Reykjavikurborg[tw] OR Hofudborgarsvaedi[tw] OR Sudurnes[tw] OR Vesturland[tw] OR Vestfirdir[tw] OR Westfjords[tw] OR Nordurland[tw] OR Austurland[tw] OR Sudurland[tw] OR Kopavogur[tw] OR Hafnarfjordur[tw] OR Akureyri[tw] OR Gardabaer[tw] OR Mosfellsbaer[tw] OR Keflavik[tw] OR Akranes[tw] OR Selfoss[tw] OR Seltjarnarnes[tw]) OR ((Iceland[ad] OR Icelandic*[ad] OR islenska*[ad] OR Icelander*[ad] OR islendinga*[ad] OR Islendigar[ad] OR Inslenska[ad] OR Reykjavik[ad] OR Reykjavikurborg[ad] OR Hofudborgarsvaedi[ad] OR Sudurnes[ad] OR Vesturland[ad] OR Vestfirdir[ad] OR Westfjords[ad] OR Nordurland[ad] OR Austurland[ad] OR Sudurland[ad] OR Kopavogur[ad] OR Hafnarfjordur[ad] OR Akureyri[ad] OR Gardabaer[ad] OR Mosfellsbaer[ad] OR Keflavik[ad] OR Akranes[ad] OR Selfoss[ad] OR Seltjarnarnes[ad]) OR (Norway[ad] OR Norwegian*[ad] OR Norge[ad] OR Noreg[ad] OR Norgga[ad] OR Akershus[ad] OR "Aust Agder"[ad] OR Buskerud[ad] OR Finnmark[ad] OR Hedmark[ad] OR Hordaland[ad] OR "More og Romsdal"[ad] OR "More and Romsdal"[ad] OR "More Romsdal"[ad] OR Nordland[ad] OR Trondelag[ad] OR Oppland[ad] OR Oslo[ad] OR Ostfold[ad] OR Rogaland[ad] OR "Sogn og fjordane"[ad] OR "Sogn and fjordane"[ad] OR "sogn fjordane"[ad] OR Telemark[ad] OR Troms[ad] OR Romsa[ad] OR Romssa[ad] OR "Vest Agder"[ad] OR Vestfold[ad] OR Bergen[ad] OR Stavanger[ad] OR Sandnes[ad] OR Trondheim[ad] OR Trondhjem[ad] OR Kaupangen[ad] OR Nidaros[ad] OR Drammen[ad] OR Fredrikstad[ad] OR Skien[ad] OR Tromso[ad] OR Sarpsborg[ad]) OR (Liechtenstein[ad] OR Lienchtensteiner*[ad] OR Balzers[ad] OR Eschen[ad] OR Gamprin[ad] OR Mauren[ad] OR Planken[ad] OR Ruggell[ad] OR Schaan[ad] OR Schellenberg[ad] OR Triesen[ad] OR Triesenberg[ad] OR Vaduz[ad]))) OR (Iceland[ad] OR Icelandic*[ad] OR islenska*[ad] OR Icelander*[ad] OR islendinga*[ad] OR Islendigar[ad] OR Inslenska[ad] OR Reykjavik[ad] OR Reykjavikurborg[ad] OR Hofudborgarsvaedi[ad] OR Sudurnes[ad] OR Vesturland[ad] OR Vestfirdir[ad] OR Westfjords[ad] OR Nordurland[ad] OR Austurland[ad] OR Sudurland[ad] OR Kopavogur[ad] OR Hafnarfjordur[ad] OR Akureyri[ad] OR Gardabaer[ad] OR Mosfellsbaer[ad] OR Keflavik[ad] OR Akranes[ad] OR Selfoss[ad] OR Seltjarnarnes[ad]) OR ((((("Liechtenstein"[Mesh] OR Liechtenstein[tw] OR Lienchtensteiner*[tw] OR Balzers[tw] OR Eschen[tw] OR Gamprin[tw] OR Mauren[tw] OR Planken[tw] OR Ruggell[tw] OR Schaan[tw] OR Schellenberg[tw] OR Triesen[tw] OR Triesenberg[tw] OR Vaduz[tw])) OR ("Norway"[Mesh] OR Norway[tw] OR Norwegian*[tw] OR Norge[tw] OR Noreg[tw] OR Norgga[tw] OR Akershus[tw] OR "Aust Agder"[tw] OR Buskerud[tw] OR Finnmark[tw] OR Hedmark[tw] OR Hordaland[tw] OR "More og Romsdal"[tw] OR "More and Romsdal"[tw] OR "More Romsdal"[tw] OR Nordland[tw] OR Trondelag[tw] OR Oppland[tw] OR Oslo[tw] OR Ostfold[tw] OR Rogaland[tw] OR "Sogn og fjordane"[tw] OR "Sogn and fjordane"[tw] OR "sogn fjordane"[tw] OR Telemark[tw] OR Troms[tw] OR Romsa[tw] OR Romssa[tw] OR "Vest Agder"[tw] OR Vestfold[tw] OR Bergen[tw] OR Stavanger[tw] OR Sandnes[tw] OR Trondheim[tw] OR Trondhjem[tw] OR Kaupangen[tw] OR Nidaros[tw] OR Drammen[tw] OR Fredrikstad[tw] OR Skien[tw] OR Tromso[tw] OR Sarpsborg[tw])) OR ("Iceland"[Mesh] OR Iceland[tw] OR Icelandic*[tw] OR islenska*[tw] OR Icelander*[tw] OR islendinga*[tw] OR Islendigar[tw] OR Inslenska[tw] OR Reykjavik[tw] OR Reykjavikurborg[tw] OR Hofudborgarsvaedi[tw] OR Sudurnes[tw] OR Vesturland[tw] OR Vestfirdir[tw] OR Westfjords[tw] OR Nordurland[tw] OR Austurland[tw] OR Sudurland[tw] OR Kopavogur[tw] OR Hafnarfjordur[tw] OR Akureyri[tw] OR Gardabaer[tw] OR Mosfellsbaer[tw] OR Keflavik[tw] OR Akranes[tw] OR Selfoss[tw] OR Seltjarnarnes[tw]))) OR (("European Union"[Mesh] OR "Europe"[Mesh:noexp] OR Europe*[tw] OR Europa*[tw] OR EU[tw] OR EEA[tw] OR "EU/EEA"[tw] OR ECSC[tw] OR Euratom[tw] OR Eurozone[tw] OR EEC[tw] OR ec[tw] OR (Schengen[tw] AND (area[tw] OR countr*[tw] OR region*[tw] OR state[tw] OR states[tw])) OR Euroregion[tw] OR Euroregions[tw] OR "Europe, Eastern"[Mesh:noexp] OR "Balkan Peninsula"[Mesh] OR Balkan[tw] OR Balkans[tw] OR "Baltic States"[Mesh] OR Baltic[tw] OR "Mediterranean Region"[Mesh] OR (Mediterranean[tw] AND (area[tw] OR countr*[tw] OR region*[tw] OR state[tw] OR states[tw])) OR (Alpine[tw] AND (area[tw] OR countr*[tw] OR region*[tw] OR state[tw] OR states[tw])) OR "Scandinavian and Nordic Countries"[Mesh] OR Scandinavia[tw] OR Scandinavian[tw] OR "Nordic country"[tw] OR "Nordic countries"[tw] OR "Nordic state"[tw] OR "Nordic states"[tw] OR Danubian[tw] OR "Iberian peninsula"[tw] OR "Peninsula iberica"[tw] OR "Peninsule Iberique"[tw] OR "Iberiar Penintsula"[tw] OR Iberia[tw] OR Anatolia[tw] OR Anadolu[tw] OR Anatole[tw] OR Anatolian[tw] OR "Yugoslavia"[Mesh] OR Yugoslavia[tw] OR "Czechoslovakia"[Mesh] OR Czechoslovakia[tw] OR "Czecho Slovakia"[tw] OR Ceskoslovensko[tw] OR "Cesko slovensko"[tw] OR Benelux[tw] OR Fennoscandia[tw] OR "Fenno Scandinavia"[tw] OR Fennoskandi*[tw] OR (Visegrad[tw] AND (Group[tw] OR Four[tw] OR Triangle[tw])) OR "Visegradska ctyrka"[tw] OR "Visegradska skupina"[tw] OR "Visegradi Egyuttmukodes"[tw] OR "Visegradi negyek"[tw] OR "Grupa Wyszehradzka"[tw] OR "Vysehradska skupina"[tw] OR "Vysehradska stvorka"[tw]) OR ("Austria"[Mesh] OR Austria*[tw] OR Osterreich*[tw] OR Oesterreich*[tw] OR Ostosterreich[tw] OR Ostoesterreich[tw] OR Sudosterreich[tw] OR Sudoesterreich[tw] OR Westosterreich[tw] OR Westoesterreich[tw] OR Burgenland[tw] OR Carinthia[tw] OR Karnten[tw] OR Kaernten[tw] OR Niederosterreich[tw] OR Niederoesterreich[tw] OR Oberosterreich[tw] OR Oberoesterreich[tw] OR Salzburg[tw] OR Saizburg[tw] OR Styria[tw] OR Steiermark[tw] OR Tyrol[tw] OR Tirol[tw] OR Vorarlberg[tw] OR Vienna[tw] OR Wien[tw] OR Graz[tw] OR Linz[tw] OR Innsbruck[tw] OR Klagenfurt[tw] OR Villach[tw] OR Wels[tw] OR "St Polten"[tw] OR "St Poelten"[tw] OR "Sankt Polten"[tw] OR "Sankt Poelten"[tw] OR Dornbirn[tw]) OR ("Belgium"[Mesh] OR Belgi*[tw] OR Belge*[tw] OR Belg[tw] OR

Brussel*[tw] OR Bruxelles[tw] OR Bruxelloise[tw] OR Walloon*[tw] OR Wallon*[tw] OR Vlaams[tw] OR Flander*[tw] OR Flandern[tw] OR Flandre[tw] OR Flemish[tw] OR Flamand[tw] OR Flemisch[tw] OR Flamisch*[tw] OR Vlaanderen[tw] OR Flamande[tw] OR Waals[tw] OR Antwerp*[tw] OR Anvers[tw] OR Henegouwen[tw] OR Hennegau[tw] OR Hainault[tw] OR Hainaut[tw] OR Liege[tw] OR Luik[tw] OR Luttich[tw] OR Limbourg[tw] OR Limburg[tw] OR Namur[tw] OR Namen[tw] OR Ostflandern[tw] OR Westflandern[tw] OR Ghent[tw] OR Gent[tw] OR Gand[tw] OR Charleroi[tw] OR Bruges[tw] OR Brugge*[tw] OR Schaerbeek[tw] OR Schaarbeek[tw] OR Anderlecht[tw] OR Leuven[tw] OR Louvain[tw]) OR ("Bulgaria"[Mesh] OR Bulgaria*[tw] OR Balgariya[tw] OR Balgarija[tw] OR Blagoevgrad*[tw] OR "Pirin Macedonia"[tw] OR Burgas[tw] OR Dobrich[tw] OR Gabrovo[tw] OR Haskovo[tw] OR Kardzhali[tw] OR Kurdzhali[tw] OR Kyustendil[tw] OR Lovech[tw] OR Lovec[tw] OR Montana[tw] OR Pazardzhik[tw] OR Pernik[tw] OR Pleven*[tw] OR Plovdiv[tw] OR Razgrad[tw] OR Rousse[tw] OR Ruse[tw] OR Rusenka[tw] OR Shumen[tw] OR Silistra[tw] OR Sliven[tw] OR Smolyan[tw] OR Sofia[tw] OR Sofyiska[tw] OR Sofiiska[tw] OR "Stara Zagora"[tw] OR Targovishte[tw] OR Varna[tw] OR "Veliko Tarnovo"[tw] OR Vidin[tw] OR Vratsa[tw] OR Vratza[tw] OR Yambol[tw]) OR ("Croatia"[Mesh] OR Croat*[tw] OR Hrvatsk*[tw] OR hrvati[tw] OR Bjelovar[tw] OR "Bjelovarsko bilogorska"[tw] OR "Brod Posavina"[tw] OR "Brodsko posavska"[tw] OR "Dubrovnik Neretva"[tw] OR "dubrovacko neretvanska"[tw] OR Zagreb[tw] OR Zagrebacka[tw] OR Istria[tw] OR Istarska[tw] OR Karlovacka[tw] OR Karlovac[tw] OR "Koprivnicko krizevacka"[tw] OR Koprivnica[tw] OR Krizevci[tw] OR "Krapina Zagorje"[tw] OR "Krapinsko zagorska"[tw] OR "Lika Senj"[tw] OR "Licko senjska"[tw] OR Medimurska[tw] OR Medimurje[tw] OR Osijek[tw] OR Baranja[tw] OR "Osjecko baranjska"[tw] OR "Pozega Slavonia"[tw] OR "Pozesko slavonska"[tw] OR "Primorje Gorski Kotar"[tw] OR "Primorsko goranska"[tw] OR "Sibensko kninska"[tw] OR "Sibensko kninske"[tw] OR Sibenik[tw] OR Knin[tw] OR Sisak[tw] OR "Sisacko moslavacka"[tw] OR Moslavina[tw] OR "Splitsko dalmatinska"[tw] OR Split[tw] OR Dalmatia[tw] OR Varazdin[tw] OR Varazdinska[tw] OR Viroviticko[tw] OR podravska[tw] OR Virovitica[tw] OR Podravina[tw] OR "Vukovarsko srijemska"[tw] OR Vukovar[tw] OR Srijem[tw] OR Zadar[tw] OR Zadarska[tw] OR Rijeka[tw] OR "Velika gorica"[tw] OR "Slavonski brod"[tw] OR Pula[tw]) OR ("Cyprus"[Mesh] OR Cyprus[tw] OR Cypriot*[tw] OR Kypros[tw] OR Kibris*[tw] OR kypriaki[tw] OR Kyprioi[tw] OR Nicosia[tw] OR Lefkosa[tw] OR Lefkosia[tw] OR Famagusta[tw] OR Magusa[tw] OR Ammochostos[tw] OR Gazimagusa[tw] OR Kyrenia[tw] OR Girne[tw] OR Keryneia[tw] OR Larnaca[tw] OR Larnaka[tw] OR Iskele[tw] OR Limassoll[tw] OR Lemesos[tw] OR Limasol[tw] OR Leymosun[tw] OR Paphos[tw] OR Pafos[tw] OR Baf[tw] OR Strovolos[tw] OR Lakatamia[tw] OR Lakadamya[tw] OR "Kato Polemidia"[tw] OR "Kato Polemidhia"[tw] OR Aglandjia[tw] OR Eglence[tw] OR Aglantzia[tw] OR Aradhippou[tw] OR Aradippou[tw] OR Engomi[tw]) OR ("Czech Republic"[Mesh] OR Czech*[tw] OR Cesky[tw] OR Ceska[tw] OR Cech[tw] OR Cestina[tw] OR Prague[tw] OR Praha[tw] OR Prag[tw] OR Stredoces*[tw] OR Jihoces*[tw] OR Bohemia[tw] OR Bohemian[tw] OR Plzen*[tw] OR Pilsen[tw] OR Karlovars*[tw] OR "Karlovy Vary"[tw] OR Usteck*[tw] OR Usti[tw] OR Liberec*[tw] OR "Hradec Kralove"[tw] OR Kralovehradec*[tw] OR Pardubic*[tw] OR Olomouc*[tw] OR Olomoc[tw] OR Holomoc[tw] OR Moravskoslezs*[tw] OR Jihomorav*[tw] OR Moravia[tw] OR Moravian[tw] OR Morava[tw] OR Vysocina[tw] OR Zlin[tw] OR Zlinsk*[tw] OR "Ceske Budejovice"[tw] OR Budweis[tw] OR Brno[tw] OR Ostrava[tw]) OR ("Denmark"[Mesh] OR Denmark[tw] OR Danish*[tw] OR dane[tw] OR danes[tw] OR Danmark[tw] OR dansk*[tw] OR Hovedstaden[tw] OR Midtjylland[tw] OR Nordjylland[tw] OR Sjaelland[tw] OR Sealand[tw] OR "Zealand region"[tw] OR "region Zealand"[tw] OR Syddanmark[tw] OR Jutland[tw] OR Jylland[tw] OR Sonderjyllands[tw] OR Copenhagen[tw] OR Kobenhavn[tw] OR Arhus[tw] OR Aarhus[tw] OR Bornholm[tw] OR Frederiksberg[tw] OR Frederiksborg[tw] OR Ringkjobing[tw] OR Viborg[tw] OR Vejle[tw] OR Roskilde[tw] OR Storstrom[tw] OR Vestsjaellands[tw] OR "West Zealand"[tw] OR Funen[tw] OR Ribe[tw] OR "Kalaallit Nunaat"[tw] OR Gronland[tw] OR Foroyar[tw] OR Faeroerne[tw] OR "Faroe islands"[tw] OR Aalborg[tw] OR Alborg[tw] OR Odense[tw] OR Esbjerg[tw] OR Gentofte[tw] OR Gladsaxe[tw] OR Randers[tw] OR Kolding[tw]) OR ("Estonia"[Mesh] OR Estonia*[tw] OR Eesti[tw] OR Eestlased[tw] OR Eestlane[tw] OR Harju[tw] OR Harjumaa[tw] OR Hiiu[tw] OR Hiiumaa[tw] OR "Ida Viru"[tw] OR "Ida Virumaa"[tw] OR Jarvamaa[tw] OR Jarva[tw] OR Jogevamaa[tw] OR Jogeva[tw] OR Laanemaa[tw] OR Laane[tw] OR "Laane Virumaa"[tw] OR Parnu[tw] OR Parnumaa[tw] OR Polva[tw] OR Polvamaa[tw] OR Rapla[tw] OR Raplamaa[tw] OR Saare[tw] OR Saaremaa[tw] OR Tartu[tw] OR Tartumaa[tw] OR Valga[tw] OR Valgamaa[tw] OR Viljandimaa[tw] OR Viljandi[tw] OR Voru[tw] OR Vorumaa[tw] OR Tallinn[tw] OR Narva[tw] OR "Kohtla Jarve"[tw] OR Rakvere[tw] OR Maardu[tw] OR Sillamae[tw] OR Kuressaare[tw]) OR ("Finland"[Mesh] OR Finland[tw] OR Finnish*[tw] OR Finn[tw] OR Finns[tw] OR Suomi[tw] AND Suomen[tw] OR Suomalaiset[tw] OR Aland[tw] OR Ahvenanmaa[tw] OR Uusimaa[tw] OR Nyland[tw] OR Karelia[tw] OR Karjala[tw] OR Karelen[tw] OR Ostrobothnia[tw] OR Pohjanmaa[tw] OR Osterbotten[tw] OR Savonia[tw] OR Savo[tw] OR Savolax[tw] OR Kainuu[tw] OR Kajanaland*[tw] OR "Kanta Hame"[tw] OR Tavastia[tw] OR Tavastland[tw] OR Kymenlaakso[tw] OR Kymmenedalen[tw] OR Lapland[tw] OR Lappi[tw] OR Lappland[tw] OR "Paijat Hame"[tw] OR Pirkanmaa[tw] OR Birkaland[tw] OR Satakunta[tw] OR Satakunda[tw] OR Helsinki[tw] OR Helsingfors[tw] OR Espoo[tw] OR Esbo[tw] OR Tampere[tw] OR Tammerfors[tw] OR Vantaa[tw] OR Vanda[tw] OR Oulu[tw] OR Uleaborg[tw] OR Turku[tw] OR Abo[tw] OR Jyvaskyla[tw] OR Kuopio[tw] OR Lahti[tw] OR Lahtis[tw] OR Kouvola[tw]) OR ("France"[Mesh] OR France[tw] OR French*[tw] OR Francais*[tw] OR Alsace[tw] OR Elsass[tw] OR Aquitaine[tw] OR Aquitania[tw] OR Akitania[tw] OR Aguiene[tw] OR Auvergne[tw] OR Auvernhe[tw] OR Auvernha[tw] OR Normandie[tw] OR Normandy[tw] OR Normaundie[tw] OR Bourgogne[tw] OR Burgundy[tw] OR Bregogne[tw] OR Borgoegne[tw] OR Borgogne[tw] OR Brittany[tw] OR Breizh[tw] OR Bertaeyn[tw] OR Bretagne[tw] OR "Champagne Ardenne"[tw] OR Corse[tw] OR Corsica[tw] OR "Franche Comte"[tw] OR "Frantche Comte"[tw] OR "Franche Comtat"[tw] OR Guadeloupe[tw] OR Guyane[tw] OR Guiana[tw] OR "Languedoc Roussillon"[tw] OR "Lengadoc Rosselhon"[tw] OR "Llenguadoc-Rossello"[tw] OR Limousin[tw] OR Lemosin[tw] OR Lorraine[tw] OR Lothringen[tw] OR Lottringe[tw] OR Martinique[tw] OR "Midi Pyrenees"[tw] OR "Miegjorn Pireneus"[tw] OR "Mieidia Pireneus"[tw] OR "Mediodia Pirineos"[tw] OR "Pays de la Loire"[tw] OR "Broiou al Liger"[tw] OR Picardie[tw] OR Picardy[tw] OR "Poitou Charentes"[tw] OR "Peitau Charantas"[tw] OR "Poetou-Cherentes"[tw] OR Provence[tw] OR Provenca[tw] OR Prouvenco[tw] OR "Cote d Azur"[tw] OR "Cote d'Azur"[tw] OR "Costo d'Azur"[tw] OR "Costo d Azur"[tw] OR "Costa d'Azur"[tw] OR "Costa d Azur"[tw] OR Reunion[tw] OR "Rhone Alpes"[tw] OR "Rono Arpes"[tw] OR "Rose Aups"[tw] OR Ain[tw] OR Aisne[tw] OR Allier[tw] OR "Alpes de Haute Provence"[tw] OR "Haute Alpes"[tw] OR "Alpes Maritimes"[tw] OR Ardeche[tw] OR Ardennes[tw] OR Ariege[tw] OR Aube[tw] OR Aude[tw] OR Aveyron[tw] OR "Bas Rhin"[tw] OR "Bouches du Rhone"[tw] OR Calvados[tw] OR Cantal[tw] OR Charente[tw] OR Cher[tw] OR Correze[tw] OR "Corse du Sud"[tw] OR "Cote d Or"[tw] OR "Cote d'Or"[tw] OR "Cotes d Armor"[tw] OR "Cotes d'Armor"[tw] OR Creuse[tw] OR "Deux Sevres"[tw] OR Dordogne[tw] OR Doubs[tw] OR Drome[tw] OR Essonne[tw] OR Eure[tw] OR Finistere[tw] OR Gard[tw] OR Gers[tw] OR Gironde[tw] OR "Haute Corse"[tw] OR "Haute Garonne"[tw] OR "Haute Marne"[tw] OR "Hautes Alpes"[tw] OR "Haute Saone"[tw] OR "Haute Savoie"[tw] OR "Hautes Pyrenees"[tw] OR "Haute Vienne"[tw] OR "Haut Rhin"[tw] OR "Hauts de Seine"[tw] OR Herault[tw] OR "Ile de France"[tw] OR "Ille et Vilaine"[tw] OR Indre[tw] OR Isere[tw] OR Jura[tw] OR Landes[tw] OR Loire[tw] OR Loiret[tw] OR (Lot[tw] AND (departement[tw] OR department[tw])) OR "Lot et Garonne"[tw] OR "Loir et Cher"[tw] OR Lozere[tw] OR Manche[tw] OR Marne[tw] OR Mayenne[tw] OR Mayotte[tw] OR "Meurthe et Moselle"[tw] OR Meuse[tw] OR Morbihan[tw] OR Moselle[tw] OR (Nord[tw] AND (department[tw] OR departement[tw])) OR Nievre[tw] OR Oise[tw] OR Orne[tw] OR "Pas de calais"[tw] OR "Noord-Nauw van Kales"[tw] OR Paris[tw] OR "Puy de dome"[tw] OR "Pyrenees Atlantiques"[tw] OR "Pyrenees Orientales"[tw] OR Rhone[tw] OR Sarthe[tw] OR Savoie[tw] OR "Seine et Marne"[tw] OR "Seine Maritime"[tw] OR Somme[tw] OR Tarn[tw] OR "Territoire de Belfort"[tw] OR "Val de Marne"[tw] OR "Val d Oise"[tw] OR Var[tw] OR Vaucluse[tw] OR Vendee[tw] OR Vienne[tw] OR Vosges[tw] OR Yonne[tw] OR Yvelines[tw] OR Marseille[tw] OR Lyon[tw] OR Nice[tw] OR Nantes[tw] OR Strasbourg[tw] OR Montpellier[tw] OR Bordeaux[tw] OR Lille[tw] OR Toulouse[tw] OR "Outre Mer"[tw] OR "Seine Saint Denis"[tw]) OR ("Germany"[Mesh] OR German*[tw] OR Deutsch*[tw] OR Bundesrepublik[tw] OR Westdeutschland[tw] OR Ostdeutschland[tw] OR Baden[tw] OR Wuerttemberg[tw] OR Wurttemberg[tw] OR Bayern[tw] OR Bavaria[tw] OR Berlin[tw] OR Brandenburg[tw] OR Bremen[tw] OR Oldenburg[tw] OR Mitteldeutschland[tw] OR Rhein[tw] OR Rhine[tw] OR Hannover[tw] OR Braunschweig[tw] OR Gottingen[tw] OR Goettingen[tw] OR Nurnberg[tw] OR Nuernberg[tw] OR Ruhr[tw] OR Koln[tw] OR koeln[tw] OR Bonn[tw] OR Hamburg[tw] OR Hessen[tw] OR Hesse[tw] OR Hessia[tw] OR Mecklenburg[tw] OR Vorpommern[tw] OR Pomerania[tw] OR Niedersachsen[tw] OR Neddersassen[tw] OR Saxony[tw] OR Niederbayern[tw] OR "Northern Rhine"[tw] OR "North Rhine"[tw] OR Westphalia[tw] OR Westfalen[tw] OR "Rhineland Palatinate"[tw] OR "Rheinland Pfalz"[tw] OR Saarland[tw] OR Sachsen[tw] OR "Schleswig Holstein"[tw] OR Thuringia[tw] OR Thuringen[tw] OR Thueringen[tw] OR Munchen[tw] OR Muenchen[tw] OR Munich[tw] OR Frankfurt[tw] OR Stuttgart[tw] OR Dusseldorf[tw] OR Duesseldorf[tw] OR Dortmund[tw] OR Essen[tw]) OR ("Greece"[Mesh] OR Greece[tw] OR "Hellenic republic"[tw] OR Greek*[tw] OR Ellada[tw] OR Elladas[tw] OR "Elliniki Dimokratia"[tw] OR Hellas[tw] OR Hellenes[tw] OR Attica[tw] OR Attiki[tw] OR Makedonia*[tw] OR Macedonia[tw] OR Thraki[tw] OR Thrace[tw] OR Crete[tw] OR Kriti[tw] OR "Ionia Nisia"[tw] OR "Ionion neson"[tw] OR "Ionion nIson"[tw] OR "Ionian islands"[tw] OR "Ionian island"[tw] OR Epirus[tw] OR Ipeiros[tw] OR "Perifereia Ipeirou"[tw] OR "North aegean"[tw] OR "Northern Aegean"[tw] OR "Aegean islands"[tw] OR "Aegean island"[tw] OR "Nisoi Agaiou"[tw] OR "Notio Aigaio"[tw] OR Peloponnese[tw] OR Peloponniso*[tw] OR Thessaly[tw] OR Thessalia[tw] OR Thessalian[tw] OR Petthalia[tw] OR "Voreio Aigaio"[tw] OR "Voreio Aigaiou"[tw] OR "South aegean"[tw] OR "Southern Aegean"[tw] OR "Mount athos"[tw] OR "Oros Athos"[tw] OR Cyclades[tw] OR Cycklades[tw] OR Kiklades[tw] OR Dodecanese[tw] OR Dodekanisa[tw] OR Athens[tw] OR Athina[tw] OR Thessaloniki[tw] OR Thessalonica[tw] OR Patras[tw] OR Patra[tw] OR Pireas[tw] OR Piraeus[tw] OR Larissa[tw] OR Larisa[tw] OR Heraklion[tw] OR Heraclion[tw] OR Iraklion[tw] OR Irakleion[tw] OR Iraklio[tw] OR Volos[tw] OR Rhodes[tw] OR Rodos[tw] OR Ioannina[tw] OR Janina[tw] OR Yannena[tw] OR Chania[tw] OR Chalcis[tw] OR Chalkida[tw]) OR ("Hungary"[Mesh] OR Hungar*[tw] OR Magyarorszag[tw] OR Magyar*[tw] OR Dunantuli[tw] OR Transdanubia[tw] OR Dunantul[tw] OR "Great Plain"[tw] OR "Eszak Alfold"[tw] OR "Del Alfold"[tw] OR "Alfold es eszak"[tw] OR "Northern Alfold"[tw] OR "North Alfold"[tw] OR "South Alfold"[tw] OR "Southern Alfold"[tw] OR Bacs[tw] OR Kiskun[tw] OR Baranya[tw] OR Bekes[tw] OR Borsod[tw] OR Abauj[tw] OR Zemplen[tw] OR Budapest[tw] OR Csongrad[tw] OR Fejer[tw] OR gyor[tw] OR moson[tw] OR sopron[tw] OR hajdu[tw] OR bihar[tw] OR

Heves[tw] OR "jasz nagykun szolnok"[tw] OR komarom[tw] OR esztergom[tw] OR Nograd[tw] OR (Pest[tw] AND (megye[tw] OR county[tw])) OR Somogy[tw] OR szabolcs[tw] OR szatmar[tw] OR bereg[tw] OR Tolna[tw] OR Vas[tw] OR Veszprem[tw] OR Zala[tw] OR Debrecen[tw] OR Miskolc[tw] OR Szeged[tw] OR Pecs[tw] OR Gyor[tw] OR Nyiregyhaza[tw] OR Kecskemet[tw] OR Szekesfehervar[tw] OR Szombathely[tw]) OR ("Ireland"[Mesh] OR Ireland[tw] OR Eire[tw] OR Irish*[tw] OR Fingal[tw] OR "Fine Gall"[tw] OR Dublin[tw] OR "Ath Cliath"[tw] OR "Dun Laoghaire"[tw] OR Wicklow[tw] OR "Cill Mhantain"[tw] OR "Chill Mhantain"[tw] OR Wexford[tw] OR "Loch Garman"[tw] OR Carlow[tw] OR Ceatharlach[tw] OR Kildare[tw] OR "Cill Dara"[tw] OR "Chill Dara"[tw] OR Meath[tw] OR "An Mhi"[tw] OR "Contae na Mi"[tw] OR Louth[tw] OR "Contae Lu"[tw] OR Monaghan[tw] OR Muineachan[tw] OR Mhuineachain[tw] OR Cavan[tw] OR "An Cabhan"[tw] OR "An Cabhain"[tw] OR Longford[tw] OR "An Longfort"[tw] OR "an Longfoirt"[tw] OR Langfurd[tw] OR Westmeath[tw] OR "An Iarmhi"[tw] OR "na Iarmhi"[tw] OR Offaly[tw] OR "Uibh Fhaili"[tw] OR Laois[tw] OR Laoise[tw] OR Kilkenny[tw] OR "Chill Chainnigh"[tw] OR "Cill Chainnigh"[tw] OR Waterford[tw] OR "Port Lairge"[tw] OR Watterford[tw] OR Cork[tw] OR Corcaigh[tw] OR Chorcai[tw] OR Kerry[tw] OR Ciarrai[tw] OR Chiarrai[tw] OR Limerick[tw] OR Luimneach[tw] OR Luimnigh[tw] OR Tipperary[tw] OR "Tiobraid Arann"[tw] OR "Thiobraid Arann"[tw] OR Clare[tw] OR "An Clar"[tw] OR "an Chlair"[tw] OR Galway[tw] OR Gaillimh[tw] OR "na Gaillimhe"[tw] OR Mayo[tw] OR "Maigh Eo"[tw] OR "Mhaigh Eo"[tw] OR Roscommon[tw] OR "Ros comain"[tw] OR Sligo[tw] OR Sligeach[tw] OR Shligigh[tw] OR Leitrim[tw] OR Liatroim[tw] OR Liatroma[tw] OR Donegal[tw] OR "Dhun na nGall"[tw] OR Dinnygal[tw] OR Dunnyga[tw] OR Leinster[tw] OR Laighin[tw] OR "Cuige Laighean"[tw] OR Munster[tw] OR Mumhain[tw] OR "Cuige Mumhan"[tw] OR Connacht[tw] OR Connachta[tw] OR Drogheda[tw] OR "Droichead Atha"[tw] OR Dundalk[tw] OR "Dun Dealgan"[tw] OR Swords[tw] OR Sord[tw] OR Bray[tw] OR Bre[tw] OR Navan[tw] OR "An Uaimh"[tw]) OR ("Italy"[Mesh] OR Italy[tw] OR Italia*[tw] OR Abruzzo[tw] OR Abruzzi[tw] OR Basilicata[tw] OR Lucania[tw] OR Calabria[tw] OR Campania[tw] OR "Emilia Romagna"[tw] OR "friuli venezia giulia"[tw] OR Lazio[tw] OR Latium[tw] OR Liguria*[tw] OR Lombardy[tw] OR Lombardia[tw] OR Marche[tw] OR Marches[tw] OR Molisano[tw] OR Molise[tw] OR Piedmont*[tw] OR Piemonte[tw] OR Bolzano[tw] OR Bozen[tw] OR Trentino[tw] OR Trento[tw] OR Puglia[tw] OR Apulia[tw] OR Sardinia[tw] OR Sardegna[tw] OR Sicily[tw] OR Sicilia[tw] OR Toscana[tw] OR Tuscany[tw] OR Umbria[tw] OR "Valle d Aosta"[tw] OR "Vallee d Aoste"[tw] OR "Valle d'Aosta"[tw] OR "Vallee d'Aoste"[tw] OR "Aosta Valley"[tw] OR Veneto[tw] OR Venetia[tw] OR Triveneto[tw] OR Rome[tw] OR Roma[tw] OR Milan[tw] OR Milano[tw] OR Naples[tw] OR Napoli[tw] OR Turin[tw] OR Torino[tw] OR Palermo[tw] OR Genoa[tw] OR Genova[tw] OR Bologna[tw] OR Florence[tw] OR Firenze[tw] OR Bari[tw] OR Catania[tw]) OR ("Latvia"[Mesh] OR Latvi*[tw] OR Riga[tw] OR Courland[tw] OR Kurzeme[tw] OR Kurland[tw] OR Latgale[tw] OR Lettgallia[tw] OR Latgola[tw] OR Latgalia[tw] OR Vidzeme[tw] OR Vidumo[tw] OR Semigallia[tw] OR Semigalia[tw] OR Zemgale[tw] OR Pieriga[tw] OR Daugavpils[tw] OR Dinaburg[tw] OR Jekabpils[tw] OR Jakobstadt[tw] OR Jelgava[tw] OR Jurmala[tw] OR Liepaja[tw] OR Libau[tw] OR Rezekne[tw] OR Rezne[tw] OR Rositten[tw] OR Valmiera[tw] OR Wolmar[tw] OR Ventspils[tw] OR Windau[tw] OR Ogre[tw]) OR ("Lithuania"[Mesh] OR Lithuania*[tw] OR "Lietuvos Respublika"[tw] OR Lietuva[tw] OR lietuviu[tw] OR Alytus[tw] OR Alytaus[tw] OR Kaunas[tw] OR Kauno[tw] OR Klaipeda[tw] OR Klaipedos[tw] OR Marijampoles[tw] OR Marijampole[tw] OR Panevezys[tw] OR Panevezio[tw] OR Siauliai[tw] OR Siauliu[tw] OR Taurages[tw] OR Taurage[tw] OR Telsiu[tw] OR Telsiai[tw] OR Utenos[tw] OR Utena[tw] OR Vilnius[tw] OR Vilniaus[tw] OR Mazeikiai[tw] OR Jonava[tw] OR Mazeikiu[tw] OR Jonavos[tw]) OR ("Luxembourg"[Mesh] OR Luxembourg*[tw] OR Luxemburg[tw] OR Letzebuerg[tw] OR Diekirch[tw] OR Grevenmacher[tw] OR "Esch sur Alzette"[tw] OR "Esch Uelzecht"[tw] OR "Esch an der Alzette"[tw] OR "Esch an der Alzig"[tw] OR Dudelange[tw] OR Diddeleng[tw] OR Dudelingen[tw] OR Duedelingen[tw] OR Schifflange[tw] OR Scheffleng[tw] OR Schifflingen[tw] OR Bettembourg[tw] OR Beetebuerg[tw] OR Bettemburg[tw] OR Petange[tw] OR Peiteng[tw] OR Petingen[tw] OR Ettelbruck[tw] OR Ettelbreck[tw] OR Ettelbrueck[tw] OR Diekirch[tw] OR Dikrech[tw] OR Strassen[tw] OR Stroossen[tw] OR Bertrange[tw] OR Bartreng[tw] OR Bartringen[tw]) OR ("Malta"[Mesh] OR Malta[tw] OR Maltese*[tw] OR Maltin[tw] OR Gozo[tw] OR Ghawdex[tw] OR Valletta[tw] OR "Il Belt"[tw] OR Birkirkara[tw] OR "B Kara"[tw] OR "B'Kara"[tw] OR Birchircara[tw] OR Mosta[tw] OR Qormi[tw] OR "St Paul s Bay"[tw] OR "St Paul's Bay"[tw] OR "Pawl il Bahar"[tw] OR Zabbar[tw] OR Sliema[tw] OR Naxxar[tw] OR Gwann[tw] OR "St John"[tw] OR Zebbug[tw] OR "Citta rohan"[tw] OR Fgura[tw]) OR ("Netherlands"[Mesh] OR Netherlands[tw] OR Nederland*[tw] OR Dutch*[tw] OR Drenthe[tw] OR Flevoland[tw] OR Friesland[tw] OR Fryslan[tw] OR Frisia[tw] OR Gelderland[tw] OR Guelders[tw] OR Groningen[tw] OR Limburg[tw] OR Brabant[tw] OR Holland[tw] OR Overijssel[tw] OR Overissel[tw] OR Utrecht[tw] OR Zeeland[tw] OR Amsterdam[tw] OR Rotterdam[tw] OR Hague[tw] OR "s-Gravenhage"[tw] OR "Den Haag"[tw] OR Eindhoven[tw] OR Tilburg[tw] OR Almere[tw] OR Breda[tw] OR Nijmegen[tw] OR Nimeguen[tw]) OR ("Poland"[Mesh] OR Poland[tw] OR Polska[tw] OR Polish[tw] OR Pole[tw] OR Poles[tw] OR Polski[tw] OR Polak[tw] OR Polka[tw] OR Polacy[tw] OR Dolnoslaskie[tw] OR Silesia*[tw] OR Slask[tw] OR Pomorskie[tw] OR Pomerania*[tw] OR Kujawsko[tw] OR Kuyavian[tw] OR Lodzkie[tw] OR Lodz[tw] OR Lubelskie[tw] OR Lublin[tw] OR Lubuskie[tw] OR Lubusz[tw] OR Lubus[tw] OR Malopolskie[tw] OR Mazowieckie[tw] OR Mazowske[tw] OR Masovia[tw] OR Masovian[tw] OR Opolskie[tw] OR Opole[tw] OR Podkarpackie[tw] OR Subcarpathian*[tw] OR Podlaskie[tw] OR Podlachia[tw] OR Podlasie[tw] OR Slaskie[tw] OR Swietokrzyskie[tw] OR "Varmia Mazuria"[tw] OR "Varmian Mazurian"[tw] OR "Varmia Masuria"[tw] OR "Varmian Masurian"[tw] OR "Warmia Mazury"[tw] OR "Warminsko Mazurskie"[tw] OR "Warmian Masurian"[tw] OR Wielkopolskie[tw] OR Zachodniopomorskie[tw] OR Warsaw[tw] OR Warszawa[tw] OR Krakow[tw] OR Cracow[tw] OR Wroclaw[tw] OR Poznan[tw] OR Gdansk[tw] OR Szczecin[tw] OR Bydgoszcz[tw] OR Katowice[tw]) OR ("Portugal"[Mesh] OR Portugal[tw] OR Portugues*[tw] OR Azores[tw] OR Acores[tw] OR Madeira[tw] OR Alentejo[tw] OR Algarve[tw] OR Lisboa[tw] OR Lisbon[tw] OR "Alto Tras-os-Montes"[tw] OR (Ave[tw] AND (community[tw] OR intermunicipal[tw] OR comunidade[tw])) OR Mondego[tw] OR Vouga[tw] OR Beira[tw] OR Cavado[tw] OR Lafoes[tw] OR Douro[tw] OR Porto[tw] OR Oporto[tw] OR Tejo[tw] OR Minho[tw] OR Setubal[tw] OR Pinhal[tw] OR "Serra da Estrela"[tw] OR Tamega[tw] OR Leira[tw] OR Santarem[tw] OR Beja[tw] OR Faro[tw] OR Evora[tw] OR Portalegre[tw] OR "Castelo Branco"[tw] OR Guarda[tw] OR Cimbra[tw] OR Aveiro[tw] OR Viseu[tw] OR Braganca[tw] OR Braganza[tw] OR Braga[tw] OR "Vila real"[tw] OR "Viana do Castelo"[tw] OR Gaia[tw] OR Amadora[tw] OR Funchal[tw] OR Coimbra[tw] OR Almada[tw] OR (Agualva[tw] AND Cacem[tw])) OR ("Romania"[Mesh] OR Romania*[tw] OR Rumania*[tw] OR Roumania*[tw] OR Romani[tw] OR Rumani[tw] OR Alba[tw] OR Arad[tw] OR Arges[tw] OR Bacau[tw] OR Bihor[tw] OR "Bistrita Nasaud"[tw] OR Botosani[tw] OR Braila[tw] OR Brasov[tw] OR Kronstadt[tw] OR Brasso[tw] OR Brassovia[tw] OR Coron[tw] OR Bucharest[tw] OR Bucuresti[tw] OR Buzau[tw] OR Calarasi[tw] OR "Caras-Severin"[tw] OR Cluj[tw] OR Klausenburg[tw] OR Kolozsvar[tw] OR Constanta[tw] OR Tomis[tw] OR Konstantia[tw] OR Kostence[tw] OR Covasna[tw] OR Dambovita[tw] OR Dolj[tw] OR Galati[tw] OR Galatz[tw] OR Galac[tw] OR Kalas[tw] OR Giurgiu[tw] OR Gorj[tw] OR Harghita[tw] OR Hunedoara[tw] OR Ialomita[tw] OR Iasi[tw] OR Jassy[tw] OR Lassy[tw] OR Ilfov[tw] OR Maramures[tw] OR Mehedinti[tw] OR Mures[tw] OR Neamt[tw] OR (Olt[tw] AND (river[tw] OR county[tw] OR region[tw] OR judetul[tw] OR Raul[tw])) OR Prahova[tw] OR Salaj[tw] OR "Satu Mare"[tw] OR Sibiu[tw] OR Suceava[tw] OR Teleorman[tw] OR Timis[tw] OR Tulcea[tw] OR Valcea[tw] OR Vilcea[tw] OR Vaslui[tw] OR Vrancea[tw] OR Timisoara[tw] OR Temeswar[tw] OR Temeschburg[tw] OR Temeschwar[tw] OR Temesvar[tw] OR Temisvar[tw] OR Timisvar[tw] OR Temesva[tw] OR Craiova[tw] OR Ploiesti[tw] OR Ploesti[tw] OR Oradea[tw] OR Varad[tw] OR Varat[tw]) OR ("Slovakia"[Mesh] OR Slovakia[tw] OR Slovensk*[tw] OR Slovak*[tw] OR Slovaci[tw] OR Slovenki[tw] OR Bratislav*[tw] OR Presporok[tw] OR Pressburg[tw] OR Pressburg[tw] OR Posonium[tw] OR Banskobystri*[tw] OR "Banska Bystrica"[tw] OR Neusohl[tw] OR Besztercebanya[tw] OR Kosic*[tw] OR Kaschau[tw] OR Kassa[tw] OR Nitrian*[tw] OR Nitra[tw] OR Neutra[tw] OR Nyitra[tw] OR Nyitria[tw] OR Trnav*[tw] OR Tyrnau[tw] OR Nagyszombat[tw] OR Tyrnavia[tw] OR Presov*[tw] OR Trencian*[tw] OR Trencin[tw] OR Trentschin[tw] OR Trencsen[tw] OR Zilina[tw] OR Sillein[tw] OR Zsolna[tw] OR Zylina[tw] OR (Martin[tw] AND (city[tw] OR Svaty[tw])) OR Turocszentmarton[tw] OR Poprad[tw] OR Deutschendorf[tw] OR Zvolen[tw]) OR ("Slovenia"[Mesh] OR Slovenia*[tw] OR Slovenija[tw] OR slovensk*[tw] OR Slovenci[tw] OR Slovene*[tw] OR Gorenjska[tw] OR Carniola[tw] OR Goriska[tw] OR Gorizia[tw] OR Jugovzhodna[tw] OR Koroska[tw] OR Carinthia[tw] OR "Notranjsko kraska"[tw] OR "Obalno kraska"[tw] OR "Coastal karst"[tw] OR Osrednjeslovenska[tw] OR Podravska[tw] OR Drava[tw] OR Pomurska[tw] OR Mura[tw] OR Savinjska[tw] OR Savinja[tw] OR Spodnjeposavska[tw] OR Zasavska[tw] OR "Central Sava"[tw] OR Posavska[tw] OR "Lower Sava"[tw] OR Ljubljana[tw] OR Laibach[tw] OR Lubiana[tw] OR Maribor[tw] OR "Marburg an der Drau"[tw] OR Kranj[tw] OR Carnium[tw] OR Creina[tw] OR Chreina[tw] OR Krainbur[tw] OR Koper[tw] OR Capodistria[tw] OR Kopar[tw] OR Celje[tw] OR "Novo mesto"[tw] OR Neustadtl[tw] OR Domzale[tw] OR Velenje[tw] OR Wollan[tw] OR Woellan[tw] OR "Nova Gorica"[tw] OR Kamnik[tw]) OR ("Spain"[Mesh] OR Spain[tw] OR Espana[tw] OR Spanish[tw] OR Espanol*[tw] OR Spaniard*[tw] OR Andalucia[tw] OR Andalusia[tw] OR Aragon[tw] OR Arago[tw] OR Cantabria[tw] OR Canarias[tw] OR "Canary Islands"[tw] OR (Canaries[tw] AND island*[tw]) OR "Castile and leon"[tw] OR "Castilla y Leon"[tw] OR "Castile La Mancha"[tw] OR "Castilla La Mancha"[tw] OR Cataluna[tw] OR Catalonia[tw] OR Ceuta[tw] OR Madrid[tw] OR Melilla[tw] OR Navarra[tw] OR Navarre[tw] OR Valencia*[tw] OR Extremadura[tw] OR Galicia[tw] OR Balears[tw] OR "Balearic Islands"[tw] OR "Balear Islands"[tw] OR Baleares[tw] OR "La Rioja"[tw] OR "Pais Vasco"[tw] OR "Basque Country"[tw] OR "Baske region"[tw] OR Euskadi[tw] OR Asturias[tw] OR Murcia[tw] OR Coruna[tw] OR Alava[tw] OR Araba[tw] OR Albacete[tw] OR Alicante[tw] OR Alacant[tw] OR Almeria[tw] OR Avila[tw] OR Badajoz[tw] OR Badajos[tw] OR Barcelona[tw] OR Burgos[tw] OR Caceres[tw] OR Cadiz[tw] OR Castellon[tw] OR Castello[tw] OR "Ciudad Real"[tw] OR Cordoba[tw] OR Cuenca[tw] OR Eivissa[tw] OR Ibiza[tw] OR Formentera[tw] OR "El Hierro"[tw] OR Fuerteventura[tw] OR Galiza[tw] OR Girona[tw] OR Gerona[tw] OR "Gran Canaria"[tw] OR Granada[tw] OR Guadalajara[tw] OR Guipuzcoa[tw] OR Gipuzkoa[tw] OR Huelva[tw] OR Huesca[tw] OR Jaen[tw] OR "La Gomera"[tw] OR "La Palma"[tw] OR Lanzarote[tw] OR Leon[tw] OR Lleida[tw] OR Lerida[tw] OR Lugo[tw] OR Malaga[tw] OR Mallorca[tw] OR Majorca[tw] OR Menorca[tw] OR Minorca[tw] OR Murcia[tw] OR Ourense[tw] OR Orense[tw] OR Palencia[tw] OR Pontevedra[tw] OR Salamanca[tw] OR Segovia[tw] OR Sevilla[tw] OR Seville[tw] OR Soria[tw] OR Tarragona[tw] OR Tenerife[tw] OR Teruel[tw] OR Toledo[tw] OR Valladolid[tw] OR Vizcaya[tw] OR Biscay[tw] OR Zamora[tw] OR Zaragoza[tw] OR Saragossa[tw] OR "Las Palmas"[tw] OR Bilbao[tw] OR Bilbo[tw]) OR ("Sweden"[Mesh] OR Sweden[tw] OR Sverige[tw] OR Swedish[tw] OR Svenska[tw] OR svenskar[tw] OR Swede[tw] OR

Swedes[tw] OR Norrland[tw] OR Mellansverige[tw] OR Smaland[tw] OR Stockholm*[tw] OR Sydsverige[tw] OR Vastsverige[tw] OR Blekinge[tw] OR Dalarna[tw] OR Gavleborg*[tw] OR Gotland*[tw] OR Halland*[tw] OR Jamtland*[tw] OR Jonkoping*[tw] OR Kalmar[tw] OR Kronoberg*[tw] OR Norrbotten*[tw] OR Orebro[tw] OR Ostergotland*[tw] OR Skane[tw] OR Sodermanlands[tw] OR Uppsala[tw] OR Varmland*[tw] OR Vasterbotten*[tw] OR Vasternorrland*[tw] OR Vastmanland*[tw] OR vastergotland*[tw] OR Gotaland*[tw] OR Gothenburg[tw] OR Goteborg[tw] OR Malmo[tw] OR Vasteras[tw] OR Linkoping[tw] OR Helsingborg[tw] OR Halsingborg[tw] OR Norrkoping[tw]) OR ("Great Britain"[Mesh] OR GB[tw] OR "United kingdom"[tw] OR UK[tw] OR Britain[tw] OR British[tw] OR England[tw] OR English[tw] OR Scotland[tw] OR Scottish[tw] OR Scots[tw] OR Wales[tw] OR Cymru[tw] OR Welsh[tw] OR Irish[tw] OR Avon[tw] OR Bedfordshire[tw] OR Berkshire[tw] OR Bristol[tw] OR Buckinghamshire[tw] OR Cambridgeshire[tw] OR "Isle of Ely"[tw] OR Cheshire[tw] OR Cleveland[tw] OR Cornwall[tw] OR Cumberland[tw] OR Cumbria[tw] OR Derbyshire[tw] OR Devon[tw] OR Dorset[tw] OR Durham[tw] OR Essex[tw] OR Gloucestershire[tw] OR Hampshire[tw] OR Southampton[tw] OR (Hereford[tw] AND Worcester[tw]) OR Hertfordshire[tw] OR Herefordshire[tw] OR Humberside[tw] OR Huntingdon[tw] OR Huntingdonshire[tw] OR "Isle of Wight"[tw] OR Kent[tw] OR Lancashire[tw] OR Leicestershire[tw] OR Lincolnshire[tw] OR London[tw] OR Manchester[tw] OR Merseyside[tw] OR Middlesex[tw] OR Norfolk[tw] OR Northamptonshire[tw] OR Northumberland[tw] OR Nottinghamshire[tw] OR Oxfordshire[tw] OR Peterborough[tw] OR Rutland[tw] OR Shropshire[tw] OR Salop[tw] OR Somerset[tw] OR Yorkshire[tw] OR Staffordshire[tw] OR Suffolk[tw] OR Surrey[tw] OR Sussex[tw] OR (Tyne[tw] AND Wear[tw]) OR Warwickshire[tw] OR Midlands[tw] OR Westmorland[tw] OR Wiltshire[tw] OR Worcestershire[tw] OR "Isle of Man"[tw] OR Jersey[tw] OR Guernsey[tw] OR "Channel Islands"[tw] OR Aberdeen[tw] OR Aberdeenshire[tw] OR Angus[tw] OR Forfarshire[tw] OR Argyll[tw] OR Ayrshire[tw] OR Banffshire[tw] OR Berwickshire[tw] OR Bute[tw] OR Caithness[tw] OR Clackmannanshire[tw] OR Cromartyshire[tw] OR Dumfriesshire[tw] OR Dunbartonshire[tw] OR Dumbarton[tw] OR Dundee[tw] OR Lothian[tw] OR Haddingtonshire[tw] OR Edinburgh[tw] OR Fife[tw] OR Glasgow[tw] OR Inverness-shire[tw] OR Kincardineshire[tw] OR Kinross-shire[tw] OR Kirkcudbrightshire[tw] OR Lanarkshire[tw] OR Midlothian[tw] OR Moray[tw] OR Elginshire[tw] OR Nairnshire[tw] OR Orkney[tw] OR Peeblesshire[tw] OR Perthshire[tw] OR Renfrewshire[tw] OR (Ross[tw] AND Cromarty[tw]) OR Ross-shire[tw] OR Roxburghshire[tw] OR Selkirkshire[tw] OR Shetland[tw] OR Zetland[tw] OR Stirlingshire[tw] OR Sutherland[tw] OR Linlithgowshire[tw] OR Wigtownshire[tw] OR Anglesey[tw] OR Brecknockshire[tw] OR Caernarfonshire[tw] OR Carmarthenshire[tw] OR Cardiganshire[tw] OR Ceredigion[tw] OR Clwyd[tw] OR Denbighshire[tw] OR Dyfed[tw] OR Flintshire[tw] OR Glamorgan[tw] OR Gwent[tw] OR Gwynedd[tw] OR Merionethshire[tw] OR Montgomeryshire[tw] OR Monmouthshire[tw] OR Pembrokeshire[tw] OR Powys[tw] OR Radnorshire[tw] OR Antrim[tw] OR Aontroim[tw] OR "Contae Aontroma"[tw] OR Anthrim[tw] OR Antrim[tw] OR Entrim[tw] OR Armagh[tw] OR "Ard Mhacha"[tw] OR Airmagh[tw] OR Belfast[tw] OR (Down[tw] AND (district[tw] OR council[tw] OR County[tw])) OR "An Dun"[tw] OR "an Duin"[tw] OR Doon[tw] OR Doun[tw] OR Fermanagh[tw] OR "Fear Manach"[tw] OR "Fhear Manach"[tw] OR Fermanay[tw] OR Londonderry[tw] OR Doire[tw] OR Dhoire[tw] OR Lunnonderrie[tw] OR Derry[tw] OR Birmingham[tw] OR Leeds[tw] OR Sheffield[tw] OR Bradford[tw] OR Liverpool[tw]))) OR ((GB[ad] OR "United kingdom"[ad] OR UK[ad] OR Britain[ad] OR British[ad] OR England[ad] OR English[ad] OR Scotland[ad] OR Scottish[ad] OR Scots[ad] OR Wales[ad] OR Cymru[ad] OR Welsh[ad] OR "North Ireland"[ad] OR "Northern Ireland"[ad] OR Irish[ad] OR Avon[ad] OR Bedfordshire[ad] OR Berkshire[ad] OR Bristol[ad] OR Buckinghamshire[ad] OR Cambridgeshire[ad] OR "Isle of Ely"[ad] OR Cheshire[ad] OR Cleveland[ad] OR Cornwall[ad] OR Cumberland[ad] OR Cumbria[ad] OR Derbyshire[ad] OR Devon[ad] OR Dorset[ad] OR Durham[ad] OR Essex[ad] OR Gloucestershire[ad] OR Hampshire[ad] OR Southampton[ad] OR (Hereford[ad] AND Worcester[ad]) OR Hertfordshire[ad] OR Herefordshire[ad] OR Humberside[ad] OR Huntingdon[ad] OR Huntingdonshire[ad] OR "Isle of Wight"[ad] OR Kent[ad] OR Lancashire[ad] OR Leicestershire[ad] OR Lincolnshire[ad] OR London[ad] OR Manchester[ad] OR Merseyside[ad] OR Middlesex[ad] OR Norfolk[ad] OR Northamptonshire[ad] OR Northumberland[ad] OR Nottinghamshire[ad] OR Oxfordshire[ad] OR Peterborough[ad] OR Rutland[ad] OR Shropshire[ad] OR Salop[ad] OR Somerset[ad] OR Yorkshire[ad] OR Staffordshire[ad] OR Suffolk[ad] OR Surrey[ad] OR Sussex[ad] OR (Tyne[ad] AND Wear[ad]) OR Warwickshire[ad] OR midlands[ad] OR Westmorland[ad] OR Wiltshire[ad] OR Worcestershire[ad] OR "Isle of Man"[ad] OR Jersey[ad] OR Guernsey[ad] OR "Channel Islands"[ad] OR Aberdeen[ad] OR Aberdeenshire[ad] OR Angus[ad] OR Forfarshire[ad] OR Argyll[ad] OR Ayrshire[ad] OR Banffshire[ad] OR Berwickshire[ad] OR bute[ad] OR Caithness[ad] OR Clackmannanshire[ad] OR Cromartyshire[ad] OR Dumfriesshire[ad] OR Dunbartonshire[ad] OR Dumbarton[ad] OR Dundee[ad] OR Lothian[ad] OR Haddingtonshire[ad] OR Edinburgh[ad] OR Fife[ad] OR Glasgow[ad] OR Inverness-shire[ad] OR Kincardineshire[ad] OR Kinross-shire[ad] OR Kirkcudbrightshire[ad] OR Lanarkshire[ad] OR Midlothian[ad] OR Moray[ad] OR Elginshire[ad] OR Nairnshire[ad] OR Orkney[ad] OR Peeblesshire[ad] OR Perthshire[ad] OR Renfrewshire[ad] OR (Ross[ad] AND Cromarty[ad]) OR Ross-shire[ad] OR Roxburghshire[ad] OR Selkirkshire[ad] OR Shetland[ad] OR Zetland[ad] OR Stirlingshire[ad] OR Sutherland[ad] OR Linlithgowshire[ad] OR Wigtownshire[ad] OR Anglesey[ad] OR Brecknockshire[ad] OR Caernarfonshire[ad] OR Carmarthenshire[ad] OR Cardiganshire[ad] OR Ceredigion[ad] OR Clwyd[ad] OR Denbighshire[ad] OR Dyfed[ad] OR Flintshire[ad] OR Glamorgan[ad] OR Gwent[ad] OR Gwynedd[ad] OR Merionethshire[ad] OR Montgomeryshire[ad] OR Monmouthshire[ad] OR Pembrokeshire[ad] OR Powys[ad] OR Radnorshire[ad] OR Antrim[ad] OR Aontroim[ad] OR "Contae Aontroma"[ad] OR Anthrim[ad] OR Antrim[ad] OR Entrim[ad] OR Armagh[ad] OR "Ard Mhacha"[ad] OR Airmagh[ad] OR Belfast[ad] OR (Down[ad] AND (district[ad] OR council[ad] OR County[ad])) OR "An Dun"[ad] OR "an Duin"[ad] OR Doon[ad] OR Doun[ad] OR Fermanagh[ad] OR "Fear Manach"[ad] OR "Fhear Manach"[ad] OR Fermanay[ad] OR Londonderry[ad] OR Doire[ad] OR Dhoire[ad] OR Lunnonderrie[ad] OR Derry[ad] OR Birmingham[ad] OR Leeds[ad] OR Sheffield[ad] OR Bradford[ad] OR Liverpool[ad]) OR (Sweden[ad] OR Sverige[ad] OR Swedish[ad] OR Svenska[ad] OR svenskar[ad] OR Swede[ad] OR Swedes[ad] OR Norrland[ad] OR Mellansverige[ad] OR Smaland[ad] OR Stockholm*[ad] OR Sydsverige[ad] OR Vastsverige[ad] OR Blekinge[ad] OR Dalarna[ad] OR Gavleborg*[ad] OR Gotland*[ad] OR Halland*[ad] OR Jamtland*[ad] OR Jonkoping*[ad] OR Kalmar[ad] OR Kronoberg*[ad] OR Norrbotten*[ad] OR Orebro[ad] OR Ostergotland*[ad] OR Skane[ad] OR Sodermanlands[ad] OR Uppsala[ad] OR Varmland*[ad] OR Vasterbotten*[ad] OR Vasternorrland*[ad] OR Vastmanland*[ad] OR vastergotland*[ad] OR Gotaland*[ad] OR Gothenburg[ad] OR Goteborg[ad] OR Malmo[ad] OR Vasteras[ad] OR Linkoping[ad] OR Helsingborg[ad] OR Halsingborg[ad] OR Norrkoping[ad]) OR (Spain[ad] OR Espana[ad] OR Spanish[ad] OR Espanol*[ad] OR Spaniard*[ad] OR Andalucia[ad] OR Andalusia[ad] OR Aragon[ad] OR Arago[ad] OR Cantabria[ad] OR Canarias[ad] OR "Canary Islands"[ad] OR (Canaries[ad] AND island*[ad]) OR "Castile and leon"[ad] OR "Castilla y Leon"[ad] OR "Castile La Mancha"[ad] OR "Castilla La Mancha"[ad] OR Cataluna[ad] OR Catalonia[ad] OR Ceuta[ad] OR Madrid[ad] OR Melilla[ad] OR Navarra[ad] OR Navarre[ad] OR Valencia*[ad] OR Extremadura[ad] OR Galicia[ad] OR Balears[ad] OR "Balearic Islands"[ad] OR "Balear Islands"[ad] OR Baleares[ad] OR "La Rioja"[ad] OR "Pais Vasco"[ad] OR "Basque Country"[ad] OR "Baske region"[ad] OR Euskadi[ad] OR Asturias[ad] OR Murcia[ad] OR Coruna[ad] OR Alava[ad] OR Araba[ad] OR Albacete[ad] OR Alicante[ad] OR Alacant[ad] OR Almeria[ad] OR Avila[ad] OR Badajoz[ad] OR Badajos[ad] OR Barcelona[ad] OR Burgos[ad] OR Caceres[ad] OR Cadiz[ad] OR Castellon[ad] OR Castello[ad] OR "Ciudad Real"[ad] OR Cordoba[ad] OR Cuenca[ad] OR Eivissa[ad] OR Ibiza[ad] OR Formentera[ad] OR "El Hierro"[ad] OR Fuerteventura[ad] OR Galiza[ad] OR Girona[ad] OR Gerona[ad] OR "Gran Canaria"[ad] OR Granada[ad] OR Guadalajara[ad] OR Guipuzcoa[ad] OR Gipuzkoa[ad] OR Huelva[ad] OR Huesca[ad] OR Jaen[ad] OR "La Gomera"[ad] OR "La Palma"[ad] OR Lanzarote[ad] OR Leon[ad] OR Lleida[ad] OR Lerida[ad] OR Lugo[ad] OR Malaga[ad] OR Mallorca[ad] OR Majorca[ad] OR Menorca[ad] OR Minorca[ad] OR Murcia[ad] OR Ourense[ad] OR Orense[ad] OR Palencia[ad] OR Pontevedra[ad] OR Salamanca[ad] OR Segovia[ad] OR Sevilla[ad] OR Seville[ad] OR Soria[ad] OR Tarragona[ad] OR Tenerife[ad] OR Teruel[ad] OR Toledo[ad] OR Valladolid[ad] OR Vizcaya[ad] OR Biscay[ad] OR Zamora[ad] OR Zaragoza[ad] OR Saragossa[ad] OR "Las Palmas"[ad] OR Bilbao[ad] OR Bilbo[ad]) OR (Slovenia*[ad] OR Slovenija[ad] OR slovensk*[ad] OR Slovenci[ad] OR Slovene*[ad] OR Gorenjska[ad] OR Carniola[ad] OR Goriska[ad] OR Gorizia[ad] OR Jugovzhodna[ad] OR Koroska[ad] OR Carinthia[ad] OR "Notranjsko kraska"[ad] OR "Obalno kraska"[ad] OR "Coastal karst"[ad] OR Osrednjeslovenska[ad] OR Podravska[ad] OR Drava[ad] OR Pomurska[ad] OR Mura[ad] OR Savinjska[ad] OR Savinja[ad] OR Spodnjeposavska[ad] OR Zasavska[ad] OR "Central Sava"[ad] OR Posavska[ad] OR "Lower Sava"[ad] OR Ljubljana[ad] OR Laibach[ad] OR Lubiana[ad] OR Maribor[ad] OR "Marburg an der Drau"[ad] OR Kranj[ad] OR Carnium[ad] OR Creina[ad] OR Chreina[ad] OR Krainbur[ad] OR Koper[ad] OR Capodistria[ad] OR Kopar[ad] OR Celje[ad] OR "Novo mesto"[ad] OR Neustadtl[ad] OR Domzale[ad] OR Velenje[ad] OR Wollan[ad] OR Woellan[ad] OR "Nova Gorica"[ad] OR Kamnik[ad]) OR (Slovakia[ad] OR Slovensk*[ad] OR Slovak*[ad] OR Slovaci[ad] OR Slovenki[ad] OR Bratislav*[ad] OR Presporok[ad] OR Pressburg[ad] OR Pressburg[ad] OR Posonium[ad] OR Banskobystri*[ad] OR "Banska Bystrica"[ad] OR Neusohl[ad] OR Besztercebanya[ad] OR Kosic*[ad] OR Kaschau[ad] OR Kassa[ad] OR Nitrian*[ad] OR Nitra[ad] OR Neutra[ad] OR Nyitra[ad] OR Nyitria[ad] OR Trnav*[ad] OR Tyrnau[ad] OR Nagyszombat[ad] OR Tyrnavia[ad] OR Presov*[ad] OR Trencian*[ad] OR Trencin[ad] OR Trentschin[ad] OR Trencsen[ad] OR Zilina[ad] OR Sillein[ad] OR Zsolna[ad] OR Zylina[ad] OR (Martin[ad] AND (city[ad] OR Svaty[ad])) OR Turocszentmarton[ad] OR Poprad[ad] OR Deutschendorf[ad] OR Zvolen[ad]) OR (Romania*[ad] OR Rumania*[ad] OR Roumania*[ad] OR Romani[ad] OR Rumani[ad] OR Alba[ad] OR Arad[ad] OR Arges[ad] OR Bacau[ad] OR Bihor[ad] OR "Bistrita Nasaud"[ad] OR Botosani[ad] OR Braila[ad] OR Brasov[ad] OR Kronstadt[ad] OR Brasso[ad] OR Brassovia[ad] OR Coron[ad] OR Bucharest[ad] OR Bucuresti[ad] OR Buzau[ad] OR Calarasi[ad] OR "Caras-Severin"[ad] OR Cluj[ad] OR Klausenburg[ad] OR Kolozsvar[ad] OR Constanta[ad] OR Tomis[ad] OR Konstantia[ad] OR Kostence[ad] OR Covasna[ad] OR Dambovita[ad] OR Dolj[ad] OR Galati[ad] OR Galatz[ad] OR Galac[ad] OR Kalas[ad] OR Giurgiu[ad] OR Gorj[ad] OR Harghita[ad] OR Hunedoara[ad] OR Ialomita[ad] OR Iasi[ad] OR Jassy[ad] OR Lassy[ad] OR Ilfov[ad] OR Maramures[ad] OR Mehedinti[ad] OR Mures[ad] OR Neamt[ad] OR (Olt[ad] AND (river[ad] OR county[ad] OR region[ad] OR judetul[ad] OR Raul[ad])) OR Prahova[ad] OR Salaj[ad] OR "Satu Mare"[ad] OR Sibiu[ad] OR Suceava[ad] OR Teleorman[ad] OR Timis[ad] OR Tulcea[ad] OR Valcea[ad] OR Vilcea[ad] OR Vaslui[ad] OR Vrancea[ad] OR Timisoara[ad] OR Temeswar[ad] OR Temeschburg[ad] OR Temeschwar[ad] OR Temesvar[ad] OR Temisvar[ad] OR Timisvar[ad] OR Temesva[ad] OR Craiova[ad] OR Ploiesti[ad] OR

Ploesti[ad] OR Oradea[ad] OR Varad[ad] OR Varat[ad]) OR (Portugal[ad] OR Portugues*[ad] OR Azores[ad] OR Acores[ad] OR Madeira[ad] OR Alentejo[ad] OR Algarve[ad] OR Lisboa[ad] OR Lisbon[ad] OR "Alto Tras-os-Montes"[ad] OR (Ave[ad] AND (community[ad] OR intermunicipal[ad] OR comunidade[ad])) OR Mondego[ad] OR Vouga[ad] OR Beira[ad] OR Cavado[ad] OR Lafoes[ad] OR Douro[ad] OR Porto[ad] OR Oporto[ad] OR Tejo[ad] OR Minho[ad] OR Setubal[ad] OR Pinhal[ad] OR "Serra da Estrela"[ad] OR Tamega[ad] OR Leira[ad] OR Santarem[ad] OR Beja[ad] OR Faro[ad] OR Evora[ad] OR Portalegre[ad] OR "Castelo Branco"[ad] OR Guarda[ad] OR Cimbra[ad] OR Aveiro[ad] OR Viseu[ad] OR Braganca[ad] OR Braganza[ad] OR Braga[ad] OR "Vila real"[ad] OR "Viana do Castelo"[ad] OR Gaia[ad] OR Amadora[ad] OR Funchal[ad] OR Coimbra[ad] OR Almada[ad] OR (Agualva[ad] AND Cacem[ad])) OR (Poland[ad] OR Polska[ad] OR Polish[ad] OR Pole[ad] OR Poles[ad] OR Polski[ad] OR Polak[ad] OR Polka[ad] OR Polacy[ad] OR Dolnoslaskie[ad] OR Silesia*[ad] OR Slask[ad] OR Pomorskie[ad] OR Pomerania*[ad] OR Kujawsko[ad] OR Kuyavian[ad] OR Lodzkie[ad] OR Lodz[ad] OR Lubelskie[ad] OR Lublin[ad] OR Lubuskie[ad] OR Lubusz[ad] OR Lubus[ad] OR Malopolskie[ad] OR Mazowieckie[ad] OR Mazowske[ad] OR Masovia[ad] OR Masovian[ad] OR Opolskie[ad] OR Opole[ad] OR Podkarpackie[ad] OR Subcarpathian*[ad] OR Podlaskie[ad] OR Podlachia[ad] OR Podlasie[ad] OR Slaskie[ad] OR Swietokrzyskie[ad] OR "Varmia Mazuria"[ad] OR "Varmian Mazurian"[ad] OR "Varmia Masuria"[ad] OR "Varmian Masurian"[ad] OR "Warmia Mazury"[ad] OR "Warminsko Mazurskie"[ad] OR "Warmian Masurian"[ad] OR Wielkopolskie[ad] OR Zachodniopomorskie[ad] OR Warsaw[ad] OR Warszawa[ad] OR Krakow[ad] OR Cracow[ad] OR Wroclaw[ad] OR Poznan[ad] OR Gdansk[ad] OR Szczecin[ad] OR Bydgoszcz[ad] OR Katowice[ad]) OR (Netherlands[ad] OR Nederland*[ad] OR Dutch*[ad] OR Drenthe[ad] OR Flevoland[ad] OR Friesland[ad] OR Fryslan[ad] OR Frisia[ad] OR Gelderland[ad] OR Guelders[ad] OR Groningen[ad] OR Limburg[ad] OR Brabant[ad] OR Holland[ad] OR Overijssel[ad] OR Overissel[ad] OR Utrecht[ad] OR Zeeland[ad] OR Amsterdam[ad] OR Rotterdam[ad] OR Hague[ad] OR "s-Gravenhage"[ad] OR "Den Haag"[ad] OR Eindhoven[ad] OR Tilburg[ad] OR Almere[ad] OR Breda[ad] OR Nijmegen[ad] OR Nimeguen[ad]) OR (Malta[ad] OR Maltese*[ad] OR Maltin[ad] OR Gozo[ad] OR Ghawdex[ad] OR Valletta[ad] OR "Ill Belt"[ad] OR Birkirkara[ad] OR "B Kara"[ad] OR "B'Kara"[ad] OR Birchircara[ad] OR Mosta[ad] OR Qormi[ad] OR "St Pauls Bay"[ad] OR "St Paul's Bay"[ad] OR "Pawl il Bahar"[ad] OR Zabbar[ad] OR Sliema[ad] OR Naxxar[ad] OR Gwann[ad] OR "St John"[ad] OR Zebbug[ad] OR "Citta rohan"[ad] OR Fgura[ad]) OR (Luxembourg*[ad] OR Luxemburg[ad] OR Letzebuerg[ad] OR Diekirch[ad] OR Grevenmacher[ad] OR "Esch sur Alzette"[ad] OR "Esch Uelzecht"[ad] OR "Esch an der Alzette"[ad] OR "Esch an der Alzig"[ad] OR Dudelange[ad] OR Diddeleng[ad] OR Dudelingen[ad] OR Duedelingen[ad] OR Schifflange[ad] OR Scheffleng[ad] OR Schifflingen[ad] OR Bettembourg[ad] OR Beetebuerg[ad] OR Bettemburg[ad] OR Petange[ad] OR Peiteng[ad] OR Petingen[ad] OR Ettelbruck[ad] OR Ettelbreck[ad] OR Ettelbrueck[ad] OR Diekirch[ad] OR Dikrech[ad] OR Strassen[ad] OR Stroossen[ad] OR Bertrange[ad] OR Bartreng[ad] OR Bartringen[ad]) OR (Lithuania*[ad] OR "Lietuvos Respublika"[ad] OR Lietuva[ad] OR lietuviu[ad] OR Alytus[ad] OR Alytaus[ad] OR Kaunas[ad] OR Kauno[ad] OR Klaipeda[ad] OR Klaipedos[ad] OR Marijampoles[ad] OR Marijampole[ad] OR Panevezys[ad] OR Panevezio[ad] OR Siauliai[ad] OR Siauliu[ad] OR Taurages[ad] OR Taurage[ad] OR Telsiu[ad] OR Telsiai[ad] OR Utenos[ad] OR Utena[ad] OR Vilnius[ad] OR Vilniaus[ad] OR Mazeikiai[ad] OR Jonava[ad] OR Mazeikiu[ad] OR Jonavos[ad]) OR (Latvi*[ad] OR Riga[ad] OR Courland[ad] OR Kurzeme[ad] OR Kurland[ad] OR Latgale[ad] OR Lettgallia[ad] OR Latgola[ad] OR Latgalia[ad] OR Vidzeme[ad] OR Vidumo[ad] OR Semigallia[ad] OR Semigalia[ad] OR Zemgale[ad] OR Pieriga[ad] OR Daugavpils[ad] OR Dinaburg[ad] OR Jekabpils[ad] OR Jakobstadt[ad] OR Jelgava[ad] OR Jurmala[ad] OR Liepaja[ad] OR Libau[ad] OR Rezekne[ad] OR Rezne[ad] OR Rositten[ad] OR Valmiera[ad] OR Wolmar[ad] OR Ventspils[ad] OR Windau[ad] OR Ogre[ad]) OR (Italy[ad] OR Italia*[ad] OR Abruzzo[ad] OR Abruzzi[ad] OR Basilicata[ad] OR Lucania[ad] OR Calabria[ad] OR Campania[ad] OR "Emilia Romagna"[ad] OR "friuli venezia giulia"[ad] OR Lazio[ad] OR Latium[ad] OR Liguria*[ad] OR Lombardy[ad] OR Lombardia[ad] OR Marche[ad] OR Marches[ad] OR Molisano[ad] OR Molise[ad] OR Piedmont*[ad] OR Piemonte[ad] OR Bolzano[ad] OR Bozen[ad] OR Trentino[ad] OR Trento[ad] OR Puglia[ad] OR Apulia[ad] OR Sardinia[ad] OR Sardegna[ad] OR Sicily[ad] OR Sicilia[ad] OR Toscana[ad] OR Tuscany[ad] OR Umbria[ad] OR "Valle d Aosta"[ad] OR "Vallee d Aoste"[ad] OR "Valle d'Aosta"[ad] OR "Vallee d'Aoste"[ad] OR "Aosta Valley"[ad] OR Veneto[ad] OR Venetia[ad] OR Triveneto[ad] OR Rome[ad] OR Roma[ad] OR Milan[ad] OR Milano[ad] OR Naples[ad] OR Napoli[ad] OR Turin[ad] OR Torino[ad] OR Palermo[ad] OR Genoa[ad] OR Genova[ad] OR Bologna[ad] OR Florence[ad] OR Firenze[ad] OR Bari[ad] OR Catania[ad]) OR (Ireland[ad] OR Eire[ad] OR Irish*[ad] OR Fingal[ad] OR "Fine Gall"[ad] OR Dublin[ad] OR "Ath Cliath"[ad] OR "Dun Laoghaire"[ad] OR Wicklow[ad] OR "Cill Mhantain"[ad] OR "Chill Mhantain"[ad] OR Wexford[ad] OR "Loch Garman"[ad] OR Carlow[ad] OR Ceatharlach[ad] OR Kildare[ad] OR "Cill Dara"[ad] OR "Chill Dara"[ad] OR Meath[ad] OR "An Mhi"[ad] OR "Contae na Mi"[ad] OR Louth[ad] OR "Contae Lu"[ad] OR Monaghan[ad] OR Muineachan[ad] OR Mhuineachain[ad] OR Cavan[ad] OR "An Cabhan"[ad] OR "An Cabhain"[ad] OR Longford[ad] OR "An Longfort"[ad] OR "an Longfoirt"[ad] OR Langfurd[ad] OR Westmeath[ad] OR "An Iarmhi"[ad] OR "na Iarmhi"[ad] OR Offaly[ad] OR "Uibh Fhaili"[ad] OR Laois[ad] OR Laoise[ad] OR Kilkenny[ad] OR "Chill Chainnigh"[ad] OR "Cill Chainnigh"[ad] OR Waterford[ad] OR "Port Lairge"[ad] OR Watterford[ad] OR Cork[ad] OR Corcaigh[ad] OR Chorcai[ad] OR Kerry[ad] OR Ciarrai[ad] OR Chiarrai[ad] OR Limerick[ad] OR Luimneach[ad] OR Luimnigh[ad] OR Tipperary[ad] OR "Tiobraid Arann"[ad] OR "Thiobraid Arann"[ad] OR Clare[ad] OR "An Clar"[ad] OR "an Chlair"[ad] OR Galway[ad] OR Gaillimh[ad] OR "na Gaillimhe"[ad] OR Mayo[ad] OR "Maigh Eo"[ad] OR "Mhaigh Eo"[ad] OR Roscommon[ad] OR "Ros comain"[ad] OR Sligo[ad] OR Sligeach[ad] OR Shligigh[ad] OR Leitrim[ad] OR Liatroim[ad] OR Liatroma[ad] OR Donegal[ad] OR "Dhun na nGall"[ad] OR Dinnygal[ad] OR Dunnyga[ad] OR Leinster[ad] OR Laighin[ad] OR "Cuige Laighean"[ad] OR Munster[ad] OR Mumhain[ad] OR "Cuige Mumhan"[ad] OR Connacht[ad] OR Connachta[ad] OR Drogheda[ad] OR "Droichead Atha"[ad] OR Dundalk[ad] OR "Dun Dealgan"[ad] OR Swords[ad] OR Sord[ad] OR Bray[ad] OR Bre[ad] OR Navan[ad] OR "An Uaimh"[ad]) OR (Hungar*[ad] OR Magyarorszag[ad] OR Magyar*[ad] OR Dunantuli[ad] OR Transdanubia[ad] OR Dunantul[ad] OR "Great Plain"[ad] OR "Eszak Alfold"[ad] OR "Del Alfold"[ad] OR "Alfold es eszak"[ad] OR "Northern Alfold"[ad] OR "North Alfold"[ad] OR "South Alfold"[ad] OR "Southern Alfold"[ad] OR Bacs[ad] OR Kiskun[ad] OR Baranya[ad] OR Bekes[ad] OR Borsod[ad] OR Abauj[ad] OR Zemplen[ad] OR Budapest[ad] OR Csongrad[ad] OR Fejer[ad] OR gyor[ad] OR moson[ad] OR sopron[ad] OR hajdu[ad] OR bihar[ad] OR Heves[ad] OR "jasz nagykun szolnok"[ad] OR komarom[ad] OR esztergom[ad] OR Nograd[ad] OR (Pest[ad] AND (megye[ad] OR county[ad])) OR Somogy[ad] OR szabolcs[ad] OR szatmar[ad] OR bereg[ad] OR Tolna[ad] OR Vas[ad] OR Veszprem[ad] OR Zala[ad] OR Debrecen[ad] OR Miskolc[ad] OR Szeged[ad] OR Pecs[ad] OR Gyor[ad] OR Nyiregyhaza[ad] OR Kecskemet[ad] OR Szekesfehervar[ad] OR Szombathely[ad]) OR (Greece[ad] OR "Hellenic republic"[ad] OR Greek*[ad] OR Ellada[ad] OR Elladas[ad] OR "Elliniki Dimokratia"[ad] OR Hellas[ad] OR Hellenes[ad] OR Attica[ad] OR Attiki[ad] OR Makedonia*[ad] OR Macedonia[ad] OR Thraki[ad] OR Thrace[ad] OR Crete[ad] OR Kriti[ad] OR "Ionia Nisia"[ad] OR "Ionion neson"[ad] OR "Ionion nIson"[ad] OR "Ionian islands"[ad] OR "Ionian island"[ad] OR Epirus[ad] OR Ipeiros[ad] OR "Perifereia Ipeirou"[ad] OR "North aegean"[ad] OR "Northern Aegean"[ad] OR "Aegean islands"[ad] OR "Aegean island"[ad] OR "Nisoi Agaiou"[ad] OR "Notio Aigaio"[ad] OR Peloponnese[ad] OR Peloponniso*[ad] OR Thessaly[ad] OR Thessalia[ad] OR Thessalian[ad] OR Petthalia[ad] OR "Voreio Aigaio"[ad] OR "Voreio Aigaiou"[ad] OR "South aegean"[ad] OR "Southern Aegean"[ad] OR "Mount athos"[ad] OR "Oros Athos"[ad] OR Cyclades[ad] OR Cycklades[ad] OR Kiklades[ad] OR Dodecanese[ad] OR Dodekanisa[ad] OR Athens[ad] OR Athina[ad] OR Thessaloniki[ad] OR Thessalonica[ad] OR Patras[ad] OR Patra[ad] OR Pireas[ad] OR Piraeus[ad] OR Larissa[ad] OR Larisa[ad] OR Heraklion[ad] OR Heraclion[ad] OR Iraklion[ad] OR Irakleion[ad] OR Iraklio[ad] OR Volos[ad] OR Rhodes[ad] OR Rodos[ad] OR Ioannina[ad] OR Janina[ad] OR Yannena[ad] OR Chania[ad] OR Chalcis[ad] OR Chalkida[ad]) OR (German*[ad] OR Deutsch*[ad] OR Bundesrepublik[ad] OR Westdeutschland[ad] OR Ostdeutschland[ad] OR Baden[ad] OR Wuerttemberg[ad] OR Wurttemberg[ad] OR Bayern[ad] OR Bavaria[ad] OR Berlin[ad] OR Brandenburg[ad] OR Bremen[ad] OR Oldenburg[ad] OR Mitteldeutschland[ad] OR Rhein[ad] OR Rhine[ad] OR Hannover[ad] OR Braunschweig[ad] OR Gottingen[ad] OR Goettingen[ad] OR Nurnberg[ad] OR Nuernberg[ad] OR Ruhr[ad] OR Koln[ad] OR koeln[ad] OR Bonn[ad] OR Hamburg[ad] OR Hessen[ad] OR Hesse[ad] OR Hessia[ad] OR Mecklenburg[ad] OR Vorpommern[ad] OR Pomerania[ad] OR Niedersachsen[ad] OR Neddersassen[ad] OR Saxony[ad] OR Niederbayern[ad] OR "Northern Rhine"[ad] OR "North Rhine"[ad] OR Westphalia[ad] OR Westfalen[ad] OR "Rhineland Palatinate"[ad] OR "Rheinland Pfalz"[ad] OR Saarland[ad] OR Sachsen[ad] OR "Schleswig Holstein"[ad] OR Thuringia[ad] OR Thuringen[ad] OR Thueringen[ad] OR Munchen[ad] OR Muenchen[ad] OR Munich[ad] OR Frankfurt[ad] OR Stuttgart[ad] OR Dusseldorf[ad] OR Duesseldorf[ad] OR Dortmund[ad] OR Essen[ad]) OR (France[ad] OR French*[ad] OR Francais*[ad] OR Alsace[ad] OR Elsass[ad] OR Aquitaine[ad] OR Aquitania[ad] OR Akitania[ad] OR Aguiene[ad] OR Auvergne[ad] OR Auvernhe[ad] OR Auvernha[ad] OR Normandie[ad] OR Normandy[ad] OR Normaundie[ad] OR Bourgogne[ad] OR Burgundy[ad] OR Bregogne[ad] OR Borgoegne[ad] OR Borgogne[ad] OR Brittany[ad] OR Breizh[ad] OR Bertaeyn[ad] OR Bretagne[ad] OR "Champagne Ardenne"[ad] OR Corse[ad] OR Corsica[ad] OR "Franche Comte"[ad] OR "Frantche Comte"[ad] OR "Franche Comtat"[ad] OR Guadeloupe[ad] OR Guyane[ad] OR Guiana[ad] OR "Languedoc Roussillon"[ad] OR "Lengadoc Rosselhon"[ad] OR "Llenguadoc-Rossello"[ad] OR Limousin[ad] OR Lemosin[ad] OR Lorraine[ad] OR Lothringen[ad] OR Lottringe[ad] OR Martinique[ad] OR "Midi Pyrenees"[ad] OR "Miegjorn Pireneus"[ad] OR "Mieidia Pireneus"[ad] OR "Mediodia Pirineos"[ad] OR "Pays de la Loire"[ad] OR "Broiou al Liger"[ad] OR Picardie[ad] OR Picardy[ad] OR "Poitou Charentes"[ad] OR "Peitau Charantas"[ad] OR "Poetou-Cherentes"[ad] OR Provence[ad] OR Provenca[ad] OR Prouvenco[ad] OR "Cote d Azur"[ad] OR "Cote d'Azur"[ad] OR "Costo d'Azur"[ad] OR "Costo d Azur"[ad] OR "Costa d'Azur"[ad] OR "Costa d Azur"[ad] OR Reunion[ad] OR "Rhone Alpes"[ad] OR "Rono Arpes"[ad] OR "Rose Aups"[ad] OR Ain[ad] OR Aisne[ad] OR Allier[ad] OR "Alpes de Haute Provence"[ad] OR "Haute Alpes"[ad] OR "Alpes Maritimes"[ad] OR Ardeche[ad] OR Ardennes[ad] OR Ariege[ad] OR Aube[ad] OR Aude[ad] OR Aveyron[ad] OR "Bas Rhin"[ad] OR "Bouches du Rhone"[ad] OR Calvados[ad] OR Cantal[ad] OR Charente[ad] OR Cher[ad] OR Correze[ad] OR "Corse du Sud"[ad] OR "Cote d Or"[ad] OR "Cote d'Or"[ad] OR "Cotes d Armor"[ad] OR "Cotes d'Armor"[ad] OR Creuse[ad] OR "Deux Sevres"[ad] OR Dordogne[ad] OR Doubs[ad] OR Drome[ad] OR Essonne[ad] OR Eure[ad] OR Finistere[ad] OR Gard[ad] OR Gers[ad] OR Gironde[ad] OR "Haute Corse"[ad] OR "Haute Garonne"[ad] OR "Haute Marne"[ad] OR "Hautes Alpes"[ad] OR "Haute Saone"[ad] OR "Haute Savoie"[ad] OR "Hautes Pyrenees"[ad] OR "Haute Vienne"[ad] OR "Haut Rhin"[ad] OR "Hauts de Seine"[ad] OR Herault[ad] OR "Ile de France"[ad] OR "Ille et Vilaine"[ad] OR Indre[ad] OR

Isere[ad] OR Jura[ad] OR Landes[ad] OR Loire[ad] OR Loiret[ad] OR (Lot[ad] AND (departement[ad] OR department[ad])) OR "Lot et Garonne"[ad] OR "Loir et Cher"[ad] OR Lozere[ad] OR Manche[ad] OR Marne[ad] OR Mayenne[ad] OR Mayotte[ad] OR "Meurthe et Moselle"[ad] OR Meuse[ad] OR Morbihan[ad] OR Moselle[ad] OR (Nord[ad] AND (department[ad] OR departement[ad])) OR Nievre[ad] OR Oise[ad] OR Orne[ad] OR "Pas de calais"[ad] OR "Noord-Nauw van Kales"[ad] OR Paris[ad] OR "Puy de dome"[ad] OR "Pyrenees Atlantiques"[ad] OR "Pyrenees Orientales"[ad] OR Rhone[ad] OR Sarthe[ad] OR Savoie[ad] OR "Seine et Marne"[ad] OR "Seine Maritime"[ad] OR Somme[ad] OR Tarn[ad] OR "Territoire de Belfort"[ad] OR "Val de Marne"[ad] OR "Val d Oise"[ad] OR Var[ad] OR Vaucluse[ad] OR Vendee[ad] OR Vienne[ad] OR Vosges[ad] OR Yonne[ad] OR Yvelines[ad] OR Marseille[ad] OR Lyon[ad] OR Nice[ad] OR Nantes[ad] OR Strasbourg[ad] OR Montpellier[ad] OR Bordeaux[ad] OR Lille[ad] OR Toulouse[ad] OR "Outre Mer"[ad] OR "Seine Saint Denis"[ad]) OR (Finland[ad] OR Finnish*[ad] OR Finn[ad] OR Finns[ad] OR Suomi[ad] AND Suomen[ad] OR Suomalaiset[ad] OR Aland[ad] OR Ahvenanmaa[ad] OR Uusimaa[ad] OR Nyland[ad] OR Karelia[ad] OR Karjala[ad] OR Karelen[ad] OR Ostrobothnia[ad] OR Pohjanmaa[ad] OR Osterbotten[ad] OR Savonia[ad] OR Savo[ad] OR Savolax[ad] OR Kainuu[ad] OR Kajanaland*[ad] OR "Kanta Hame"[ad] OR Tavastia[ad] OR Tavastland[ad] OR Kymenlaakso[ad] OR Kymmenedalen[ad] OR Lapland[ad] OR Lappi[ad] OR Lappland[ad] OR "Paijat Hame"[ad] OR Pirkanmaa[ad] OR Birkaland[ad] OR Satakunta[ad] OR Satakunda[ad] OR Helsinki[ad] OR Helsingfors[ad] OR Espoo[ad] OR Esbo[ad] OR Tampere[ad] OR Tammerfors[ad] OR Vantaa[ad] OR Vanda[ad] OR Oulu[ad] OR Uleaborg[ad] OR Turku[ad] OR Abo[ad] OR Jyvaskyla[ad] OR Kuopio[ad] OR Lahti[ad] OR Lahtis[ad] OR Kouvola[ad]) OR (Estonia*[ad] OR Eesti[ad] OR Eestlased[ad] OR Eestlane[ad] OR Harju[ad] OR Harjumaa[ad] OR Hiiu[ad] OR Hiiumaa[ad] OR "Ida Viru"[ad] OR "Ida Virumaa"[ad] OR Jarvamaa[ad] OR Jarva[ad] OR Jogevamaa[ad] OR Jogeva[ad] OR Laanemaa[ad] OR Laane[ad] OR "Laane Virumaa"[ad] OR Parnu[ad] OR Parnumaa[ad] OR Polva[ad] OR Polvamaa[ad] OR Rapla[ad] OR Raplamaa[ad] OR Saare[ad] OR Saaremaa[ad] OR Tartu[ad] OR Tartumaa[ad] OR Valga[ad] OR Valgamaa[ad] OR Viljandimaa[ad] OR Viljandi[ad] OR Voru[ad] OR Vorumaa[ad] OR Tallinn[ad] OR Narva[ad] OR "Kohtla Jarve"[ad] OR Rakvere[ad] OR Maardu[ad] OR Sillamae[ad] OR Kuressaare[ad]) OR (Denmark[ad] OR Danish*[ad] OR dane[ad] OR danes[ad] OR Danmark[ad] OR dansk*[ad] OR Hovedstaden[ad] OR Midtjylland[ad] OR Nordjylland[ad] OR Sjaelland[ad] OR Sealand[ad] OR "Zealand region"[ad] OR "region Zealand"[ad] OR Syddanmark[ad] OR Jutland[ad] OR Jylland[ad] OR Sonderjyllands[ad] OR Copenhagen[ad] OR Kobenhavn[ad] OR Arhus[ad] OR Aarhus[ad] OR Bornholm[ad] OR Frederiksberg[ad] OR Frederiksborg[ad] OR Ringkjobing[ad] OR Viborg[ad] OR Vejle[ad] OR Roskilde[ad] OR Storstrom[ad] OR Vestsjaellands[ad] OR "West Zealand"[ad] OR Funen[ad] OR Ribe[ad] OR "Kalaallit Nunaat"[ad] OR Gronland[ad] OR Foroyar[ad] OR Faeroerne[ad] OR "Faroe islands"[ad] OR Aalborg[ad] OR Alborg[ad] OR Odense[ad] OR Esbjerg[ad] OR Gentofte[ad] OR Gladsaxe[ad] OR Randers[ad] OR Kolding[ad]) OR (Czech*[ad] OR Cesky[ad] OR Ceska[ad] OR Cech[ad] OR Cestina[ad] OR Prague[ad] OR Praha[ad] OR Prag[ad] OR Stredoces*[ad] OR Jihoces*[ad] OR Bohemia[ad] OR Bohemian[ad] OR Plzen*[ad] OR Pilsen[ad] OR Karlovars*[ad] OR "Karlovy Vary"[ad] OR Usteck*[ad] OR Usti[ad] OR Liberec*[ad] OR "Hradec Kralove"[ad] OR Kralovehradec*[ad] OR Pardubic*[ad] OR Olomouc*[ad] OR Olomoc[ad] OR Holomoc[ad] OR Moravskoslezs*[ad] OR Jihomorav*[ad] OR Moravia[ad] OR Moravian[ad] OR Morava[ad] OR Vysocina[ad] OR Zlin[ad] OR Zlinsk*[ad] OR "Ceske Budejovice"[ad] OR Budweis[ad] OR Brno[ad] OR Ostrava[ad]) OR (Cyprus[ad] OR Cypriot*[ad] OR Kypros[ad] OR Kibris*[ad] OR kypriaki[ad] OR Kyprioi[ad] OR Nicosia[ad] OR Lefkosa[ad] OR Lefkosia[ad] OR Famagusta[ad] OR Magusa[ad] OR Ammochostos[ad] OR Gazimagusa[ad] OR Kyrenia[ad] OR Girne[ad] OR Keryneia[ad] OR Larnaca[ad] OR Larnaka[ad] OR Iskele[ad] OR Limassol[ad] OR Lemesos[ad] OR Limasol[ad] OR Leymosun[ad] OR Paphos[ad] OR Pafos[ad] OR Baf[ad] OR Strovolos[ad] OR Lakatamia[ad] OR Lakadamya[ad] OR "Kato Polemidia"[ad] OR "Kato Polemidhia"[ad] OR Aglandjia[ad] OR Eglence[ad] OR Aglantzia[ad] OR Aradhippou[ad] OR Aradippou[ad] OR Engomi[ad]) OR (Croat*[ad] OR Hrvatsk*[ad] OR hrvati[ad] OR Bjelovar[ad] OR "Bjelovarsko bilogorska"[ad] OR "Brod Posavina"[ad] OR "Brodsko posavska"[ad] OR "Dubrovnik Neretva"[ad] OR "dubrovacko neretvanska"[ad] OR Zagreb[ad] OR Zagrebacka[ad] OR Istria[ad] OR Istarska[ad] OR Karlovacka[ad] OR Karlovac[ad] OR "Koprivnicko krizevacka"[ad] OR Koprivnica[ad] OR Krizevci[ad] OR "Krapina Zagorje"[ad] OR "Krapinsko zagorska"[ad] OR "Lika Senj"[ad] OR "Licko senjska"[ad] OR Medimurska[ad] OR Medimurje[ad] OR Osijek[ad] OR Baranja[ad] OR "Osjecko baranjska"[ad] OR "Pozega Slavonia"[ad] OR "Pozesko slavonska"[ad] OR "Primorje Gorski Kotar"[ad] OR "Primorsko goranska"[ad] OR "Sibensko kninska"[ad] OR "Sibensko kninske"[ad] OR Sibenik[ad] OR Knin[ad] OR Sisak[ad] OR "Sisacko moslavacka"[ad] OR Moslavina[ad] OR "Splitsko dalmatinska"[ad] OR Split[ad] OR Dalmatia[ad] OR Varazdin[ad] OR Varazdinska[ad] OR Viroviticko[ad] OR podravska[ad] OR Virovitica[ad] OR Podravina[ad] OR "Vukovarsko srijemska"[ad] OR Vukovar[ad] OR Srijem[ad] OR Zadar[ad] OR Zadarska[ad] OR Rijeka[ad] OR "Velika gorica"[ad] OR "Slavonski brod"[ad] OR Pula[ad]) OR (Bulgaria*[ad] OR Balgariya[ad] OR Balgarija[ad] OR Blagoevgrad*[ad] OR "Pirin Macedonia"[ad] OR Burgas[ad] OR Dobrich[ad] OR Gabrovo[ad] OR Haskovo[ad] OR Kardzhali[ad] OR Kurdzhali[ad] OR Kyustendil[ad] OR Lovech[ad] OR Lovec[ad] OR Montana[ad] OR Pazardzhik[ad] OR Pernik[ad] OR Pleven*[ad] OR Plovdiv[ad] OR Razgrad[ad] OR Rousse[ad] OR Ruse[ad] OR Rusenka[ad] OR Shumen[ad] OR Silistra[ad] OR Sliven[ad] OR Smolyan[ad] OR Sofia[ad] OR Sofyiska[ad] OR Sofiiska[ad] OR "Stara Zagora"[ad] OR Targovishte[ad] OR Varna[ad] OR "Veliko Tarnovo"[ad] OR Vidin[ad] OR Vratsa[ad] OR Vratza[ad] OR Yambol[ad]) OR (Belgi*[ad] OR Belge*[ad] OR Belg[ad] OR Brussel*[ad] OR Bruxelles[ad] OR Bruxelloise[ad] OR Walloon*[ad] OR Wallon*[ad] OR Vlaams[ad] OR Flander*[ad] OR Flandern[ad] OR Flandre[ad] OR Flemish[ad] OR Flamand[ad] OR Flemisch[ad] OR Flamisch*[ad] OR Vlaanderen[ad] OR Flamande[ad] OR Waals[ad] OR Antwerp*[ad] OR Anvers[ad] OR Henegouwen[ad] OR Hennegau[ad] OR Hainault[ad] OR Hainaut[ad] OR Liege[ad] OR Luik[ad] OR Luttich[ad] OR Limbourg[ad] OR Limburg[ad] OR Namur[ad] OR Namen[ad] OR Ostflandern[ad] OR Westflandern[ad] OR Ghent[ad] OR Gent[ad] OR Gand[ad] OR Charleroi[ad] OR Bruges[ad] OR Brugge*[ad] OR Schaerbeek[ad] OR Schaarbeek[ad] OR Anderlecht[ad] OR Leuven[ad] OR Louvain[ad]) OR (Austria*[ad] OR Osterreich*[ad] OR Oesterreich*[ad] OR Ostosterreich[ad] OR Ostoesterreich[ad] OR Sudosterreich[ad] OR Sudoesterreich[ad] OR Westosterreich[ad] OR Westoesterreich[ad] OR Burgenland[ad] OR Carinthia[ad] OR Karnten[ad] OR Kaernten[ad] OR Niederosterreich[ad] OR Niederoesterreich[ad] OR Oberosterreich[ad] OR Oberoesterreich[ad] OR Salzburg[ad] OR Saizburg[ad] OR Styria[ad] OR Steiermark[ad] OR Tyrol[ad] OR Tirol[ad] OR Vorarlberg[ad] OR Vienna[ad] OR Wien[ad] OR Graz[ad] OR Linz[ad] OR Innsbruck[ad] OR Klagenfurt[ad] OR Villach[ad] OR Wels[ad] OR "St Polten"[ad] OR "St Poelten"[ad] OR "Sankt Polten"[ad] OR "Sankt Poelten"[ad] OR Dornbirn[ad]))

Embase search strings

String for Hepatitis B and Hepatitis C virus

('hepatitis B'/exp OR 'hepatitis B antibody'/exp OR 'hepatitis B antigen'/exp OR 'hepatitis b':ti,ab OR ‘hbv’:ti,ab OR 'hep b':ti,ab OR 'hbsag':ti,ab OR 'hbs ag':ti,ab OR 'hepatitis C'/exp OR 'Hepacivirus'/exp OR 'hepatitis C antibody'/exp OR 'hepatitis C antigen'/exp OR 'hepatitis c':ti,ab OR hepaciviru*:ti,ab OR 'hcv':ti,ab OR 'hep c':ti,ab OR 'blood borne virus':ti,ab OR 'blood borne viruses':ti,ab OR 'bbv':ti,ab)

String for occurrence

('prevalence'/exp OR 'incidence'/exp OR seroprevalen*:ti,ab OR prevalen*:ti,ab OR inciden*:ti,ab OR distribution*:ti,ab OR frequenc*:ti,ab OR ‘occurrence’:ti,ab OR ‘positivity rate’:ti,ab OR ‘positivity rates’:ti,ab OR ‘odds ratio’:ti,ab OR ‘odds ratios’:ti,ab OR ‘relative risk’:ti,ab OR ‘relative risks’:ti,ab OR ‘transmission rate’:ti,ab OR ‘transmission rates’:ti,ab OR ‘at risk’:ti,ab)

String for risk groups not covered in previous reviews

(‘health care staff’:ti,ab OR ‘healthcare staff’:ti,ab OR ‘health staff’:ti,ab OR ‘health worker’:ti,ab OR ‘health workers’:ti,ab OR ‘health care worker’:ti,ab OR ‘health care workers’:ti,ab OR ‘healthcare worker’:ti,ab OR ‘healthcare workers’:ti,ab OR ‘health care provider’:ti,ab OR ‘healthcare provider’:ti,ab OR ‘health care providers’:ti,ab OR ‘healthcare providers’:ti,ab OR ‘medical staff’:ti,ab OR ‘exposure prone procedure’:ti,ab OR ‘exposure prone procedures’:ti,ab OR ‘safety worker’:ti,ab OR ‘safety workers’:ti,ab OR police*:ti,ab OR firefighter*:ti,ab OR ‘firemen’:ti,ab OR ‘fireman’:ti,ab OR paramedic*:ti,ab OR ‘ambulance’:ti,ab OR ‘corrections officer’:ti,ab OR ‘corrections officers’:ti,ab OR ‘correctional officer’:ti,ab OR ‘correctional officers’:ti,ab OR ‘prison guard’:ti,ab OR ‘prison guards’:ti,ab OR ‘waste worker’:ti,ab OR ‘waste workers’:ti,ab OR ‘waste disposal’:ti,ab OR ‘waste disposer’:ti,ab OR ‘waste disposers’:ti,ab OR ‘waste collection’:ti,ab OR ‘waste collector’:ti,ab OR ‘waste collectors’:ti,ab OR ‘waste removal’:ti,ab OR ‘waste remover’:ti,ab OR ‘waste removers’:ti,ab OR ‘sewage worker’:ti,ab OR ‘sewage workers’:ti,ab OR ‘rubbish men’:ti,ab OR ‘rubbish man’:ti,ab OR rubbishm*:ti,ab OR ‘rubbish disposal’:ti,ab OR ‘rubbish disposer’:ti,ab OR ‘rubbish disposers’:ti,ab OR ‘rubbish collection’:ti,ab OR ‘rubbish collector’:ti,ab OR ‘rubbish collectors’:ti,ab OR ‘rubbish removal’:ti,ab OR ‘rubbish remover’:ti,ab OR ‘rubbish removers’:ti,ab OR ‘bin man’:ti,ab OR ‘bin men’:ti,ab OR ‘dustbin man’:ti,ab OR ‘dustbin men’:ti,ab OR ‘garbage men’:ti,ab OR ‘garbage man’:ti,ab OR garbagem*:ti,ab OR ‘garbage disposal’:ti,ab OR ‘garbage disposer’:ti,ab OR ‘garbage disposers’:ti,ab OR ‘garbage collection’:ti,ab OR ‘garbage collector’:ti,ab OR ‘garbage collectors’:ti,ab OR ‘garbage worker’:ti,ab OR ‘garbage workers’:ti,ab OR ‘garbage removal’:ti,ab OR ‘garbage remover’:ti,ab OR ‘garbage removers’:ti,ab OR ‘trash men’:ti,ab OR ‘trash man’:ti,ab OR trashm*:ti,ab OR ‘trash disposal’:ti,ab OR ‘trash disposer’:ti,ab OR ‘trash disposers’:ti,ab OR ‘trash collection’:ti,ab OR ‘trash collector’:ti,ab OR ‘trash collectors’:ti,ab OR ‘trash worker’:ti,ab OR ‘trash workers’:ti,ab OR ‘trash removal’:ti,ab OR ‘trash remover’:ti,ab OR ‘trash removers’:ti,ab OR ‘refuse disposal’:ti,ab OR ‘refuse disposer’:ti,ab OR ‘refuse disposers’:ti,ab OR ‘refuse collection’:ti,ab OR ‘refuse collector’:ti,ab OR ‘refuse collectors’:ti,ab OR ‘refuse worker’:ti,ab OR ‘refuse workers’:ti,ab OR ‘refuse removal’:ti,ab OR ‘refuse

remover’:ti,ab OR ‘refuse removers’:ti,ab OR ‘HIV infected’:ti,ab OR ‘HIV positive’:ti,ab OR ‘HIV seropositive’:ti,ab OR ‘HIV patient’:ti,ab OR ‘HIV patients’:ti,ab OR ‘infected with HIV’:ti,ab OR ‘STI patient’:ti,ab OR ‘STI patients’:ti,ab OR ‘STD patient’:ti,ab OR ‘STD patients’:ti,ab OR ‘STI infected’:ti,ab OR ‘STD infected’:ti,ab OR ‘intranasal drug’:ti,ab OR ‘intranasal drugs’:ti,ab OR ‘snort drugs’:ti,ab OR ‘inhale drugs’:ti,ab OR 'blood transfusion'/exp OR ‘blood transfusion’:ti,ab OR ‘blood transfusions’:ti,ab OR 'graft recipient'/exp OR transplant*:ti,ab OR 'hemodialysis'/exp OR dialys*:ti,ab OR hemodialys*:ti,ab OR haemodialys*:ti,ab OR ‘medical intervention’:ti,ab OR ‘medical interventions’:ti,ab OR ‘dental’:ti,ab OR ‘dentistry’:ti,ab OR ‘surgery’:ti,ab OR ‘medical procedure’:ti,ab OR ‘medical procedures’:ti,ab OR 'body modification'/exp OR tattoo*:ti,ab OR pierc*:ti,ab OR scarifi*:ti,ab OR ‘body modification’:ti,ab OR acupunctur*:ti,ab OR ‘mesotherapy’:ti,ab OR ‘care home’:ti,ab OR ‘care homes’:ti,ab OR institutionali*:ti,ab OR ‘residential care’:ti,ab OR 'mentally disabled person'/exp OR ‘mentally disabled’:ti,ab OR ‘mental disability’:ti,ab OR ‘mental disabilities’:ti,ab OR ‘intellectual disability’:ti,ab OR ‘intellectual disabilities’:ti,ab OR ‘intellectually disabled’:ti,ab OR ‘low intellect’:ti,ab OR ‘learning disability’:ti,ab OR ‘learning disabilities’:ti,ab OR retard*:ti,ab OR ‘cognitive disability’:ti,ab OR ‘cognitive disabilities’:ti,ab OR ‘intellectual impairment’:ti,ab OR ‘mental deficiency’:ti,ab OR homeless*:ti,ab OR vagrant*:ti,ab OR partner*:ti,ab OR couple*:ti,ab OR 'nuclear family'/exp OR 'spouse'/exp OR spouse*:ti,ab OR husband*:ti,ab OR ‘wife’:ti,ab OR ‘wives’:ti,ab OR ‘household’:ti,ab OR contact*:ti,ab OR mother*:ti,ab OR ‘birth cohort’:ti,ab OR 'travel'/exp OR travel*:ti,ab OR 'rape'/exp OR rape*:ti,ab OR assault*:ti,ab OR ‘multiple sexual partners’:ti,ab OR ‘multiple sex partners’:ti,ab OR ‘multiple sexual contacts’:ti,ab OR ‘sexually promiscuous’:ti,ab OR ‘sexual promiscuity’:ti,ab OR diabet*:ti,ab OR auto-inject*:ti,ab OR transsexual*:ti,ab OR transgender*:ti,ab OR ‘anabolic steroid’:ti,ab OR ‘anabolic steroids’:ti,ab OR ‘performance enhancing’:ti,ab OR ‘beauty’:ti,ab OR salon*:ti,ab OR pedicur*:ti,ab OR manicur*:ti,ab)

String for EU/EEA

'european union'/exp OR 'europe'/de OR europe*:ab,ti OR europa*:ab,ti OR eu:ab,ti OR eea:ab,ti OR efta:ab,ti OR 'eu/eea':ab,ti OR 'eu/efta':ab,ti OR ecsc:ab,ti OR euratom:ab,ti OR eurozone:ab,ti OR eec:ab,ti OR ec:ab,ti OR (schengen:ab,ti AND (area:ab,ti OR countr*:ab,ti OR region*:ab,ti OR state:ab,ti OR states:ab,ti)) OR euroregion:ab,ti OR euroregions:ab,ti OR 'eastern europe'/de OR 'western europe'/de OR 'balkan peninsula'/exp OR balkan:ab,ti OR balkans:ab,ti OR 'baltic states'/de OR baltic:ab,ti OR 'southern europe'/de OR (mediterranean:ab,ti AND (area:ab,ti OR countr*:ab,ti OR region*:ab,ti OR state:ab,ti OR states:ab,ti)) OR (alpine:ab,ti AND (area:ab,ti OR countr*:ab,ti OR region*:ab,ti OR state:ab,ti OR states:ab,ti)) OR 'scandinavia'/de OR scandinavia:ab,ti OR scandinavian:ab,ti OR (nordic NEXT/1 (countr* OR state*)):ab,ti OR danubian:ab,ti OR 'iberian peninsula':ab,ti OR 'peninsula iberica':ab,ti OR 'péninsule ibérique':ab,ti OR 'iberiar penintsula':ab,ti OR iberia:ab,ti OR anatolia:ab,ti OR anadolu:ab,ti OR anatole:ab,ti OR anatolian:ab,ti OR 'yugoslavia (pre-1992)'/de OR 'yugoslavia'/exp OR yugoslavia:ab,ti OR 'czechoslovakia'/de OR czechoslovakia:ab,ti OR 'czecho slovakia':ab,ti OR ceskoslovensko:ab,ti OR 'cesko slovensko':ab,ti OR 'benelux'/exp OR benelux:ab,ti OR fennoscandia:ab,ti OR 'fenno scandinavia':ab,ti OR fennoskandi*:ab,ti OR (visegrád:ab,ti AND (group:ab,ti OR four:ab,ti OR triangle:ab,ti)) OR 'visegrádská čtyřka':ab,ti OR 'visegrádská skupina':ab,ti OR 'visegrádi együttműködés':ab,ti OR 'visegrádi négyek':ab,ti OR 'grupa wyszehradzka':ab,ti OR 'vyšehradská skupina':ab,ti OR 'vyšehradská štvorka':ab,ti OR 'European'/de OR 'EU citizen'/de OR 'Central European'/de OR 'Eastern European'/de OR 'Northern European'/de OR 'Southern European'/de OR 'Western European'/de OR 'austrian'/exp OR 'austria'/exp OR austria*:ab,ti OR osterreich*:ab,ti OR oesterreich*:ab,ti OR ostosterreich:ab,ti OR ostoesterreich:ab,ti OR sudosterreich:ab,ti OR sudoesterreich:ab,ti OR westosterreich:ab,ti OR westoesterreich:ab,ti OR burgenland:ab,ti OR carinthia:ab,ti OR karnten:ab,ti OR kaernten:ab,ti OR niederosterreich:ab,ti OR niederoesterreich:ab,ti OR oberosterreich:ab,ti OR oberoesterreich:ab,ti OR salzburg:ab,ti OR saizburg:ab,ti OR styria:ab,ti OR steiermark:ab,ti OR tyrol:ab,ti OR tirol:ab,ti OR vorarlberg:ab,ti OR vienna:ab,ti OR wien:ab,ti OR graz:ab,ti OR linz:ab,ti OR innsbruck:ab,ti OR klagenfurt:ab,ti OR villach:ab,ti OR wels:ab,ti OR 'st polten':ab,ti OR 'st poelten':ab,ti OR 'sankt polten':ab,ti OR 'sankt poelten':ab,ti OR dornbirn:ab,ti OR 'Belgium'/exp OR 'Belgian'/exp OR Belgi*:ti,ab OR Belge*:ti,ab OR Belg:ti,ab OR Brussel*:ti,ab OR Bruxelles:ti,ab OR Bruxelloise:ti,ab OR Walloon*:ti,ab OR Wallon*:ti,ab OR Vlaams:ti,ab OR Flander*:ti,ab OR Flandern:ti,ab OR Flandre:ti,ab OR Flemish:ti,ab OR Flamand:ti,ab OR Flemisch:ti,ab OR Flämisch*:ti,ab OR Vlaanderen:ti,ab OR Flamande:ti,ab OR Waals:ti,ab OR Antwerp*:ti,ab OR Anvers:ti,ab OR Henegouwen:ti,ab OR Hennegau:ti,ab OR Hainault:ti,ab OR Hainaut:ti,ab OR Liege:ti,ab OR Luik:ti,ab OR Luttich:ti,ab OR Limbourg:ti,ab OR Limburg:ti,ab OR Namur:ti,ab OR Namen:ti,ab OR Ostflandern:ti,ab OR Westflandern:ti,ab OR Ghent:ti,ab OR Gent:ti,ab OR Gand:ti,ab OR Charleroi:ti,ab OR Bruges:ti,ab OR Brugge*:ti,ab OR Schaerbeek:ti,ab OR Schaarbeek:ti,ab OR Anderlecht:ti,ab OR Leuven:ti,ab OR Louvain:ti,ab OR 'bulgaria'/exp OR 'bulgarian (citizen)'/exp OR 'Bulgarian (people)'/exp OR bulgaria*:ti,ab OR balgariya:ab,ti OR balgarija:ab,ti OR blagoevgrad*:ab,ti OR 'croatia'/exp OR 'croatian (citizen)'/exp OR 'Croat (people)'/exp OR croat*:ab,ti OR hrvatsk*:ab,ti OR hrvati:ab,ti OR bjelovar:ab,ti OR 'bjelovarsko bilogorska':ab,ti OR 'brod posavina':ab,ti OR 'brodsko posavska':ab,ti OR 'dubrovnik neretva':ab,ti OR 'dubrovacko neretvanska':ab,ti OR zagreb:ab,ti OR zagrebacka:ab,ti OR istria:ab,ti OR istarska:ab,ti OR karlovacka:ab,ti OR karlovac:ab,ti OR 'koprivnicko krizevacka':ab,ti OR koprivnica:ab,ti OR krizevci:ab,ti OR 'krapina zagorje':ab,ti OR 'krapinsko zagorska':ab,ti OR 'lika senj':ab,ti OR 'licko senjska':ab,ti OR medimurska:ab,ti OR medimurje:ab,ti OR osijek:ab,ti OR baranja:ab,ti OR 'osjecko baranjska':ab,ti OR 'pozega slavonia':ab,ti OR 'pozesko slavonska':ab,ti OR 'primorje gorski kotar':ab,ti OR 'primorsko goranska':ab,ti OR 'sibensko kninska':ab,ti OR 'sibensko kninske':ab,ti OR sibenik:ab,ti OR knin:ab,ti OR sisak:ab,ti OR 'sisacko moslavacka':ab,ti OR moslavina:ab,ti OR 'splitsko dalmatinska':ab,ti OR split:ab,ti OR dalmatia:ab,ti OR varazdin:ab,ti OR varazdinska:ab,ti OR viroviticko:ab,ti OR podravska:ab,ti OR virovitica:ab,ti OR podravina:ab,ti OR 'vukovarsko srijemska':ab,ti OR vukovar:ab,ti OR srijem:ab,ti OR zadar:ab,ti OR zadarska:ab,ti OR rijeka:ab,ti OR 'velika gorica':ab,ti OR 'slavonski brod':ab,ti OR pula:ab,ti OR 'cyprus'/exp OR 'cypriot'/exp OR cyprus:ab,ti OR cypriot*:ab,ti OR kypros:ab,ti OR kibris*:ab,ti OR kypriaki:ab,ti OR kyprioi:ab,ti OR nicosia:ab,ti OR lefkosa:ab,ti OR lefkosia:ab,ti OR famagusta:ab,ti OR magusa:ab,ti OR ammochostos:ab,ti OR gazimagusa:ab,ti OR kyrenia:ab,ti OR girne:ab,ti OR keryneia:ab,ti OR larnaca:ab,ti OR larnaka:ab,ti OR iskele:ab,ti OR limassol:ab,ti OR lemesos:ab,ti OR limasol:ab,ti OR leymosun:ab,ti OR paphos:ab,ti OR pafos:ab,ti OR baf:ab,ti OR strovolos:ab,ti OR lakatamia:ab,ti OR lakadamya:ab,ti OR 'kato polemidia':ab,ti OR 'kato polemidhia':ab,ti OR aglandjia:ab,ti OR eglence:ab,ti OR aglantzia:ab,ti OR aradhippou:ab,ti OR aradippou:ab,ti OR engomi:ab,ti OR 'czech (citizen)'/exp OR 'czech republic'/exp OR 'Czech (people)'/exp OR czech*:ab,ti OR cesky:ab,ti OR ceska:ab,ti OR cech:ab,ti OR cestina:ab,ti OR prague:ab,ti OR praha:ab,ti OR prag:ab,ti OR stredoces*:ab,ti OR jihoces*:ab,ti OR bohemia:ab,ti OR bohemian:ab,ti OR plzen*:ab,ti OR pilsen:ab,ti OR karlovars*:ab,ti OR 'karlovy vary':ab,ti OR usteck*:ab,ti OR usti:ab,ti OR liberec*:ab,ti OR 'hradec kralove':ab,ti OR kralovehradec*:ab,ti OR pardubic*:ab,ti OR olomouc*:ab,ti OR olomoc:ab,ti OR holomoc:ab,ti OR moravskoslezs*:ab,ti OR jihomorav*:ab,ti OR moravia:ab,ti OR moravian:ab,ti OR morava:ab,ti OR vysocina:ab,ti OR zlin:ab,ti OR zlinsk*:ab,ti OR 'ceske budejovice':ab,ti OR budweis:ab,ti OR brno:ab,ti OR ostrava:ab,ti OR 'Denmark'/exp OR 'Danish citizen'/exp OR 'Dane (people)'/exp OR Denmark:ti,ab OR Danish*:ti,ab OR dane:ti,ab OR danes:ti,ab OR Danmark:ti,ab OR dansk*:ti,ab OR Hovedstaden:ti,ab OR Midtjylland:ti,ab OR Nordjylland:ti,ab OR Sjaelland:ti,ab OR Sealand:ti,ab OR 'Zealand region':ti,ab OR 'region Zealand':ti,ab OR Syddanmark:ti,ab OR Jutland:ti,ab OR Jylland:ti,ab OR Sonderjyllands:ti,ab OR Copenhagen:ti,ab OR Kobenhavn:ti,ab OR Arhus:ti,ab OR Aarhus:ti,ab OR Bornholm:ti,ab OR Frederiksberg:ti,ab OR Frederiksborg:ti,ab OR Ringkjobing:ti,ab OR Viborg:ti,ab OR Vejle:ti,ab OR Roskilde:ti,ab OR Storstrøm:ti,ab OR Vestsjaellands:ti,ab OR 'West Zealand':ti,ab OR Funen:ti,ab OR Ribe:ti,ab OR 'Kalaallit Nunaat':ti,ab OR Gronland:ti,ab OR Foroyar:ti,ab OR Faeroerne:ti,ab OR 'Faroe islands':ti,ab OR Aalborg:ti,ab OR Alborg:ti,ab OR Odense:ti,ab OR Esbjerg:ti,ab OR Gentofte:ti,ab OR Gladsaxe:ti,ab OR Randers:ti,ab OR Kolding:ti,ab OR 'Estonia'/exp OR 'Estonian (citizen)'/exp OR 'Estonian (people)'/exp OR Estonia*:ti,ab OR Eesti:ti,ab OR Eestlased:ti,ab OR Eestlane:ti,ab OR Harju:ti,ab OR Harjumaa:ti,ab OR Hiiu:ti,ab OR Hiiumaa:ti,ab OR 'Ida Viru':ti,ab OR 'Ida Virumaa':ti,ab OR Jarvamaa:ti,ab OR Jarva:ti,ab OR Jogevamaa:ti,ab OR Jogeva:ti,ab OR Laanemaa:ti,ab OR Laane:ti,ab OR 'Laane Virumaa':ti,ab OR Parnu:ti,ab OR Parnumaa:ti,ab OR Polva:ti,ab OR Polvamaa:ti,ab OR Rapla:ti,ab OR Raplamaa:ti,ab OR Saare:ti,ab OR Saaremaa:ti,ab OR Tartu:ti,ab OR Tartumaa:ti,ab OR Valga:ti,ab OR Valgamaa:ti,ab OR Viljandimaa:ti,ab OR Viljandi:ti,ab OR Voru:ti,ab OR Vorumaa:ti,ab OR Tallinn:ti,ab OR Narva:ti,ab OR 'Kohtla Jarve':ti,ab OR Rakvere:ti,ab OR Maardu:ti,ab OR Sillamae:ti,ab OR Kuressaare:ti,ab OR 'Finland'/exp OR 'Finn (citizen)'/exp OR 'Finn (people)'/exp OR Finland:ti,ab OR Finnish*:ti,ab OR Finn:ti,ab OR Finns:ti,ab OR Suomi:ti,ab Suomen:ti,ab OR Suomalaiset:ti,ab OR Aland:ti,ab OR Ahvenanmaa:ti,ab OR Uusimaa:ti,ab OR Nyland:ti,ab OR Karelia:ti,ab OR Karjala:ti,ab OR Karelen:ti,ab OR Ostrobothnia:ti,ab OR Pohjanmaa:ti,ab OR Osterbotten:ti,ab OR Savonia:ti,ab OR Savo:ti,ab OR Savolax:ti,ab OR Kainuu:ti,ab OR Kajanaland*:ti,ab OR 'Kanta Hame':ti,ab OR Tavastia:ti,ab OR Tavastland:ti,ab OR Kymenlaakso:ti,ab OR Kymmenedalen:ti,ab OR Lapland:ti,ab OR Lappi:ti,ab OR Lappland:ti,ab OR 'Paijat Hame':ti,ab OR Pirkanmaa:ti,ab OR Birkaland:ti,ab OR Satakunta:ti,ab OR Satakunda:ti,ab OR Helsinki:ti,ab OR Helsingfors:ti,ab OR Espoo:ti,ab OR Esbo:ti,ab OR Tampere:ti,ab OR Tammerfors:ti,ab OR Vantaa:ti,ab OR Vanda:ti,ab OR Oulu:ti,ab OR Uleaborg:ti,ab OR Turku:ti,ab OR Abo:ti,ab OR Jyvaskyla:ti,ab OR Kuopio:ti,ab OR Lahti:ti,ab OR Lahtis:ti,ab OR Kouvola:ti,ab OR 'France'/exp OR 'Frenchman'/exp OR France:ti,ab OR French*:ti,ab OR Francais*:ti,ab OR Alsace:ti,ab OR Elsass:ti,ab OR Aquitaine:ti,ab OR Aquitania:ti,ab OR Akitania:ti,ab OR Aguiéne:ti,ab OR Auvergne:ti,ab OR Auvèrnhe:ti,ab OR Auvèrnha:ti,ab OR Normandie:ti,ab OR Normandy:ti,ab OR Normaundie:ti,ab OR Bourgogne:ti,ab OR Burgundy:ti,ab OR Bregogne:ti,ab OR Borgoégne:ti,ab OR Borgogne:ti,ab OR Brittany:ti,ab OR Breizh:ti,ab OR Bertaèyn:ti,ab OR Bretagne:ti,ab OR 'Champagne Ardenne':ti,ab OR Corse:ti,ab OR Corsica:ti,ab OR 'Franche Comte':ti,ab OR 'Frantche Comte':ti,ab OR 'Franche Comtat':ti,ab OR Guadeloupe:ti,ab OR Guyane:ti,ab OR Guiana:ti,ab OR 'Languedoc Roussillon':ti,ab OR 'Lengadoc Rosselhon':ti,ab OR 'Llenguadoc-Rossello':ti,ab OR Limousin:ti,ab OR Lemosin:ti,ab OR Lorraine:ti,ab OR Lothringen:ti,ab OR Lottringe:ti,ab OR Martinique:ti,ab OR 'Midi Pyrenees':ti,ab OR 'Miègjorn Pirenèus':ti,ab OR 'Mieidia Pirenèus':ti,ab OR 'Mediodia Pirineos':ti,ab OR 'Pays de la Loire':ti,ab OR 'Broioù al Liger':ti,ab OR Picardie:ti,ab OR Picardy:ti,ab OR 'Poitou Charentes':ti,ab OR 'Peitau Charantas':ti,ab OR 'Poetou-Cherentes':ti,ab OR Provence:ti,ab OR Provenca:ti,ab OR

Prouvenco:ti,ab OR 'Cote d Azur':ti,ab OR 'Costo d Azur':ti,ab OR 'Costa d Azur':ti,ab OR Reunion:ti,ab OR 'Rhone Alpes':ti,ab OR 'Rono Arpes':ti,ab OR 'Rose Aups':ti,ab OR Ain:ti,ab OR Aisne:ti,ab OR Allier:ti,ab OR 'Alpes de Haute Provence':ti,ab OR 'Haute Alpes':ti,ab OR 'Alpes Maritimes':ti,ab OR Ardeche:ti,ab OR Ardennes:ti,ab OR Ariege:ti,ab OR Aube:ti,ab OR Aude:ti,ab OR Aveyron:ti,ab OR 'Bas Rhin':ti,ab OR 'Bouches du Rhone':ti,ab OR Calvados:ti,ab OR Cantal:ti,ab OR Charente:ti,ab OR Cher:ti,ab OR Correze:ti,ab OR 'Corse du Sud':ti,ab OR 'Cote d Or':ti,ab OR 'Cotes d Armor':ti,ab OR Creuse:ti,ab OR 'Deux Sevres':ti,ab OR Dordogne:ti,ab OR Doubs:ti,ab OR Drome:ti,ab OR Essonne:ti,ab OR Eure:ti,ab OR Finistere:ti,ab OR Gard:ti,ab OR Gers:ti,ab OR Gironde:ti,ab OR 'Haute Corse':ti,ab OR 'Haute Garonne':ti,ab OR 'Haute Marne':ti,ab OR 'Hautes Alpes':ti,ab OR 'Haute Saone':ti,ab OR 'Haute Savoie':ti,ab OR 'Hautes Pyrenees':ti,ab OR 'Haute Vienne':ti,ab OR 'Haut Rhin':ti,ab OR 'Hauts de Seine':ti,ab OR Herault:ti,ab OR 'Ile de France':ti,ab OR 'Ille et Vilaine':ti,ab OR Indre:ti,ab OR Isere:ti,ab OR Jura:ti,ab OR Landes:ti,ab OR Loire:ti,ab OR Loiret:ti,ab OR (Lot NEAR/3 (departement OR department)):ab,ti OR 'Lot et Garonne':ti,ab OR 'Loir et Cher':ti,ab OR Lozere:ti,ab OR Manche:ti,ab OR Marne:ti,ab OR Mayenne:ti,ab OR Mayotte:ti,ab OR 'Meurthe et Moselle':ti,ab OR Meuse:ti,ab OR Morbihan:ti,ab OR Moselle:ti,ab OR (Nord NEAR/3 (department OR departement)):ti,ab OR Nievre:ti,ab OR Oise:ti,ab OR Orne:ti,ab OR 'Pas de calais':ti,ab OR 'Noord-Nauw van Kales':ti,ab OR Paris:ti,ab OR 'Puy de dome':ti,ab OR 'Pyrenees Atlantiques':ti,ab OR 'Pyrenees Orientales':ti,ab OR Rhone:ti,ab OR Sarthe:ti,ab OR Savoie:ti,ab OR 'Seine et Marne':ti,ab OR 'Seine Maritime':ti,ab OR Somme:ti,ab OR Tarn:ti,ab OR 'Territoire de Belfort':ti,ab OR 'Val de Marne':ti,ab OR 'Val d Oise':ti,ab OR Var:ti,ab OR Vaucluse:ti,ab OR Vendee:ti,ab OR Vienne:ti,ab OR Vosges:ti,ab OR Yonne:ti,ab OR Yvelines:ti,ab OR Marseille:ti,ab OR Lyon:ti,ab OR Nice:ti,ab OR Nantes:ti,ab OR Strasbourg:ti,ab OR Montpellier:ti,ab OR Bordeaux:ti,ab OR Lille:ti,ab OR Toulouse:ti,ab OR 'Outre Mer':ti,ab OR 'Seine Saint Denis':ti,ab OR 'Germany'/de OR 'German (citizen)'/exp OR German*:ti,ab OR Deutsch*:ti,ab OR Bundesrepublik:ti,ab OR Westdeutschland:ti,ab OR Ostdeutschland:ti,ab OR Baden:ti,ab OR Wuerttemberg:ti,ab OR Wurttemberg:ti,ab OR Bayern:ti,ab OR Bavaria:ti,ab OR Berlin:ti,ab OR Brandenburg:ti,ab OR Bremen:ti,ab OR Oldenburg:ti,ab OR Mitteldeutschland:ti,ab OR Rhein:ti,ab OR Rhine:ti,ab OR Hannover:ti,ab OR Braunschweig:ti,ab OR Göttingen:ti,ab OR Goettingen:ti,ab OR Nurnberg:ti,ab OR Nuernberg:ti,ab OR Ruhr:ti,ab OR Koln:ti,ab OR koeln:ti,ab OR Bonn:ti,ab OR Hamburg:ti,ab OR Hessen:ti,ab OR Hesse:ti,ab OR Hessia:ti,ab OR Mecklenburg:ti,ab OR Vorpommern:ti,ab OR Pomerania:ti,ab OR Niedersachsen:ti,ab OR Neddersassen:ti,ab OR Saxony:ti,ab OR Niederbayern:ti,ab OR 'Northern Rhine':ti,ab OR 'North Rhine':ti,ab OR Westphalia:ti,ab OR Westfalen:ti,ab OR 'Rhineland Palatinate':ti,ab OR 'Rheinland Pfalz':ti,ab OR Saarland:ti,ab OR Sachsen:ti,ab OR 'Schleswig Holstein':ti,ab OR Thuringia:ti,ab OR Thuringen:ti,ab OR Thueringen:ti,ab OR Munchen:ti,ab OR Muenchen:ti,ab OR Munich:ti,ab OR Frankfurt:ti,ab OR Stuttgart:ti,ab OR Dusseldorf:ti,ab OR Duesseldorf:ti,ab OR Dortmund:ti,ab OR Essen:ti,ab OR 'Greece'/exp OR 'Greek (citizen)'/exp OR 'Greek (people)'/exp OR Greece:ti,ab OR 'Hellenic republic':ti,ab OR Greek*:ti,ab OR Ellada:ti,ab OR Elladas:ti,ab OR 'Elliniki Dimokratia':ti,ab OR Hellas:ti,ab OR Hellenes:ti,ab OR Attica:ti,ab OR Attiki:ti,ab OR Makedonia*:ti,ab OR Macedonia:ti,ab OR Thraki:ti,ab OR Thrace:ti,ab OR Crete:ti,ab OR Kriti:ti,ab OR 'Ionia Nisia':ti,ab OR 'Ionion neson':ti,ab OR 'Ionion nIson':ti,ab OR 'Ionian islands':ti,ab OR 'Ionian island':ti,ab OR Epirus:ti,ab OR Ipeiros:ti,ab OR 'Periféreia Ipeírou':ti,ab OR 'North aegean':ti,ab OR 'Northern Aegean':ti,ab OR 'Aegean islands':ti,ab OR 'Aegean island':ti,ab OR 'Nisoi Agaiou':ti,ab OR 'Notio Aigaio':ti,ab OR Peloponnese:ti,ab OR Peloponniso*:ti,ab OR Thessaly:ti,ab OR Thessalia:ti,ab OR Thessalian:ti,ab OR Petthalia:ti,ab OR 'Voreio Aigaio':ti,ab OR 'Voreio Aigaiou':ti,ab OR 'South aegean':ti,ab OR 'Southern Aegean':ti,ab OR 'Mount athos':ti,ab OR 'Oros Athos':ti,ab OR Cyclades:ti,ab OR Cycklades:ti,ab OR Kiklades:ti,ab OR Dodecanese:ti,ab OR Dodekanisa:ti,ab OR Athens:ti,ab OR Athina:ti,ab OR Thessaloniki:ti,ab OR Thessalonica:ti,ab OR Patras:ti,ab OR Patra:ti,ab OR Pireas:ti,ab OR Piraeus:ti,ab OR Larissa:ti,ab OR Larisa:ti,ab OR Heraklion:ti,ab OR Heraclion:ti,ab OR Iraklion:ti,ab OR Irakleion:ti,ab OR Iraklio:ti,ab OR Volos:ti,ab OR Rhodes:ti,ab OR Rodos:ti,ab OR Ioannina:ti,ab OR Janina:ti,ab OR Yannena:ti,ab OR Chania:ti,ab OR Chalcis:ti,ab OR Chalkida:ti,ab OR 'Hungary'/exp OR 'Hungarian (citizen)'/exp OR 'Magyar (people)'/exp OR Hungar*:ti,ab OR Magyarorszag:ti,ab OR Magyar*:ti,ab OR Dunantuli:ti,ab OR Transdanubia:ti,ab OR Dunantul:ti,ab OR 'Great Plain':ti,ab OR 'Eszak Alfold':ti,ab OR 'Del Alfold':ti,ab OR 'Alfold es eszak':ti,ab OR 'Northern Alfold':ti,ab OR 'North Alfold':ti,ab OR 'South Alfold':ti,ab OR 'Southern Alfold':ti,ab OR Bacs:ti,ab OR Kiskun:ti,ab OR Baranya:ti,ab OR Bekes:ti,ab OR Borsod:ti,ab OR Abauj:ti,ab OR Zemplen:ti,ab OR Budapest:ti,ab OR Csongrad:ti,ab OR Fejer:ti,ab OR gyor:ti,ab OR moson:ti,ab OR sopron:ti,ab OR hajdu:ti,ab OR bihar:ti,ab OR Heves:ti,ab OR 'jasz nagykun szolnok':ti,ab OR komarom:ti,ab OR esztergom:ti,ab OR Nograd:ti,ab OR (Pest NEXT/3 (megye OR county)):ti,ab OR Somogy:ti,ab OR szabolcs:ti,ab OR szatmar:ti,ab OR bereg:ti,ab OR Tolna:ti,ab OR Vas:ti,ab OR Veszprem:ti,ab OR Zala:ti,ab OR Debrecen:ti,ab OR Miskolc:ti,ab OR Szeged:ti,ab OR Pecs:ti,ab OR Gyor:ti,ab OR Nyiregyhaza:ti,ab OR Kecskemet:ti,ab OR Szekesfehervar:ti,ab OR Szombathely:ti,ab OR 'Ireland'/exp OR 'Irish (citizen)'/exp OR Ireland:ti,ab OR Eire:ti,ab OR Irish*:ti,ab OR Fingal:ti,ab OR 'Fine Gall':ti,ab OR Dublin:ti,ab OR 'Ath Cliath':ti,ab OR 'Dun Laoghaire':ti,ab OR Wicklow:ti,ab OR 'Cill Mhantain':ti,ab OR 'Chill Mhantain':ti,ab OR Wexford:ti,ab OR 'Loch Garman':ti,ab OR Carlow:ti,ab OR Ceatharlach:ti,ab OR Kildare:ti,ab OR 'Cill Dara':ti,ab OR 'Chill Dara':ti,ab OR Meath:ti,ab OR 'An Mhi':ti,ab OR 'Contae na Mi':ti,ab OR Louth:ti,ab OR 'Contae Lu':ti,ab OR Monaghan:ti,ab OR Muineachán:ti,ab OR Mhuineacháin:ti,ab OR Cavan:ti,ab OR 'An Cabhan':ti,ab OR 'An Cabhain':ti,ab OR Longford:ti,ab OR 'An Longfort':ti,ab OR 'an Longfoirt':ti,ab OR Langfurd:ti,ab OR Westmeath:ti,ab OR 'An Iarmhi':ti,ab OR 'na Iarmhi':ti,ab OR Offaly:ti,ab OR 'Uibh Fhaili':ti,ab OR Laois:ti,ab OR Laoise:ti,ab OR Kilkenny:ti,ab OR 'Chill Chainnigh':ti,ab OR 'Cill Chainnigh':ti,ab OR Waterford:ti,ab OR 'Port Lairge':ti,ab OR Watterford:ti,ab OR Cork:ti,ab OR Corcaigh:ti,ab OR Chorcai:ti,ab OR Kerry:ti,ab OR Ciarrai:ti,ab OR Chiarrai:ti,ab OR Limerick:ti,ab OR Luimneach:ti,ab OR Luimnigh:ti,ab OR Tipperary:ti,ab OR 'Tiobraid Arann':ti,ab OR 'Thiobraid Arann':ti,ab OR Clare:ti,ab OR 'An Clar':ti,ab OR 'an Chlair':ti,ab OR Galway:ti,ab OR Gaillimh:ti,ab OR 'na Gaillimhe':ti,ab OR Mayo:ti,ab OR 'Maigh Eo':ti,ab OR 'Mhaigh Eo':ti,ab OR Roscommon:ti,ab OR 'Ros comain':ti,ab OR Sligo:ti,ab OR Sligeach:ti,ab OR Shligigh:ti,ab OR Leitrim:ti,ab OR Liatroim:ti,ab OR Liatroma:ti,ab OR Donegal:ti,ab OR 'Dhún na nGall':ti,ab OR Dinnygal:ti,ab OR Dunnyga:ti,ab OR Leinster:ti,ab OR Laighin:ti,ab OR 'Cúige Laighean':ti,ab OR Munster:ti,ab OR Mumhain:ti,ab OR 'Cúige Mumhan':ti,ab OR Connacht:ti,ab OR Connachta:ti,ab OR Drogheda:ti,ab OR 'Droichead Atha':ti,ab OR Dundalk:ti,ab OR 'Dún Dealgan':ti,ab OR Swords:ti,ab OR Sord:ti,ab OR Bray:ti,ab OR Bre:ti,ab OR Navan:ti,ab OR 'An Uaimh':ti,ab OR 'Italy'/exp OR 'Italian (citizen)'/exp OR 'Italic people'/exp OR Italy:ti,ab OR Italia*:ti,ab OR Abruzzo:ti,ab OR Abruzzi:ti,ab OR Basilicata:ti,ab OR Lucania:ti,ab OR Calabria:ti,ab OR Campania:ti,ab OR 'Emilia Romagna':ti,ab OR 'friuli venezia giulia':ti,ab OR Lazio:ti,ab OR Latium:ti,ab OR Liguria*:ti,ab OR Lombardy:ti,ab OR Lombardia:ti,ab OR Marche:ti,ab OR Marches:ti,ab OR Molisano:ti,ab OR Molise:ti,ab OR Piedmont*:ti,ab OR Piemonte:ti,ab OR Bolzano:ti,ab OR Bozen:ti,ab OR Trentino:ti,ab OR Trento:ti,ab OR Puglia:ti,ab OR Apulia:ti,ab OR Sardinia:ti,ab OR Sardegna:ti,ab OR Sicily:ti,ab OR Sicilia:ti,ab OR Toscana:ti,ab OR Tuscany:ti,ab OR Umbria:ti,ab OR 'Valle d Aosta':ti,ab OR 'Vallee d Aoste':ti,ab OR 'Aosta Valley':ti,ab OR Veneto:ti,ab OR Venetia:ti,ab OR Triveneto:ti,ab OR Rome:ti,ab OR Roma:ti,ab OR Milan:ti,ab OR Milano:ti,ab OR Naples:ti,ab OR Napoli:ti,ab OR Turin:ti,ab OR Torino:ti,ab OR Palermo:ti,ab OR Genoa:ti,ab OR Genova:ti,ab OR Bologna:ti,ab OR Florence:ti,ab OR Firenze:ti,ab OR Bari:ti,ab OR Catania:ti,ab OR 'Latvia'/exp OR 'Latvian (citizen)'/exp OR 'Lett (people)'/exp OR Latvi*:ti,ab OR Riga:ti,ab OR Courland:ti,ab OR Kurzeme:ti,ab OR Kurland:ti,ab OR Latgale:ti,ab OR Lettgallia:ti,ab OR Latgola:ti,ab OR Latgalia:ti,ab OR Vidzeme:ti,ab OR Vidumo:ti,ab OR Semigallia:ti,ab OR Semigalia:ti,ab OR Zemgale:ti,ab OR Pieriga:ti,ab OR Daugavpils:ti,ab OR Dinaburg:ti,ab OR Jekabpils:ti,ab OR Jakobstadt:ti,ab OR Jelgava:ti,ab OR Jurmala:ti,ab OR Liepaja:ti,ab OR Libau:ti,ab OR Rezekne:ti,ab OR Rezne:ti,ab OR Rositten:ti,ab OR Valmiera:ti,ab OR Wolmar:ti,ab OR Ventspils:ti,ab OR Windau:ti,ab OR Ogre:ti,ab OR 'Lithuania'/exp OR 'Lithuanian (citizen)'/exp OR Lithuania*:ti,ab OR 'Lietuvos Respublika':ti,ab OR Lietuva:ti,ab OR lietuviu:ti,ab OR Alytus:ti,ab OR Alytaus:ti,ab OR Kaunas:ti,ab OR Kauno:ti,ab OR Klaipeda:ti,ab OR Klaipedos:ti,ab OR Marijampoles:ti,ab OR Marijampole:ti,ab OR Panevezys:ti,ab OR Panevezio:ti,ab OR Siauliai:ti,ab OR Siauliu:ti,ab OR Taurages:ti,ab OR Taurage:ti,ab OR Telsiu:ti,ab OR Telsiai:ti,ab OR Utenos:ti,ab OR Utena:ti,ab OR Vilnius:ti,ab OR Vilniaus:ti,ab OR Mazeikiai:ti,ab OR Jonava:ti,ab OR Mazeikiu:ti,ab OR Jonavos:ti,ab OR 'Luxembourg'/exp OR Luxembourg*:ti,ab OR Luxemburg:ti,ab OR Letzebuerg:ti,ab OR Diekirch:ti,ab OR Grevenmacher:ti,ab OR 'Esch sur Alzette':ti,ab OR 'Esch Uelzecht':ti,ab OR 'Esch an der Alzette':ti,ab OR 'Esch an der Alzig':ti,ab OR Dudelange:ti,ab OR Diddeleng:ti,ab OR Düdelingen:ti,ab OR Duedelingen:ti,ab OR Schifflange:ti,ab OR Scheffleng:ti,ab OR Schifflingen:ti,ab OR Bettembourg:ti,ab OR Beetebuerg:ti,ab OR Bettemburg:ti,ab OR Petange:ti,ab OR Peiteng:ti,ab OR Petingen:ti,ab OR Ettelbruck:ti,ab OR Ettelbreck:ti,ab OR Ettelbrueck:ti,ab OR Diekirch:ti,ab OR Dikrech:ti,ab OR Strassen:ti,ab OR Stroossen:ti,ab OR Bertrange:ti,ab OR Bartreng:ti,ab OR Bartringen:ti,ab OR 'Malta'/exp OR 'Maltese (citizen)'/exp OR Malta:ti,ab OR Maltese*:ti,ab OR Maltin:ti,ab OR Gozo:ti,ab OR Ghawdex:ti,ab OR Valletta:ti,ab OR 'Ill Belt':ti,ab OR Birkirkara:ti,ab OR 'B Kara':ti,ab OR Birchircara:ti,ab OR Mosta:ti,ab OR Qormi:ti,ab OR 'St Paul s Bay':ti,ab OR 'Pawl il Bahar':ti,ab OR Zabbar:ti,ab OR Sliema:ti,ab OR Naxxar:ti,ab OR Gwann:ti,ab OR 'St John':ti,ab OR Zebbug:ti,ab OR 'Citta rohan':ti,ab OR Fgura:ti,ab OR 'Netherlands'/exp OR 'Dutchman'/exp OR Netherlands:ti,ab OR Nederland*:ti,ab OR Dutch*:ti,ab OR Drenthe:ti,ab OR Flevoland:ti,ab OR Friesland:ti,ab OR Fryslan:ti,ab OR Frisia:ti,ab OR Gelderland:ti,ab OR Guelders:ti,ab OR Groningen:ti,ab OR Limburg:ti,ab OR Brabant:ti,ab OR Holland:ti,ab OR Overijssel:ti,ab OR Overissel:ti,ab OR Utrecht:ti,ab OR Zeeland:ti,ab OR Amsterdam:ti,ab OR Rotterdam:ti,ab OR Hague:ti,ab OR 's-Gravenhage':ti,ab OR 'Den Haag':ti,ab OR Eindhoven:ti,ab OR Tilburg:ti,ab OR Almere:ti,ab OR Breda:ti,ab OR Nijmegen:ti,ab OR Nimeguen:ti,ab OR 'Poland'/exp OR 'Polish citizen'/exp OR 'Pole (people)'/exp OR Poland:ti,ab OR Polska:ti,ab OR Polish:ti,ab OR Pole:ti,ab OR Poles:ti,ab OR Polski:ti,ab OR Polak:ti,ab OR Polka:ti,ab OR Polacy:ti,ab OR Dolnoslaskie:ti,ab OR Silesia*:ti,ab OR Slask:ti,ab OR Pomorskie:ti,ab OR Pomerania*:ti,ab OR Kujawsko:ti,ab OR Kuyavian:ti,ab OR Lodzkie:ti,ab OR Lodz:ti,ab OR Lubelskie:ti,ab OR Lublin:ti,ab OR Lubuskie:ti,ab OR Lubusz:ti,ab OR Lubus:ti,ab OR Malopolskie:ti,ab OR Mazowieckie:ti,ab OR Mazowske:ti,ab OR Masovia:ti,ab OR Masovian:ti,ab OR Opolskie:ti,ab OR Opole:ti,ab OR Podkarpackie:ti,ab OR Subcarpathian*:ti,ab OR Podlaskie:ti,ab OR Podlachia:ti,ab OR Podlasie:ti,ab OR Slaskie:ti,ab OR Swietokrzyskie:ti,ab OR 'Varmia Mazuria':ti,ab OR 'Varmian Mazurian':ti,ab OR 'Varmia Masuria':ti,ab OR 'Varmian Masurian':ti,ab OR 'Warmia Mazury':ti,ab OR 'Warminsko Mazurskie':ti,ab OR 'Warmian Masurian':ti,ab OR Wielkopolskie:ti,ab OR Zachodniopomorskie:ti,ab OR Warsaw:ti,ab OR Warszawa:ti,ab OR Krakow:ti,ab OR

Cracow:ti,ab OR Wroclaw:ti,ab OR Poznan:ti,ab OR Gdansk:ti,ab OR Szczecin:ti,ab OR Bydgoszcz:ti,ab OR Katowice:ti,ab OR 'Portugal'/exp OR 'Portuguese (citizen)'/exp OR Portugal:ti,ab OR Portugues*:ti,ab OR Azores:ti,ab OR Acores:ti,ab OR Madeira:ti,ab OR Alentejo:ti,ab OR Algarve:ti,ab OR Lisboa:ti,ab OR Lisbon:ti,ab OR 'Alto Tras-os-Montes':ti,ab OR (Ave NEAR/3 (community OR intermunicipal OR comunidade)):ti,ab OR Mondego:ti,ab OR Vouga:ti,ab OR Beira:ti,ab OR Cavado:ti,ab OR Lafoes:ti,ab OR Douro:ti,ab OR Porto:ti,ab OR Oporto:ti,ab OR Tejo:ti,ab OR Minho:ti,ab OR Setubal:ti,ab OR Pinhal:ti,ab OR 'Serra da Estrela':ti,ab OR Tamega:ti,ab OR Leira:ti,ab OR Santarem:ti,ab OR Beja:ti,ab OR Faro:ti,ab OR Evora:ti,ab OR Portalegre:ti,ab OR 'Castelo Branco':ti,ab OR Guarda:ti,ab OR Cimbra:ti,ab OR Aveiro:ti,ab OR Viseu:ti,ab OR Braganca:ti,ab OR Braganza:ti,ab OR Braga:ti,ab OR 'Vila real':ti,ab OR 'Viana do Castelo':ti,ab OR Gaia:ti,ab OR Amadora:ti,ab OR Funchal:ti,ab OR Coimbra:ti,ab OR Almada:ti,ab OR (Agualva:ti,ab AND Cacem:ti,ab) OR 'Romania'/exp OR 'Romanian (citizen)'/exp OR Romania*:ti,ab OR Rumania*:ti,ab OR Roumania*:ti,ab OR Romani:ti,ab OR Rumani:ti,ab OR Alba:ti,ab OR Arad:ti,ab OR Arges:ti,ab OR Bacau:ti,ab OR Bihor:ti,ab OR 'Bistrita Nasaud':ti,ab OR Botosani:ti,ab OR Braila:ti,ab OR Brasov:ti,ab OR Kronstadt:ti,ab OR Brasso:ti,ab OR Brassovia:ti,ab OR Coron:ti,ab OR Bucharest:ti,ab OR Bucuresti:ti,ab OR Buzau:ti,ab OR Calarasi:ti,ab OR 'Caras-Severin':ti,ab OR Cluj:ti,ab OR Klausenburg:ti,ab OR Kolozsvar:ti,ab OR Constanta:ti,ab OR Tomis:ti,ab OR Konstantia:ti,ab OR Kostence:ti,ab OR Covasna:ti,ab OR Dambovita:ti,ab OR Dolj:ti,ab OR Galati:ti,ab OR Galatz:ti,ab OR Galac:ti,ab OR Kalas:ti,ab OR Giurgiu:ti,ab OR Gorj:ti,ab OR Harghita:ti,ab OR Hunedoara:ti,ab OR Ialomita:ti,ab OR Iasi:ti,ab OR Jassy:ti,ab OR Lassy:ti,ab OR Ilfov:ti,ab OR Maramures:ti,ab OR Mehedinti:ti,ab OR Mures:ti,ab OR Neamt:ti,ab OR (Olt:ti,ab AND (river:ti,ab OR county:ti,ab OR region:ti,ab OR judetul:ti,ab OR Raul:ti,ab)) OR Prahova:ti,ab OR Salaj:ti,ab OR 'Satu Mare':ti,ab OR Sibiu:ti,ab OR Suceava:ti,ab OR Teleorman:ti,ab OR Timis:ti,ab OR Tulcea:ti,ab OR Valcea:ti,ab OR Vilcea:ti,ab OR Vaslui:ti,ab OR Vrancea:ti,ab OR Timisoara:ti,ab OR Temeswar:ti,ab OR Temeschburg:ti,ab OR Temeschwar:ti,ab OR Temesvar:ti,ab OR Temisvar:ti,ab OR Timisvar:ti,ab OR Temesva:ti,ab OR Craiova:ti,ab OR Ploiesti:ti,ab OR Ploesti:ti,ab OR Oradea:ti,ab OR Varad:ti,ab OR Varat:ti,ab OR 'Slovakia'/exp OR 'Slovak (citizen)'/exp OR 'Slovak (people)'/exp OR Slovakia:ti,ab OR Slovensk*:ti,ab OR Slovak*:ti,ab OR Slovaci:ti,ab OR Slovenki:ti,ab OR Bratislav*:ti,ab OR Presporok:ti,ab OR Pressburg:ti,ab OR Preßburg:ti,ab OR Posonium:ti,ab OR Banskobystri*:ti,ab OR 'Banska Bystrica':ti,ab OR Neusohl:ti,ab OR Besztercebánya:ti,ab OR Kosic*:ti,ab OR Kaschau:ti,ab OR Kassa:ti,ab OR Nitrian*:ti,ab OR Nitra:ti,ab OR Neutra:ti,ab OR Nyitra:ti,ab OR Nyitria:ti,ab OR Trnav*:ti,ab OR Tyrnau:ti,ab OR Nagyszombat:ti,ab OR Tyrnavia:ti,ab OR Presov*:ti,ab OR Trencian*:ti,ab OR Trencin:ti,ab OR Trentschin:ti,ab OR Trencsén:ti,ab OR Zilina:ti,ab OR Sillein:ti,ab OR Zsolna:ti,ab OR Zylina:ti,ab OR (Martin:ti,ab AND (city:ti,ab OR Svaty:ti,ab)) OR Turócszentmárton:ti,ab OR Poprad:ti,ab OR Deutschendorf:ti,ab OR Zvolen:ti,ab OR 'Slovenia'/exp OR 'Slovenian (citizen)'/exp OR 'Slovene (people)'/exp OR Slovenia*:ti,ab OR Slovenija:ti,ab OR slovensk*:ti,ab OR Slovenci:ti,ab OR Slovene*:ti,ab OR Gorenjska:ti,ab OR Carniola:ti,ab OR Goriska:ti,ab OR Gorizia:ti,ab OR Jugovzhodna:ti,ab OR Koroska:ti,ab OR Carinthia:ti,ab OR 'Notranjsko kraska':ti,ab OR 'Obalno kraska':ti,ab OR 'Coastal karst':ti,ab OR Osrednjeslovenska:ti,ab OR Podravska:ti,ab OR Drava:ti,ab OR Pomurska:ti,ab OR Mura:ti,ab OR Savinjska:ti,ab OR Savinja:ti,ab OR Spodnjeposavska:ti,ab OR Zasavska:ti,ab OR 'Central Sava':ti,ab OR Posavska:ti,ab OR 'Lower Sava':ti,ab OR Ljubljana:ti,ab OR Laibach:ti,ab OR Lubiana:ti,ab OR Maribor:ti,ab OR 'Marburg an der Drau':ti,ab OR Kranj:ti,ab OR Carnium:ti,ab OR Creina:ti,ab OR Chreina:ti,ab OR Krainbur:ti,ab OR Koper:ti,ab OR Capodistria:ti,ab OR Kopar:ti,ab OR Celje:ti,ab OR 'Novo mesto':ti,ab OR Neustadtl:ti,ab OR Domzale:ti,ab OR Velenje:ti,ab OR Wollan:ti,ab OR Woellan:ti,ab OR 'Nova Gorica':ti,ab OR Kamnik:ti,ab OR 'Spain'/exp OR 'Spaniard'/exp OR 'Basque (people)'/exp OR Spain:ti,ab OR Espana:ti,ab OR Spanish:ti,ab OR Espanol*:ti,ab OR Spaniard*:ti,ab OR Andalucia:ti,ab OR Andalusia:ti,ab OR Aragon:ti,ab OR Arago:ti,ab OR Cantabria:ti,ab OR Canarias:ti,ab OR 'Canary Islands':ti,ab OR (Canaries:ti,ab AND island*:ti,ab) OR 'Castile and leon':ti,ab OR 'Castilla y Leon':ti,ab OR 'Castile La Mancha':ti,ab OR 'Castilla La Mancha':ti,ab OR Cataluna:ti,ab OR Catalonia:ti,ab OR Ceuta:ti,ab OR Madrid:ti,ab OR Melilla:ti,ab OR Navarra:ti,ab OR Navarre:ti,ab OR Valencia*:ti,ab OR Extremadura:ti,ab OR Galicia:ti,ab OR Balears:ti,ab OR 'Balearic Islands':ti,ab OR 'Balear Islands':ti,ab OR Baleares:ti,ab OR 'La Rioja':ti,ab OR 'Pais Vasco':ti,ab OR 'Basque Country':ti,ab OR 'Baske region':ti,ab OR Euskadi:ti,ab OR Asturias:ti,ab OR Murcia:ti,ab OR Coruna:ti,ab OR Alava:ti,ab OR Araba:ti,ab OR Albacete:ti,ab OR Alicante:ti,ab OR Alacant:ti,ab OR Almeria:ti,ab OR Avila:ti,ab OR Badajoz:ti,ab OR Badajos:ti,ab OR Barcelona:ti,ab OR Burgos:ti,ab OR Caceres:ti,ab OR Cadiz:ti,ab OR Castellon:ti,ab OR Castello:ti,ab OR 'Ciudad Real':ti,ab OR Cordoba:ti,ab OR Cuenca:ti,ab OR Eivissa:ti,ab OR Ibiza:ti,ab OR Formentera:ti,ab OR 'El Hierro':ti,ab OR Fuerteventura:ti,ab OR Galiza:ti,ab OR Girona:ti,ab OR Gerona:ti,ab OR 'Gran Canaria':ti,ab OR Granada:ti,ab OR Guadalajara:ti,ab OR Guipuzcoa:ti,ab OR Gipuzkoa:ti,ab OR Huelva:ti,ab OR Huesca:ti,ab OR Jaen:ti,ab OR 'La Gomera':ti,ab OR 'La Palma':ti,ab OR Lanzarote:ti,ab OR Leon:ti,ab OR Lleida:ti,ab OR Lerida:ti,ab OR Lugo:ti,ab OR Malaga:ti,ab OR Mallorca:ti,ab OR Majorca:ti,ab OR Menorca:ti,ab OR Minorca:ti,ab OR Murcia:ti,ab OR Ourense:ti,ab OR Orense:ti,ab OR Palencia:ti,ab OR Pontevedra:ti,ab OR Salamanca:ti,ab OR Segovia:ti,ab OR Sevilla:ti,ab OR Seville:ti,ab OR Soria:ti,ab OR Tarragona:ti,ab OR Tenerife:ti,ab OR Teruel:ti,ab OR Toledo:ti,ab OR Valladolid:ti,ab OR Vizcaya:ti,ab OR Biscay:ti,ab OR Zamora:ti,ab OR Zaragoza:ti,ab OR Saragossa:ti,ab OR 'Las Palmas':ti,ab OR Bilbao:ti,ab OR Bilbo:ti,ab OR 'Sweden'/exp OR 'Swedish citizen'/exp OR 'Swede (people)'/exp OR Sweden:ti,ab OR Sverige:ti,ab OR Swedish:ti,ab OR Svenska:ti,ab OR svenskar:ti,ab OR Swede:ti,ab OR Swedes:ti,ab OR Norrland:ti,ab OR Mellansverige:ti,ab OR Smaland:ti,ab OR Stockholm*:ti,ab OR Sydsverige:ti,ab OR Vastsverige:ti,ab OR Blekinge:ti,ab OR Dalarna:ti,ab OR Gavleborg*:ti,ab OR Gotland*:ti,ab OR Halland*:ti,ab OR Jamtland*:ti,ab OR Jonkoping*:ti,ab OR Kalmar:ti,ab OR Kronoberg*:ti,ab OR Norrbotten*:ti,ab OR Orebro:ti,ab OR Ostergotland*:ti,ab OR Skane:ti,ab OR Sodermanlands:ti,ab OR Uppsala:ti,ab OR Varmland*:ti,ab OR Vasterbotten*:ti,ab OR Vasternorrland*:ti,ab OR Vastmanland*:ti,ab OR vastergotland*:ti,ab OR Gotaland*:ti,ab OR Gothenburg:ti,ab OR Goteborg:ti,ab OR Malmo:ti,ab OR Vasteras:ti,ab OR Linkoping:ti,ab OR Helsingborg:ti,ab OR Halsingborg:ti,ab OR Norrkoping:ti,ab OR 'United Kingdom'/exp OR 'British citizen'/exp OR 'GB':ti,ab OR 'United kingdom':ti,ab OR 'UK':ti,ab OR Britain:ti,ab OR British:ti,ab OR England:ti,ab OR English:ti,ab OR Scotland:ti,ab OR Scottish:ti,ab OR Scots:ti,ab OR Wales:ti,ab OR Cymru:ti,ab OR Welsh:ti,ab OR 'North Ireland':ti,ab OR 'Northern Ireland':ti,ab OR Irish:ti,ab OR Avon:ti,ab OR Bedfordshire:ti,ab OR Berkshire:ti,ab OR Bristol:ti,ab OR Buckinghamshire:ti,ab OR Cambridgeshire:ti,ab OR 'Isle of Ely':ti,ab OR Cheshire:ti,ab OR Cleveland:ti,ab OR Cornwall:ti,ab OR Cumberland:ti,ab OR Cumbria:ti,ab OR Derbyshire:ti,ab OR Devon:ti,ab OR Dorset:ti,ab OR Durham:ti,ab OR Essex:ti,ab OR Gloucestershire:ti,ab OR Hampshire:ti,ab OR Southampton:ti,ab OR (Hereford:ti,ab AND Worcester:ti,ab) OR Hertfordshire:ti,ab OR Herefordshire:ti,ab OR Humberside:ti,ab OR Huntingdon:ti,ab OR Huntingdonshire:ti,ab OR 'Isle of Wight':ti,ab OR Kent:ti,ab OR Lancashire:ti,ab OR Leicestershire:ti,ab OR Lincolnshire:ti,ab OR London:ti,ab OR Manchester:ti,ab OR Merseyside:ti,ab OR Middlesex:ti,ab OR Norfolk:ti,ab OR Northamptonshire:ti,ab OR Northumberland:ti,ab OR Nottinghamshire:ti,ab OR Oxfordshire:ti,ab OR Peterborough:ti,ab OR Rutland:ti,ab OR Shropshire:ti,ab OR Salop:ti,ab OR Somerset:ti,ab OR Yorkshire:ti,ab OR Staffordshire:ti,ab OR Suffolk:ti,ab OR Surrey:ti,ab OR Sussex:ti,ab OR (Tyne:ti,ab AND Wear:ti,ab) OR Warwickshire:ti,ab OR Midlands:ti,ab OR Westmorland:ti,ab OR Wiltshire:ti,ab OR Worcestershire:ti,ab OR 'Isle of Man':ti,ab OR Jersey:ti,ab OR Guernsey:ti,ab OR 'Channel Islands':ti,ab OR Aberdeen:ti,ab OR Aberdeenshire:ti,ab OR Angus:ti,ab OR Forfarshire:ti,ab OR Argyll:ti,ab OR Ayrshire:ti,ab OR Banffshire:ti,ab OR Berwickshire:ti,ab OR Bute:ti,ab OR Caithness:ti,ab OR Clackmannanshire:ti,ab OR Cromartyshire:ti,ab OR Dumfriesshire:ti,ab OR Dunbartonshire:ti,ab OR Dumbarton:ti,ab OR Dundee:ti,ab OR Lothian:ti,ab OR Haddingtonshire:ti,ab OR Edinburgh:ti,ab OR Fife:ti,ab OR Glasgow:ti,ab OR Inverness-shire:ti,ab OR Kincardineshire:ti,ab OR Kinross-shire:ti,ab OR Kirkcudbrightshire:ti,ab OR Lanarkshire:ti,ab OR Midlothian:ti,ab OR Moray:ti,ab OR Elginshire:ti,ab OR Nairnshire:ti,ab OR Orkney:ti,ab OR Peeblesshire:ti,ab OR Perthshire:ti,ab OR Renfrewshire:ti,ab OR (Ross:ti,ab AND Cromarty:ti,ab) OR Ross-shire:ti,ab OR Roxburghshire:ti,ab OR Selkirkshire:ti,ab OR Shetland:ti,ab OR Zetland:ti,ab OR Stirlingshire:ti,ab OR Sutherland:ti,ab OR Linlithgowshire:ti,ab OR Wigtownshire:ti,ab OR Anglesey:ti,ab OR Brecknockshire:ti,ab OR Caernarfonshire:ti,ab OR Carmarthenshire:ti,ab OR Cardiganshire:ti,ab OR Ceredigion:ti,ab OR Clwyd:ti,ab OR Denbighshire:ti,ab OR Dyfed:ti,ab OR Flintshire:ti,ab OR Glamorgan:ti,ab OR Gwent:ti,ab OR Gwynedd:ti,ab OR Merionethshire:ti,ab OR Montgomeryshire:ti,ab OR Monmouthshire:ti,ab OR Pembrokeshire:ti,ab OR Powys:ti,ab OR Radnorshire:ti,ab OR Antrim:ti,ab OR Aontroim:ti,ab OR 'Contae Aontroma':ti,ab OR Anthrim:ti,ab OR Antrìm:ti,ab OR Entrim:ti,ab OR Armagh:ti,ab OR 'Ard Mhacha':ti,ab OR Airmagh:ti,ab OR Belfast:ti,ab OR (Down:ti,ab AND (district:ti,ab OR council:ti,ab OR County:ti,ab)) OR 'An Dún':ti,ab OR 'an Dúin':ti,ab OR Doon:ti,ab OR Doun:ti,ab OR Fermanagh:ti,ab OR 'Fear Manach':ti,ab OR 'Fhear Manach':ti,ab OR Fermanay:ti,ab OR Londonderry:ti,ab OR Doire:ti,ab OR Dhoire:ti,ab OR Lunnonderrie:ti,ab OR Derry:ti,ab OR Birmingham:ti,ab OR Leeds:ti,ab OR Sheffield:ti,ab OR Bradford:ti,ab OR Liverpool:ti,ab OR 'Iceland'/exp OR 'Icelander'/exp OR Iceland:ti,ab OR Icelandic*:ti,ab OR islenska*:ti,ab OR Icelander*:ti,ab OR islendinga*:ti,ab OR Islendigar:ti,ab OR Inslenska:ti,ab OR Reykjavík:ti,ab OR Reykjavíkurborg:ti,ab OR Hofudborgarsvaedi:ti,ab OR Sudurnes:ti,ab OR Vesturland:ti,ab OR Vestfirdir:ti,ab OR Westfjords:ti,ab OR Nordurland:ti,ab OR Austurland:ti,ab OR Sudurland:ti,ab OR Kopavogur:ti,ab OR Hafnarfjordur:ti,ab OR Akureyri:ti,ab OR Gardabaer:ti,ab OR Mosfellsbaer:ti,ab OR Keflavik:ti,ab OR Akranes:ti,ab OR Selfoss:ti,ab OR Seltjarnarnes:ti,ab OR 'Bosnia and Herzegovina'/exp OR 'Bosnian (citizen)'/exp OR 'Bosniak (people)'/exp OR Bosnia*:ti,ab OR Herzegov*:ti,ab OR Herzegonine:ti,ab OR Bosna:ti,ab OR Bosne:ti,ab OR Bosanski:ti,ab OR Bosanac:ti,ab OR Bosanci:ti,ab OR Srpska:ti,ab OR Brcko:ti,ab OR Posavski:ti,ab OR Posavina:ti,ab OR posavska:ti,ab OR Tuzlanski:ti,ab OR Tuzla:ti,ab OR Tuzlanska:ti,ab OR 'Zenickho dobojski':ti,ab OR 'Zenicko dobojska':ti,ab OR Zenica:ti,ab OR 'Bosansko Podrinjski':ti,ab OR 'Bosansko Podrinjska':ti,ab OR Srednjobosanski:ti,ab OR hercegovacko:ti,ab OR Zapadnohercegovacki:ti,ab OR Zapadnohercegovacka:ti,ab OR Sarajevo:ti,ab OR Sarajevska:ti,ab OR 'Kanton 10':ti,ab OR '10 kanton':ti,ab OR Hercegbosanska:ti,ab OR 'Unsko sanski':ti,ab OR 'Una Sana':ti,ab OR 'Banja Luka':ti,ab OR bijeljina:ti,ab OR Mostar:ti,ab OR Prijedor:ti,ab OR Cazin:ti,ab OR Doboj:ti,ab OR Zupanija:ti,ab OR 'Kosovo'/exp OR 'Kosovar'/exp OR Kosov*:ti,ab OR Ferizaj*:ti,ab OR Urosevac*:ti,ab OR Gjakov*:ti,ab OR Dakovic*:ti,ab OR Gjilan*:ti,ab OR Gnjilan*:ti,ab OR Mitrovic*:ti,ab OR Pejes:ti,ab OR Peja:ti,ab OR Peje:ti,ab OR Pecki:ti,ab OR Pec:ti,ab OR Pristin*:ti,ab OR Prishtin*:ti,ab OR Pristinski:ti,ab OR Prizrenit:ti,ab OR Prizrenski:ti,ab OR Prizen:ti,ab OR Prizren:ti,ab OR Prizeni:ti,ab OR Produjev*:ti,ab OR Vucitrn:ti,ab OR Vushtrri*:ti,ab OR 'Suva reka':ti,ab OR Suhareka:ti,ab OR

Besiana:ti,ab OR Metohija:ti,ab OR Dukagjini:ti,ab OR Dukagjinit:ti,ab OR 'Liechtenstein'/exp OR Liechtenstein:ti,ab OR Lienchtensteiner*:ti,ab OR Balzers:ti,ab OR Eschen:ti,ab OR Gamprin:ti,ab OR Mauren:ti,ab OR Planken:ti,ab OR Ruggell:ti,ab OR Schaan:ti,ab OR Schellenberg:ti,ab OR Triesen:ti,ab OR Triesenberg:ti,ab OR Vaduz:ti,ab OR 'Norway'/exp OR 'Norwegian (citizen)'/exp OR 'Norwegian (people)'/exp OR Norway:ti,ab OR Norwegian*:ti,ab OR Norge:ti,ab OR Noreg:ti,ab OR Norgga:ti,ab OR Akershus:ti,ab OR 'Aust Agder':ti,ab OR Buskerud:ti,ab OR Finnmark:ti,ab OR Hedmark:ti,ab OR Hordaland:ti,ab OR 'More og Romsdal':ti,ab OR 'More and Romsdal':ti,ab OR 'More Romsdal':ti,ab OR Nordland:ti,ab OR Trondelag:ti,ab OR Oppland:ti,ab OR Oslo:ti,ab OR Ostfold:ti,ab OR Rogaland:ti,ab OR 'Sogn og fjordane':ti,ab OR 'Sogn and fjordane':ti,ab OR 'sogn fjordane':ti,ab OR Telemark:ti,ab OR Troms:ti,ab OR Romsa:ti,ab OR Romssa:ti,ab OR 'Vest Agder':ti,ab OR Vestfold:ti,ab OR Bergen:ti,ab OR Stavanger:ti,ab OR Sandnes:ti,ab OR Trondheim:ti,ab OR Trondhjem:ti,ab OR Kaupangen:ti,ab OR Nidaros:ti,ab OR Drammen:ti,ab OR Fredrikstad:ti,ab OR Skien:ti,ab OR Tromso:ti,ab OR Sarpsborg:ti,ab OR europe*:ad OR europa*:ad OR eu:ad OR eea:ad OR efta:ad OR 'eu/eea':ad OR 'eu/efta':ad OR ecsc:ad OR euratom:ad OR eurozone:ad OR eec:ad OR ec:ad OR (schengen:ad AND (area:ad OR countr*:ad OR region*:ad OR state:ad OR states:ad)) OR euroregion:ad OR euroregions:ad OR balkan:ad OR balkans:ad OR baltic:ad OR (mediterranean:ad AND (area:ad OR countr*:ad OR region*:ad OR state:ad OR states:ad)) OR (alpine:ad AND (area:ad OR countr*:ad OR region*:ad OR state:ad OR states:ad)) OR scandinavia:ad OR scandinavian:ad OR (nordic NEXT/1 (countr* OR state*)):ad OR danubian:ad OR 'iberian peninsula':ad OR 'peninsula iberica':ad OR 'péninsule ibérique':ad OR 'iberiar penintsula':ad OR iberia:ad OR anatolia:ad OR anadolu:ad OR anatole:ad OR anatolian:ad OR yugoslavia:ad OR czechoslovakia:ad OR 'czecho slovakia':ad OR ceskoslovensko:ad OR 'cesko slovensko':ad OR benelux:ad OR fennoscandia:ad OR 'fenno scandinavia':ad OR fennoskandi*:ad OR (visegrád:ad AND (group:ad OR four:ad OR triangle:ad)) OR 'visegrádská čtyřka':ad OR 'visegrádská skupina':ad OR 'visegrádi együttműködés':ad OR 'visegrádi négyek':ad OR 'grupa wyszehradzka':ad OR 'vyšehradská skupina':ad OR 'vyšehradská štvorka':ad OR austria*:ad OR osterreich*:ad OR oesterreich*:ad OR ostosterreich:ad OR ostoesterreich:ad OR sudosterreich:ad OR sudoesterreich:ad OR westosterreich:ad OR westoesterreich:ad OR burgenland:ad OR carinthia:ad OR karnten:ad OR kaernten:ad OR niederosterreich:ad OR niederoesterreich:ad OR oberosterreich:ad OR oberoesterreich:ad OR salzburg:ad OR saizburg:ad OR styria:ad OR steiermark:ad OR tyrol:ad OR tirol:ad OR vorarlberg:ad OR vienna:ad OR wien:ad OR graz:ad OR linz:ad OR innsbruck:ad OR klagenfurt:ad OR villach:ad OR wels:ad OR 'st polten':ad OR 'st poelten':ad OR 'sankt polten':ad OR 'sankt poelten':ad OR dornbirn:ad OR Belgi*:ad OR Belge*:ad OR Belg:ad OR Brussel*:ad OR Bruxelles:ad OR Bruxelloise:ad OR Walloon*:ad OR Wallon*:ad OR Vlaams:ad OR Flander*:ad OR Flandern:ad OR Flandre:ad OR Flemish:ad OR Flamand:ad OR Flemisch:ad OR Flämisch*:ad OR Vlaanderen:ad OR Flamande:ad OR Waals:ad OR Antwerp*:ad OR Anvers:ad OR Henegouwen:ad OR Hennegau:ad OR Hainault:ad OR Hainaut:ad OR Liege:ad OR Luik:ad OR Luttich:ad OR Limbourg:ad OR Limburg:ad OR Namur:ad OR Namen:ad OR Ostflandern:ad OR Westflandern:ad OR Ghent:ad OR Gent:ad OR Gand:ad OR Charleroi:ad OR Bruges:ad OR Brugge*:ad OR Schaerbeek:ad OR Schaarbeek:ad OR Anderlecht:ad OR Leuven:ad OR Louvain:ad OR Bulgaria:ad OR balgariya:ad OR balgarija:ad OR blagoevgrad*:ad OR 'pirin macedonia':ad OR burgas:ad OR dobrich:ad OR gabrovo:ad OR haskovo:ad OR kardzhali:ad OR kurdzhali:ad OR kyustendil:ad OR lovech:ad OR lovec:ad OR montana:ad OR pazardzhik:ad OR pernik:ad OR pleven*:ad OR plovdiv:ad OR razgrad:ad OR rousse:ad OR ruse:ad OR rusenka:ad OR shumen:ad OR silistra:ad OR sliven:ad OR smolyan:ad OR sofia:ad OR sofyiska:ad OR sofiiska:ad OR 'stara zagora':ad OR targovishte:ad OR varna:ad OR 'veliko tarnovo':ad OR vidin:ad OR vratsa:ad OR vratza:ad OR yambol:ad OR croat*:ad OR hrvatsk*:ad OR hrvati:ad OR bjelovar:ad OR 'bjelovarsko bilogorska':ad OR 'brod posavina':ad OR 'brodsko posavska':ad OR 'dubrovnik neretva':ad OR 'dubrovacko neretvanska':ad OR zagreb:ad OR zagrebacka:ad OR istria:ad OR istarska:ad OR karlovacka:ad OR karlovac:ad OR 'koprivnicko krizevacka':ad OR koprivnica:ad OR krizevci:ad OR 'krapina zagorje':ad OR 'krapinsko zagorska':ad OR 'lika senj':ad OR 'licko senjska':ad OR medimurska:ad OR medimurje:ad OR osijek:ad OR baranja:ad OR 'osjecko baranjska':ad OR 'pozega slavonia':ad OR 'pozesko slavonska':ad OR 'primorje gorski kotar':ad OR 'primorsko goranska':ad OR 'sibensko kninska':ad OR 'sibensko kninske':ad OR sibenik:ad OR knin:ad OR sisak:ad OR 'sisacko moslavacka':ad OR moslavina:ad OR 'splitsko dalmatinska':ad OR split:ad OR dalmatia:ad OR varazdin:ad OR varazdinska:ad OR viroviticko:ad OR podravska:ad OR virovitica:ad OR podravina:ad OR 'vukovarsko srijemska':ad OR vukovar:ad OR srijem:ad OR zadar:ad OR zadarska:ad OR rijeka:ad OR 'velika gorica':ad OR 'slavonski brod':ad OR pula:ad OR cyprus:ad OR cypriot*:ad OR kypros:ad OR kibris*:ad OR kypriaki:ad OR kyprioi:ad OR nicosia:ad OR lefkosa:ad OR lefkosia:ad OR famagusta:ad OR magusa:ad OR ammochostos:ad OR gazimagusa:ad OR kyrenia:ad OR girne:ad OR keryneia:ad OR larnaca:ad OR larnaka:ad OR iskele:ad OR limassol:ad OR lemesos:ad OR limasol:ad OR leymosun:ad OR paphos:ad OR pafos:ad OR baf:ad OR strovolos:ad OR lakatamia:ad OR lakadamya:ad OR 'kato polemidia':ad OR 'kato polemidhia':ad OR aglandjia:ad OR eglence:ad OR aglantzia:ad OR aradhippou:ad OR aradippou:ad OR engomi:ad OR czech*:ad OR cesky:ad OR ceska:ad OR cech:ad OR cestina:ad OR prague:ad OR praha:ad OR prag:ad OR stredoces*:ad OR jihoces*:ad OR bohemia:ad OR bohemian:ad OR plzen*:ad OR pilsen:ad OR karlovars*:ad OR 'karlovy vary':ad OR usteck*:ad OR usti:ad OR liberec*:ad OR 'hradec kralove':ad OR kralovehradec*:ad OR pardubic*:ad OR olomouc*:ad OR olomoc:ad OR holomoc:ad OR moravskoslezs*:ad OR jihomorav*:ad OR moravia:ad OR moravian:ad OR morava:ad OR vysocina:ad OR zlin:ad OR zlinsk*:ad OR 'ceske budejovice':ad OR budweis:ad OR brno:ad OR ostrava:ad OR Denmark:ad OR Danish*:ad OR dane:ad OR danes:ad OR Danmark:ad OR dansk*:ad OR Hovedstaden:ad OR Midtjylland:ad OR Nordjylland:ad OR Sjaelland:ad OR Sealand:ad OR 'Zealand region':ad OR 'region Zealand':ad OR Syddanmark:ad OR Jutland:ad OR Jylland:ad OR Sonderjyllands:ad OR Copenhagen:ad OR Kobenhavn:ad OR Arhus:ad OR Aarhus:ad OR Bornholm:ad OR Frederiksberg:ad OR Frederiksborg:ad OR Ringkjobing:ad OR Viborg:ad OR Vejle:ad OR Roskilde:ad OR Storstrøm:ad OR Vestsjaellands:ad OR 'West Zealand':ad OR Funen:ad OR Ribe:ad OR 'Kalaallit Nunaat':ad OR Gronland:ad OR Foroyar:ad OR Faeroerne:ad OR 'Faroe islands':ad OR Aalborg:ad OR Alborg:ad OR Odense:ad OR Esbjerg:ad OR Gentofte:ad OR Gladsaxe:ad OR Randers:ad OR Kolding:ad OR Estonia*:ad OR Eesti:ad OR Eestlased:ad OR Eestlane:ad OR Harju:ad OR Harjumaa:ad OR Hiiu:ad OR Hiiumaa:ad OR 'Ida Viru':ad OR 'Ida Virumaa':ad OR Jarvamaa:ad OR Jarva:ad OR Jogevamaa:ad OR Jogeva:ad OR Laanemaa:ad OR Laane:ad OR 'Laane Virumaa':ad OR Parnu:ad OR Parnumaa:ad OR Polva:ad OR Polvamaa:ad OR Rapla:ad OR Raplamaa:ad OR Saare:ad OR Saaremaa:ad OR Tartu:ad OR Tartumaa:ad OR Valga:ad OR Valgamaa:ad OR Viljandimaa:ad OR Viljandi:ad OR Voru:ad OR Vorumaa:ad OR Tallinn:ad OR Narva:ad OR 'Kohtla Jarve':ad OR Rakvere:ad OR Maardu:ad OR Sillamae:ad OR Kuressaare:ad OR Finland:ad OR Finnish*:ad OR Finn:ad OR Finns:ad OR Suomi:ad Suomen:ad OR Suomalaiset:ad OR Aland:ad OR Ahvenanmaa:ad OR Uusimaa:ad OR Nyland:ad OR Karelia:ad OR Karjala:ad OR Karelen:ad OR Ostrobothnia:ad OR Pohjanmaa:ad OR Osterbotten:ad OR Savonia:ad OR Savo:ad OR Savolax:ad OR Kainuu:ad OR Kajanaland*:ad OR 'Kanta Hame':ad OR Tavastia:ad OR Tavastland:ad OR Kymenlaakso:ad OR Kymmenedalen:ad OR Lapland:ad OR Lappi:ad OR Lappland:ad OR 'Paijat Hame':ad OR Pirkanmaa:ad OR Birkaland:ad OR Satakunta:ad OR Satakunda:ad OR Helsinki:ad OR Helsingfors:ad OR Espoo:ad OR Esbo:ad OR Tampere:ad OR Tammerfors:ad OR Vantaa:ad OR Vanda:ad OR Oulu:ad OR Uleaborg:ad OR Turku:ad OR Abo:ad OR Jyvaskyla:ad OR Kuopio:ad OR Lahti:ad OR Lahtis:ad OR Kouvola:ad OR France:ad OR French*:ad OR Francais*:ad OR Alsace:ad OR Elsass:ad OR Aquitaine:ad OR Aquitania:ad OR Akitania:ad OR Aguiéne:ad OR Auvergne:ad OR Auvèrnhe:ad OR Auvèrnha:ad OR Normandie:ad OR Normandy:ad OR Normaundie:ad OR Bourgogne:ad OR Burgundy:ad OR Bregogne:ad OR Borgoégne:ad OR Borgogne:ad OR Brittany:ad OR Breizh:ad OR Bertaèyn:ad OR Bretagne:ad OR 'Champagne Ardenne':ad OR Corse:ad OR Corsica:ad OR 'Franche Comte':ad OR 'Frantche Comte':ad OR 'Franche Comtat':ad OR Guadeloupe:ad OR Guyane:ad OR Guiana:ad OR 'Languedoc Roussillon':ad OR 'Lengadoc Rosselhon':ad OR 'Llenguadoc-Rossello':ad OR Limousin:ad OR Lemosin:ad OR Lorraine:ad OR Lothringen:ad OR Lottringe:ad OR Martinique:ad OR 'Midi Pyrenees':ad OR 'Miègjorn Pirenèus':ad OR 'Mieidia Pirenèus':ad OR 'Mediodia Pirineos':ad OR 'Pays de la Loire':ad OR 'Broioù al Liger':ad OR Picardie:ad OR Picardy:ad OR 'Poitou Charentes':ad OR 'Peitau Charantas':ad OR 'Poetou-Cherentes':ad OR Provence:ad OR Provenca:ad OR Prouvenco:ad OR 'Cote d Azur':ad OR 'Costo d Azur':ad OR 'Costa d Azur':ad OR Reunion:ad OR 'Rhone Alpes':ad OR 'Rono Arpes':ad OR 'Rose Aups':ad OR Ain:ad OR Aisne:ad OR Allier:ad OR 'Alpes de Haute Provence':ad OR 'Haute Alpes':ad OR 'Alpes Maritimes':ad OR Ardeche:ad OR Ardennes:ad OR Ariege:ad OR Aube:ad OR Aude:ad OR Aveyron:ad OR 'Bas Rhin':ad OR 'Bouches du Rhone':ad OR Calvados:ad OR Cantal:ad OR Charente:ad OR Cher:ad OR Correze:ad OR 'Corse du Sud':ad OR 'Cote d Or':ad OR 'Cotes d Armor':ad OR Creuse:ad OR 'Deux Sevres':ad OR Dordogne:ad OR Doubs:ad OR Drome:ad OR Essonne:ad OR Eure:ad OR Finistere:ad OR Gard:ad OR Gers:ad OR Gironde:ad OR 'Haute Corse':ad OR 'Haute Garonne':ad OR 'Haute Marne':ad OR 'Hautes Alpes':ad OR 'Haute Saone':ad OR 'Haute Savoie':ad OR 'Hautes Pyrenees':ad OR 'Haute Vienne':ad OR 'Haut Rhin':ad OR 'Hauts de Seine':ad OR Herault:ad OR 'Ile de France':ad OR 'Ille et Vilaine':ad OR Indre:ad OR Isere:ad OR Jura:ad OR Landes:ad OR Loire:ad OR Loiret:ad OR (Lot NEAR/3 (departement OR department)):ad OR 'Lot et Garonne':ad OR 'Loir et Cher':ad OR Lozere:ad OR Manche:ad OR Marne:ad OR Mayenne:ad OR Mayotte:ad OR 'Meurthe et Moselle':ad OR Meuse:ad OR Morbihan:ad OR Moselle:ad OR (Nord NEAR/3 (department OR departement)):ad OR Nievre:ad OR Oise:ad OR Orne:ad OR 'Pas de calais':ad OR 'Noord-Nauw van Kales':ad OR Paris:ad OR 'Puy de dome':ad OR 'Pyrenees Atlantiques':ad OR 'Pyrenees Orientales':ad OR Rhone:ad OR Sarthe:ad OR Savoie:ad OR 'Seine et Marne':ad OR 'Seine Maritime':ad OR Somme:ad OR Tarn:ad OR 'Territoire de Belfort':ad OR 'Val de Marne':ad OR 'Val d Oise':ad OR Var:ad OR Vaucluse:ad OR Vendee:ad OR Vienne:ad OR Vosges:ad OR Yonne:ad OR Yvelines:ad OR Marseille:ad OR Lyon:ad OR Nice:ad OR Nantes:ad OR Strasbourg:ad OR Montpellier:ad OR Bordeaux:ad OR Lille:ad OR Toulouse:ad OR 'Outre Mer':ad OR 'Seine Saint Denis':ad OR German*:ad OR Deutsch*:ad OR Bundesrepublik:ad OR Westdeutschland:ad OR Ostdeutschland:ad OR Baden:ad OR Wuerttemberg:ad OR Wurttemberg:ad OR Bayern:ad OR Bavaria:ad OR Berlin:ad OR Brandenburg:ad OR Bremen:ad OR Oldenburg:ad OR Mitteldeutschland:ad OR Rhein:ad OR Rhine:ad OR Hannover:ad OR Braunschweig:ad OR Göttingen:ad OR Goettingen:ad OR Nurnberg:ad OR Nuernberg:ad OR Ruhr:ad OR Koln:ad OR koeln:ad OR Bonn:ad OR Hamburg:ad OR Hessen:ad OR Hesse:ad OR Hessia:ad OR Mecklenburg:ad OR Vorpommern:ad OR Pomerania:ad OR Niedersachsen:ad OR Neddersassen:ad OR Saxony:ad OR Niederbayern:ad OR 'Northern Rhine':ad OR 'North Rhine':ad OR Westphalia:ad OR Westfalen:ad OR 'Rhineland Palatinate':ad OR 'Rheinland Pfalz':ad OR Saarland:ad OR Sachsen:ad OR 'Schleswig

Holstein':ad OR Thuringia:ad OR Thuringen:ad OR Thueringen:ad OR Munchen:ad OR Muenchen:ad OR Munich:ad OR Frankfurt:ad OR Stuttgart:ad OR Dusseldorf:ad OR Duesseldorf:ad OR Dortmund:ad OR Essen:ad OR Greece:ad OR 'Hellenic republic':ad OR Greek*:ad OR Ellada:ad OR Elladas:ad OR 'Elliniki Dimokratia':ad OR Hellas:ad OR Hellenes:ad OR Attica:ad OR Attiki:ad OR Makedonia*:ad OR Macedonia:ad OR Thraki:ad OR Thrace:ad OR Crete:ad OR Kriti:ad OR 'Ionia Nisia':ad OR 'Ionion neson':ad OR 'Ionion nIson':ad OR 'Ionian islands':ad OR 'Ionian island':ad OR Epirus:ad OR Ipeiros:ad OR 'Periféreia Ipeírou':ad OR 'North aegean':ad OR 'Northern Aegean':ad OR 'Aegean islands':ad OR 'Aegean island':ad OR 'Nisoi Agaiou':ad OR 'Notio Aigaio':ad OR Peloponnese:ad OR Peloponniso*:ad OR Thessaly:ad OR Thessalia:ad OR Thessalian:ad OR Petthalia:ad OR 'Voreio Aigaio':ad OR 'Voreio Aigaiou':ad OR 'South aegean':ad OR 'Southern Aegean':ad OR 'Mount athos':ad OR 'Oros Athos':ad OR Cyclades:ad OR Cycklades:ad OR Kiklades:ad OR Dodecanese:ad OR Dodekanisa:ad OR Athens:ad OR Athina:ad OR Thessaloniki:ad OR Thessalonica:ad OR Patras:ad OR Patra:ad OR Pireas:ad OR Piraeus:ad OR Larissa:ad OR Larisa:ad OR Heraklion:ad OR Heraclion:ad OR Iraklion:ad OR Irakleion:ad OR Iraklio:ad OR Volos:ad OR Rhodes:ad OR Rodos:ad OR Ioannina:ad OR Janina:ad OR Yannena:ad OR Chania:ad OR Chalcis:ad OR Chalkida:ad OR Hungar*:ad OR Magyarorszag:ad OR Magyar*:ad OR Dunantuli:ad OR Transdanubia:ad OR Dunantul:ad OR 'Great Plain':ad OR 'Eszak Alfold':ad OR 'Del Alfold':ad OR 'Alfold es eszak':ad OR 'Northern Alfold':ad OR 'North Alfold':ad OR 'South Alfold':ad OR 'Southern Alfold':ad OR Bacs:ad OR Kiskun:ad OR Baranya:ad OR Bekes:ad OR Borsod:ad OR Abauj:ad OR Zemplen:ad OR Budapest:ad OR Csongrad:ad OR Fejer:ad OR gyor:ad OR moson:ad OR sopron:ad OR hajdu:ad OR bihar:ad OR Heves:ad OR 'jasz nagykun szolnok':ad OR komarom:ad OR esztergom:ad OR Nograd:ad OR (Pest NEXT/3 (megye OR county)):ad OR Somogy:ad OR szabolcs:ad OR szatmar:ad OR bereg:ad OR Tolna:ad OR Vas:ad OR Veszprem:ad OR Zala:ad OR Debrecen:ad OR Miskolc:ad OR Szeged:ad OR Pecs:ad OR Gyor:ad OR Nyiregyhaza:ad OR Kecskemet:ad OR Szekesfehervar:ad OR Szombathely:ad OR Ireland:ad OR Eire:ad OR Irish*:ad OR Fingal:ad OR 'Fine Gall':ad OR Dublin:ad OR 'Ath Cliath':ad OR 'Dun Laoghaire':ad OR Wicklow:ad OR 'Cill Mhantain':ad OR 'Chill Mhantain':ad OR Wexford:ad OR 'Loch Garman':ad OR Carlow:ad OR Ceatharlach:ad OR Kildare:ad OR 'Cill Dara':ad OR 'Chill Dara':ad OR Meath:ad OR 'An Mhi':ad OR 'Contae na Mi':ad OR Louth:ad OR 'Contae Lu':ad OR Monaghan:ad OR Muineachán:ad OR Mhuineacháin:ad OR Cavan:ad OR 'An Cabhan':ad OR 'An Cabhain':ad OR Longford:ad OR 'An Longfort':ad OR 'an Longfoirt':ad OR Langfurd:ad OR Westmeath:ad OR 'An Iarmhi':ad OR 'na Iarmhi':ad OR Offaly:ad OR 'Uibh Fhaili':ad OR Laois:ad OR Laoise:ad OR Kilkenny:ad OR 'Chill Chainnigh':ad OR 'Cill Chainnigh':ad OR Waterford:ad OR 'Port Lairge':ad OR Watterford:ad OR Cork:ad OR Corcaigh:ad OR Chorcai:ad OR Kerry:ad OR Ciarrai:ad OR Chiarrai:ad OR Limerick:ad OR Luimneach:ad OR Luimnigh:ad OR Tipperary:ad OR 'Tiobraid Arann':ad OR 'Thiobraid Arann':ad OR Clare:ad OR 'An Clar':ad OR 'an Chlair':ad OR Galway:ad OR Gaillimh:ad OR 'na Gaillimhe':ad OR Mayo:ad OR 'Maigh Eo':ad OR 'Mhaigh Eo':ad OR Roscommon:ad OR 'Ros comain':ad OR Sligo:ad OR Sligeach:ad OR Shligigh:ad OR Leitrim:ad OR Liatroim:ad OR Liatroma:ad OR Donegal:ad OR 'Dhún na nGall':ad OR Dinnygal:ad OR Dunnyga:ad OR Leinster:ad OR Laighin:ad OR 'Cúige Laighean':ad OR Munster:ad OR Mumhain:ad OR 'Cúige Mumhan':ad OR Connacht:ad OR Connachta:ad OR Drogheda:ad OR 'Droichead Atha':ad OR Dundalk:ad OR 'Dún Dealgan':ad OR Swords:ad OR Sord:ad OR Bray:ad OR Bre:ad OR Navan:ad OR 'An Uaimh':ad OR Italy:ad OR Italia*:ad OR Abruzzo:ad OR Abruzzi:ad OR Basilicata:ad OR Lucania:ad OR Calabria:ad OR Campania:ad OR 'Emilia Romagna':ad OR 'friuli venezia giulia':ad OR Lazio:ad OR Latium:ad OR Liguria*:ad OR Lombardy:ad OR Lombardia:ad OR Marche:ad OR Marches:ad OR Molisano:ad OR Molise:ad OR Piedmont*:ad OR Piemonte:ad OR Bolzano:ad OR Bozen:ad OR Trentino:ad OR Trento:ad OR Puglia:ad OR Apulia:ad OR Sardinia:ad OR Sardegna:ad OR Sicily:ad OR Sicilia:ad OR Toscana:ad OR Tuscany:ad OR Umbria:ad OR 'Valle d Aosta':ad OR 'Vallee d Aoste':ad OR 'Aosta Valley':ad OR Veneto:ad OR Venetia:ad OR Triveneto:ad OR Rome:ad OR Roma:ad OR Milan:ad OR Milano:ad OR Naples:ad OR Napoli:ad OR Turin:ad OR Torino:ad OR Palermo:ad OR Genoa:ad OR Genova:ad OR Bologna:ad OR Florence:ad OR Firenze:ad OR Bari:ad OR Catania:ad OR Latvi*:ad OR Riga:ad OR Courland:ad OR Kurzeme:ad OR Kurland:ad OR Latgale:ad OR Lettgallia:ad OR Latgola:ad OR Latgalia:ad OR Vidzeme:ad OR Vidumo:ad OR Semigallia:ad OR Semigalia:ad OR Zemgale:ad OR Pieriga:ad OR Daugavpils:ad OR Dinaburg:ad OR Jekabpils:ad OR Jakobstadt:ad OR Jelgava:ad OR Jurmala:ad OR Liepaja:ad OR Libau:ad OR Rezekne:ad OR Rezne:ad OR Rositten:ad OR Valmiera:ad OR Wolmar:ad OR Ventspils:ad OR Windau:ad OR Ogre:ad OR Lithuania*:ad OR 'Lietuvos Respublika':ad OR Lietuva:ad OR lietuviu:ad OR Alytus:ad OR Alytaus:ad OR Kaunas:ad OR Kauno:ad OR Klaipeda:ad OR Klaipedos:ad OR Marijampoles:ad OR Marijampole:ad OR Panevezys:ad OR Panevezio:ad OR Siauliai:ad OR Siauliu:ad OR Taurages:ad OR Taurage:ad OR Telsiu:ad OR Telsiai:ad OR Utenos:ad OR Utena:ad OR Vilnius:ad OR Vilniaus:ad OR Mazeikiai:ad OR Jonava:ad OR Mazeikiu:ad OR Jonavos:ad OR Luxembourg*:ad OR Luxemburg:ad OR Letzebuerg:ad OR Diekirch:ad OR Grevenmacher:ad OR 'Esch sur Alzette':ad OR 'Esch Uelzecht':ad OR 'Esch an der Alzette':ad OR 'Esch an der Alzig':ad OR Dudelange:ad OR Diddeleng:ad OR Düdelingen:ad OR Duedelingen:ad OR Schifflange:ad OR Scheffleng:ad OR Schifflingen:ad OR Bettembourg:ad OR Beetebuerg:ad OR Bettemburg:ad OR Petange:ad OR Peiteng:ad OR Petingen:ad OR Ettelbruck:ad OR Ettelbreck:ad OR Ettelbrueck:ad OR Diekirch:ad OR Dikrech:ad OR Strassen:ad OR Stroossen:ad OR Bertrange:ad OR Bartreng:ad OR Bartringen:ad OR Malta:ad OR Maltese*:ad OR Maltin:ad OR Gozo:ad OR Ghawdex:ad OR Valletta:ad OR 'Ill Belt':ad OR Birkirkara:ad OR 'B Kara':ad OR Birchircara:ad OR Mosta:ad OR Qormi:ad OR 'St Paul s Bay':ad OR 'Pawl il Bahar':ad OR Zabbar:ad OR Sliema:ad OR Naxxar:ad OR Gwann:ad OR 'St John':ad OR Zebbug:ad OR 'Citta rohan':ad OR Fgura:ad OR Netherlands:ad OR Nederland*:ad OR Dutch*:ad OR Drenthe:ad OR Flevoland:ad OR Friesland:ad OR Fryslan:ad OR Frisia:ad OR Gelderland:ad OR Guelders:ad OR Groningen:ad OR Limburg:ad OR Brabant:ad OR Holland:ad OR Overijssel:ad OR Overissel:ad OR Utrecht:ad OR Zeeland:ad OR Amsterdam:ad OR Rotterdam:ad OR Hague:ad OR 's-Gravenhage':ad OR 'Den Haag':ad OR Eindhoven:ad OR Tilburg:ad OR Almere:ad OR Breda:ad OR Nijmegen:ad OR Nimeguen:ad OR Poland:ad OR Polska:ad OR Polish:ad OR Pole:ad OR Poles:ad OR Polski:ad OR Polak:ad OR Polka:ad OR Polacy:ad OR Dolnoslaskie:ad OR Silesia*:ad OR Slask:ad OR Pomorskie:ad OR Pomerania*:ad OR Kujawsko:ad OR Kuyavian:ad OR Lodzkie:ad OR Lodz:ad OR Lubelskie:ad OR Lublin:ad OR Lubuskie:ad OR Lubusz:ad OR Lubus:ad OR Malopolskie:ad OR Mazowieckie:ad OR Mazowske:ad OR Masovia:ad OR Masovian:ad OR Opolskie:ad OR Opole:ad OR Podkarpackie:ad OR Subcarpathian*:ad OR Podlaskie:ad OR Podlachia:ad OR Podlasie:ad OR Slaskie:ad OR Swietokrzyskie:ad OR 'Varmia Mazuria':ad OR 'Varmian Mazurian':ad OR 'Varmia Masuria':ad OR 'Varmian Masurian':ad OR 'Warmia Mazury':ad OR 'Warminsko Mazurskie':ad OR 'Warmian Masurian':ad OR Wielkopolskie:ad OR Zachodniopomorskie:ad OR Warsaw:ad OR Warszawa:ad OR Krakow:ad OR Cracow:ad OR Wroclaw:ad OR Poznan:ad OR Gdansk:ad OR Szczecin:ad OR Bydgoszcz:ad OR Katowice:ad OR Portugal:ad OR Portugues*:ad OR Azores:ad OR Acores:ad OR Madeira:ad OR Alentejo:ad OR Algarve:ad OR Lisboa:ad OR Lisbon:ad OR 'Alto Tras-os-Montes':ad OR (Ave NEAR/3 (community OR intermunicipal OR comunidade)):ad OR Mondego:ad OR Vouga:ad OR Beira:ad OR Cavado:ad OR Lafoes:ad OR Douro:ad OR Porto:ad OR Oporto:ad OR Tejo:ad OR Minho:ad OR Setubal:ad OR Pinhal:ad OR 'Serra da Estrela':ad OR Tamega:ad OR Leira:ad OR Santarem:ad OR Beja:ad OR Faro:ad OR Evora:ad OR Portalegre:ad OR 'Castelo Branco':ad OR Guarda:ad OR Cimbra:ad OR Aveiro:ad OR Viseu:ad OR Braganca:ad OR Braganza:ad OR Braga:ad OR 'Vila real':ad OR 'Viana do Castelo':ad OR Gaia:ad OR Amadora:ad OR Funchal:ad OR Coimbra:ad OR Almada:ad OR (Agualva:ad AND Cacem:ad) OR Romania*:ad OR Rumania*:ad OR Roumania*:ad OR Romani:ad OR Rumani:ad OR Alba:ad OR Arad:ad OR Arges:ad OR Bacau:ad OR Bihor:ad OR 'Bistrita Nasaud':ad OR Botosani:ad OR Braila:ad OR Brasov:ad OR Kronstadt:ad OR Brasso:ad OR Brassovia:ad OR Coron:ad OR Bucharest:ad OR Bucuresti:ad OR Buzau:ad OR Calarasi:ad OR 'Caras-Severin':ad OR Cluj:ad OR Klausenburg:ad OR Kolozsvar:ad OR Constanta:ad OR Tomis:ad OR Konstantia:ad OR Kostence:ad OR Covasna:ad OR Dambovita:ad OR Dolj:ad OR Galati:ad OR Galatz:ad OR Galac:ad OR Kalas:ad OR Giurgiu:ad OR Gorj:ad OR Harghita:ad OR Hunedoara:ad OR Ialomita:ad OR Iasi:ad OR Jassy:ad OR Lassy:ad OR Ilfov:ad OR Maramures:ad OR Mehedinti:ad OR Mures:ad OR Neamt:ad OR (Olt:ad AND (river:ad OR county:ad OR region:ad OR judetul:ad OR Raul:ad)) OR Prahova:ad OR Salaj:ad OR 'Satu Mare':ad OR Sibiu:ad OR Suceava:ad OR Teleorman:ad OR Timis:ad OR Tulcea:ad OR Valcea:ad OR Vilcea:ad OR Vaslui:ad OR Vrancea:ad OR Timisoara:ad OR Temeswar:ad OR Temeschburg:ad OR Temeschwar:ad OR Temesvar:ad OR Temisvar:ad OR Timisvar:ad OR Temesva:ad OR Craiova:ad OR Ploiesti:ad OR Ploesti:ad OR Oradea:ad OR Varad:ad OR Varat:ad OR Slovakia:ad OR Slovensk*:ad OR Slovak*:ad OR Slovaci:ad OR Slovenki:ad OR Bratislav*:ad OR Presporok:ad OR Pressburg:ad OR Preßburg:ad OR Posonium:ad OR Banskobystri*:ad OR 'Banska Bystrica':ad OR Neusohl:ad OR Besztercebánya:ad OR Kosic*:ad OR Kaschau:ad OR Kassa:ad OR Nitrian*:ad OR Nitra:ad OR Neutra:ad OR Nyitra:ad OR Nyitria:ad OR Trnav*:ad OR Tyrnau:ad OR Nagyszombat:ad OR Tyrnavia:ad OR Presov*:ad OR Trencian*:ad OR Trencin:ad OR Trentschin:ad OR Trencsén:ad OR Zilina:ad OR Sillein:ad OR Zsolna:ad OR Zylina:ad OR (Martin:ad AND (city:ad OR Svaty:ad)) OR Turócszentmárton:ad OR Poprad:ad OR Deutschendorf:ad OR Zvolen:ad OR Slovenia*:ad OR Slovenija:ad OR slovensk*:ad OR Slovenci:ad OR Slovene*:ad OR Gorenjska:ad OR Carniola:ad OR Goriska:ad OR Gorizia:ad OR Jugovzhodna:ad OR Koroska:ad OR Carinthia:ad OR 'Notranjsko kraska':ad OR 'Obalno kraska':ad OR 'Coastal karst':ad OR Osrednjeslovenska:ad OR Podravska:ad OR Drava:ad OR Pomurska:ad OR Mura:ad OR Savinjska:ad OR Savinja:ad OR Spodnjeposavska:ad OR Zasavska:ad OR 'Central Sava':ad OR Posavska:ad OR 'Lower Sava':ad OR Ljubljana:ad OR Laibach:ad OR Lubiana:ad OR Maribor:ad OR 'Marburg an der Drau':ad OR Kranj:ad OR Carnium:ad OR Creina:ad OR Chreina:ad OR Krainbur:ad OR Koper:ad OR Capodistria:ad OR Kopar:ad OR Celje:ad OR 'Novo mesto':ad OR Neustadtl:ad OR Domzale:ad OR Velenje:ad OR Wollan:ad OR Woellan:ad OR 'Nova Gorica':ad OR Kamnik:ad OR Spain:ad OR Espana:ad OR Spanish:ad OR Espanol*:ad OR Spaniard*:ad OR Andalucia:ad OR Andalusia:ad OR Aragon:ad OR Arago:ad OR Cantabria:ad OR Canarias:ad OR 'Canary Islands':ad OR (Canaries:ad AND island*:ad) OR 'Castile and leon':ad OR 'Castilla y Leon':ad OR 'Castile La Mancha':ad OR 'Castilla La Mancha':ad OR Cataluna:ad OR Catalonia:ad OR Ceuta:ad OR Madrid:ad OR Melilla:ad OR Navarra:ad OR Navarre:ad OR Valencia*:ad OR Extremadura:ad OR Galicia:ad OR Balears:ad OR 'Balearic Islands':ad OR 'Balear Islands':ad OR Baleares:ad OR 'La Rioja':ad OR 'Pais Vasco':ad OR 'Basque Country':ad OR 'Baske region':ad OR Euskadi:ad OR Asturias:ad OR Murcia:ad OR Coruna:ad OR Alava:ad OR Araba:ad OR Albacete:ad OR Alicante:ad OR Alacant:ad OR Almeria:ad OR Avila:ad OR Badajoz:ad OR Badajos:ad OR Barcelona:ad OR Burgos:ad OR Caceres:ad OR Cadiz:ad OR Castellon:ad OR Castello:ad OR 'Ciudad Real':ad OR Cordoba:ad OR Cuenca:ad OR Eivissa:ad OR Ibiza:ad OR Formentera:ad OR 'El Hierro':ad OR Fuerteventura:ad OR

Galiza:ad OR Girona:ad OR Gerona:ad OR 'Gran Canaria':ad OR Granada:ad OR Guadalajara:ad OR Guipuzcoa:ad OR Gipuzkoa:ad OR Huelva:ad OR Huesca:ad OR Jaen:ad OR 'La Gomera':ad OR 'La Palma':ad OR Lanzarote:ad OR Leon:ad OR Lleida:ad OR Lerida:ad OR Lugo:ad OR Malaga:ad OR Mallorca:ad OR Majorca:ad OR Menorca:ad OR Minorca:ad OR Murcia:ad OR Ourense:ad OR Orense:ad OR Palencia:ad OR Pontevedra:ad OR Salamanca:ad OR Segovia:ad OR Sevilla:ad OR Seville:ad OR Soria:ad OR Tarragona:ad OR Tenerife:ad OR Teruel:ad OR Toledo:ad OR Valladolid:ad OR Vizcaya:ad OR Biscay:ad OR Zamora:ad OR Zaragoza:ad OR Saragossa:ad OR 'Las Palmas':ad OR Bilbao:ad OR Bilbo:ad OR Sweden:ad OR Sverige:ad OR Swedish:ad OR Svenska:ad OR svenskar:ad OR Swede:ad OR Swedes:ad OR Norrland:ad OR Mellansverige:ad OR Smaland:ad OR Stockholm*:ad OR Sydsverige:ad OR Vastsverige:ad OR Blekinge:ad OR Dalarna:ad OR Gavleborg*:ad OR Gotland*:ad OR Halland*:ad OR Jamtland*:ad OR Jonkoping*:ad OR Kalmar:ad OR Kronoberg*:ad OR Norrbotten*:ad OR Orebro:ad OR Ostergotland*:ad OR Skane:ad OR Sodermanlands:ad OR Uppsala:ad OR Varmland*:ad OR Vasterbotten*:ad OR Vasternorrland*:ad OR Vastmanland*:ad OR vastergotland*:ad OR Gotaland*:ad OR Gothenburg:ad OR Goteborg:ad OR Malmo:ad OR Vasteras:ad OR Linkoping:ad OR Helsingborg:ad OR Halsingborg:ad OR Norrkoping:ad OR 'GB':ad OR 'United kingdom':ad OR 'UK':ad OR Britain:ad OR British:ad OR England:ad OR English:ad OR Scotland:ad OR Scottish:ad OR Scots:ad OR Wales:ad OR Cymru:ad OR Welsh:ad OR 'North Ireland':ad OR 'Northern Ireland':ad OR Irish:ad OR Avon:ad OR Bedfordshire:ad OR Berkshire:ad OR Bristol:ad OR Buckinghamshire:ad OR Cambridgeshire:ad OR 'Isle of Ely':ad OR Cheshire:ad OR Cleveland:ad OR Cornwall:ad OR Cumberland:ad OR Cumbria:ad OR Derbyshire:ad OR Devon:ad OR Dorset:ad OR Durham:ad OR Essex:ad OR Gloucestershire:ad OR Hampshire:ad OR Southampton:ad OR (Hereford:ad AND Worcester:ad) OR Hertfordshire:ad OR Herefordshire:ad OR Humberside:ad OR Huntingdon:ad OR Huntingdonshire:ad OR 'Isle of Wight':ad OR Kent:ad OR Lancashire:ad OR Leicestershire:ad OR Lincolnshire:ad OR London:ad OR Manchester:ad OR Merseyside:ad OR Middlesex:ad OR Norfolk:ad OR Northamptonshire:ad OR Northumberland:ad OR Nottinghamshire:ad OR Oxfordshire:ad OR Peterborough:ad OR Rutland:ad OR Shropshire:ad OR Salop:ad OR Somerset:ad OR Yorkshire:ad OR Staffordshire:ad OR Suffolk:ad OR Surrey:ad OR Sussex:ad OR (Tyne:ad AND Wear:ad) OR Warwickshire:ad OR Midlands:ad OR Westmorland:ad OR Wiltshire:ad OR Worcestershire:ad OR 'Isle of Man':ad OR Jersey:ad OR Guernsey:ad OR 'Channel Islands':ad OR Aberdeen:ad OR Aberdeenshire:ad OR Angus:ad OR Forfarshire:ad OR Argyll:ad OR Ayrshire:ad OR Banffshire:ad OR Berwickshire:ad OR Bute:ad OR Caithness:ad OR Clackmannanshire:ad OR Cromartyshire:ad OR Dumfriesshire:ad OR Dunbartonshire:ad OR Dumbarton:ad OR Dundee:ad OR Lothian:ad OR Haddingtonshire:ad OR Edinburgh:ad OR Fife:ad OR Glasgow:ad OR Inverness-shire:ad OR Kincardineshire:ad OR Kinross-shire:ad OR Kirkcudbrightshire:ad OR Lanarkshire:ad OR Midlothian:ad OR Moray:ad OR Elginshire:ad OR Nairnshire:ad OR Orkney:ad OR Peeblesshire:ad OR Perthshire:ad OR Renfrewshire:ad OR (Ross:ad AND Cromarty:ad) OR Ross-shire:ad OR Roxburghshire:ad OR Selkirkshire:ad OR Shetland:ad OR Zetland:ad OR Stirlingshire:ad OR Sutherland:ad OR Linlithgowshire:ad OR Wigtownshire:ad OR Anglesey:ad OR Brecknockshire:ad OR Caernarfonshire:ad OR Carmarthenshire:ad OR Cardiganshire:ad OR Ceredigion:ad OR Clwyd:ad OR Denbighshire:ad OR Dyfed:ad OR Flintshire:ad OR Glamorgan:ad OR Gwent:ad OR Gwynedd:ad OR Merionethshire:ad OR Montgomeryshire:ad OR Monmouthshire:ad OR Pembrokeshire:ad OR Powys:ad OR Radnorshire:ad OR Antrim:ad OR Aontroim:ad OR 'Contae Aontroma':ad OR Anthrim:ad OR Antrìm:ad OR Entrim:ad OR Armagh:ad OR 'Ard Mhacha':ad OR Airmagh:ad OR Belfast:ad OR (Down:ad AND (district:ad OR council:ad OR County:ad)) OR 'An Dún':ad OR 'an Dúin':ad OR Doon:ad OR Doun:ad OR Fermanagh:ad OR 'Fear Manach':ad OR 'Fhear Manach':ad OR Fermanay:ad OR Londonderry:ad OR Doire:ad OR Dhoire:ad OR Lunnonderrie:ad OR Derry:ad OR Birmingham:ad OR Leeds:ad OR Sheffield:ad OR Bradford:ad OR Liverpool:ad OR Makedon*:ad OR Macedon*:ad OR Fyrom:ad OR Istocen:ad OR Severoistocen:ad OR Jugoistocen:ad OR Jugozapaden:ad OR Pelagonski:ad OR Pelagonia:ad OR Poloski:ad OR Polog:ad OR Skopski:ad OR Skopje:ad OR Ckonje:ad OR Vardar*:ad OR Bitola:ad OR Kumanovo:ad OR Prilep:ad OR Tetovo:ad OR Tetova:ad OR Tetove:ad OR Veles:ad OR Stip:ad OR Shtip:ad OR Ohrid:ad OR Gostivar:ad OR Gostivari:ad OR Strumica:ad OR Iceland:ad OR Icelandic*:ad OR islenska*:ad OR Icelander*:ad OR islendinga*:ad OR Islendigar:ad OR Inslenska:ad OR Reykjavík:ad OR Reykjavíkurborg:ad OR Hofudborgarsvaedi:ad OR Sudurnes:ad OR Vesturland:ad OR Vestfirdir:ad OR Westfjords:ad OR Nordurland:ad OR Austurland:ad OR Sudurland:ad OR Kopavogur:ad OR Hafnarfjordur:ad OR Akureyri:ad OR Gardabaer:ad OR Mosfellsbaer:ad OR Keflavik:ad OR Akranes:ad OR Selfoss:ad OR Seltjarnarnes:ad OR Bosnia*:ad OR Herzegov*:ad OR Herzegonine:ad OR Bosna:ad OR Bosne:ad OR Bosanski:ad OR Bosanac:ad OR Bosanci:ad OR Srpska:ad OR Brcko:ad OR Posavski:ad OR Posavina:ad OR posavska:ad OR Tuzlanski:ad OR Tuzla:ad OR Tuzlanska:ad OR 'Zenickho dobojski':ad OR 'Zenicko dobojska':ad OR Zenica:ad OR 'Bosansko Podrinjski':ad OR 'Bosansko Podrinjska':ad OR Srednjobosanski:ad OR hercegovacko:ad OR Zapadnohercegovacki:ad OR Zapadnohercegovacka:ad OR Sarajevo:ad OR Sarajevska:ad OR 'Kanton 10':ad OR '10 kanton':ad OR Hercegbosanska:ad OR 'Unsko sanski':ad OR 'Una Sana':ad OR 'Banja Luka':ad OR bijeljina:ad OR Mostar:ad OR Prijedor:ad OR Cazin:ad OR Doboj:ad OR Zupanija:ad OR Kosov*:ad OR Ferizaj*:ad OR Urosevac*:ad OR Gjakov*:ad OR Dakovic*:ad OR Gjilan*:ad OR Gnjilan*:ad OR Mitrovic*:ad OR Pejes:ad OR Peja:ad OR Peje:ad OR Pecki:ad OR Pec:ad OR Pristin*:ad OR Prishtin*:ad OR Pristinski:ad OR Prizrenit:ad OR Prizrenski:ad OR Prizen:ad OR Prizren:ad OR Prizeni:ad OR Produjev*:ad OR Vucitrn:ad OR Vushtrri*:ad OR 'Suva reka':ad OR Suhareka:ad OR Besiana:ad OR Metohija:ad OR Dukagjini:ad OR Dukagjinit:ad OR Liechtenstein:ad OR Lienchtensteiner*:ad OR Balzers:ad OR Eschen:ad OR Gamprin:ad OR Mauren:ad OR Planken:ad OR Ruggell:ad OR Schaan:ad OR Schellenberg:ad OR Triesen:ad OR Triesenberg:ad OR Vaduz:ad OR Norway:ad OR Norwegian*:ad OR Norge:ad OR Noreg:ad OR Norgga:ad OR Akershus:ad OR 'Aust Agder':ad OR Buskerud:ad OR Finnmark:ad OR Hedmark:ad OR Hordaland:ad OR 'More og Romsdal':ad OR 'More and Romsdal':ad OR 'More Romsdal':ad OR Nordland:ad OR Trondelag:ad OR Oppland:ad OR Oslo:ad OR Ostfold:ad OR Rogaland:ad OR 'Sogn og fjordane':ad OR 'Sogn and fjordane':ad OR 'sogn fjordane':ad OR Telemark:ad OR Troms:ad OR Romsa:ad OR Romssa:ad OR 'Vest Agder':ad OR Vestfold:ad OR Bergen:ad OR Stavanger:ad OR Sandnes:ad OR Trondheim:ad OR Trondhjem:ad OR Kaupangen:ad OR Nidaros:ad OR Drammen:ad OR Fredrikstad:ad OR Skien:ad OR Tromso:ad OR Sarpsborg:ad OR Gibraltar:ti,ab OR Gibraltar:ad OR Hebrid*:ti,ab OR Hebrid*:ad OR Svalbard*:ti,ab OR Svalbard*:ad

Data extraction

Table S1: Variables for data extraction

Variable	Description	Values
**Reference**		
Author	Surname of first author of the article	Surname
Year	Year of publication of the article	Year: yyyy
Journal	Journal of publication	Journal name
**Study characteristics**		
Virus	Virus for which prevalence/incidence data are reported	HCV<br>HBV<br>both
Country	Country for which the study report prevalence estimates	Country name
Study design	Design of reported study	Randomised controlled trial<br>Surveillance study<br>Non-randomised, prospective comparative study<br>Prospective observational study<br>Retrospective observational study<br>Cross-sectional study<br>Meta-analysis/Systematic review<br>Mathematical modelling study

		Other
Period of sampling	Month/s and year/s during which study sampling was conducted	Month/s and year/s
Setting	Narrative field for relevant details of setting	
Sampling approach	Description of sampling approach	Exhaustive (screening)<br>Random sampling<br>Convenience sampling<br>Respondent-driven<br>Mixed<br>Other<br>Not reported<br>Not applicable
HBV: specimen	Specimen type	Serum<br>Saliva<br>Dry blood spot<br>Saliva/dry blood spot<br>Not reported<br>Not applicable<br>Other*
HBV: testing method	Method of testing	EIA HBsAg<br>CLIA HBsAg<br>ECLIA HBsAg<br>RIA HBsAg<br>RDT HBsAg<br>HBsAg, not specified<br>NAT<br>EIA + NAT<br>CLIA + NAT<br>RDT + NAT<br>not specified HBsAg + NAT<br>Not reported<br>Other*
HBV: confirmation	Whether a confirmation test was performed and what type of test	Yes/No; test
HBV: acute or chronic	Authors specification of disease type that reported prevalence/incidence applies to	Acute<br>Chronic<br>Both<br>Not specified<br>Not applicable
HCV: specimen	Specimen type	Serum<br>Saliva<br>Dry blood spot<br>Saliva/dry blood spot<br>Not reported<br>Not applicable<br>Other*
HCV: testing method	Method of testing	EIA anti-HCV<br>CLIA anti-HCV<br>ECLIA anti-HCV<br>RDT anti-HCV<br>Particle agglutination anti-HCV<br>anti-HCV, not specified<br>NAT<br>anti-HCV, not specified or NAT<br>EIA anti-HCV or NAT<br>Various anti-HCV<br>Not reported<br>Other*
HCV: confirmation test	Whether a confirmation test (anti-HCV) was performed and what type of test	No<br>anti-HCV, not specified<br>RIBA<br>EIA anti-HCV<br>LIA<br>EIA + RIBA

		Various anti-HCV<br>Other*
HCV-RNA	Whether NAT was performed	Yes/no
HCV: acute or chronic	Authors specification of disease type that reported prevalence/incidence applies to	Acute<br>Chronic<br>Both<br>Not specified<br>Not applicable
**Study population**		
Group of interest	Population subgroup sampled in the study	PLHIV<br>People in prison
Study population description	Narrative field for any further relevant information on the study population for which the prevalence/incidence is reported	
Study population N	Size of the study population	Numerical
Sample description	Narrative field to describe the sample of all included participants who provided specimens for testing	
Sample N	Size of the sample	Numerical
Inclusion criteria	Narrative field to list any additional criteria for inclusion not covered in sample description	
Exclusion criteria	Narrative field to list any additional criteria for exclusion not covered in sample description	
Gender: % male	Percentage of the sample that were male	%
Age, mean	Mean age of the sample ± standard deviation (if reported	Numerical
Age, median	Median age of the sample with interquartile range (if reported)	Numerical
Age, range	Minimum and maximum age of study population	Numerical
**Results HBV**		
Prevalence HBV %	% HBsAg positive	%
Prevalence HBV 95% CI	95% CI of the HBV prevalence	Numerical
Incidence value HBV	Value reported for incidence of HBV	Numerical
Incidence unit HBV	Unit reported for incidence of HBV	cases per 100 person-years<br>% per year<br>12 year cumulative incidence (%)<br>%<br>per 10 years<br>% per 75 days<br>Other*
Incidence HBV 95% CI	95% CI of incidence	Numerical
OR HBV in group compared to general population	OR of HBV in study population compared to the general population	Numerical
OR HBV 95% CI	95% CI of OR	Numerical
RR HBV in group compared to general population	RR of HBV in study population compared to the general population	Numerical
RR HBV 95% CI	95% CI of RR	Numerical
Other outcome HBV	Narrative field for other relevant reported outcomes	
Comments HBV	Narrative field for comments	
**Results HCV**		
Prevalence anti-HCV %	% anti-HCV positive (unconfirmed)	%
Prevalence anti-HCV 95% CI	95% CI of the anti-HCV prevalence (unconfirmed)	Numerical
Prevalence confirmed anti-HCV %	% anti-HCV positive (confirmed)	%
Prevalence confirmed anti-HCV 95% CI	95% CI of the anti-HCV prevalence (unconfirmed)	Numerical
Prevalence HCV-RNA %	% HCV-RNA positive	%
Prevalence HCV-RNA 95% CI	95% CI of the HCV-RNA prevalence	Numerical
Incidence value HCV	Value reported for incidence of HCV	Numerical
Incidence unit HCV	Unit reported for incidence of HCV	cases per 100 person-years<br>% per year<br>12 year cumulative incidence (%)

		%<br>per 10 years<br>% per 75 days<br>Other*
Incidence HCV 95% CI	95% CI of incidence	Numerical
OR HCV in group compared to general population	OR of HCV in study population compared to the general population	Numerical
OR HCV 95% CI	95% CI of OR	Numerical
RR HCV in group compared to general population	RR of HBV in study population compared to the general population	Numerical
RR HCV 95% CI	95% CI of RR	Numerical
Other outcome HCV	Narrative field for other relevant reported outcomes	
Comments HCV	Narrative field for comments	
**General**		
Critical appraisal	Narrative field for aspects of quality assessment	Aspects in which the study diverged from the checklist in table 4.
General comments	Narrative field for relevant comments on the study or further interpretation of the data extraction and critical appraisal	

Quality assessment

Table S2: Quality assessment checklist

- The relevance and purpose of the research are clearly described
- The methods used are clearly described and appropriate for the purpose of the research
- The selection of the study population is adequate for the purpose of the research
- Data collection is adequate for the purpose of the research
- The theoretical background is clearly described
- Data are analysed in depth
- Results and conclusions are clearly described
- The study population is clearly described (including where appropriate case detection and case definition)
- The population is representative of the source population
- The denominator is chosen appropriately (e.g. in case of surveillance studies)

Detailed summary tables

Hepatitis B

Table S3: Prevalence of HBV among PLHIV

Reference	Country and study period	Study design	Sampling approach	Subgroup	Study population	Sample N	Prevalence HBV (%)	Critical appraisal	General comments
Alexiev 2016	Bulgaria 2010-2014	Cross-sectional study	Not reported	PLHIV	HIV-1 positive patients nationwide, diagnosed between 2010-2014	794	10.4	- little detail in methods section<br>- selection study population not clear<br>- not clear whether study population is representative	
Alexiev 2016	Bulgaria 2010-2014	Cross-sectional study	Not reported	MSM living with HIV	HIV-1 positive MSM nationwide, diagnosed between 2010-2014	287	8.4	- little detail in methods section<br>- selection study population not clear<br>- not clear whether study population is representative	
Alexiev 2016	Bulgaria 2010-2014	Cross-sectional study	Not reported	PLHIV in prison	HIV-1 positive prisoners nationwide, diagnosed between 2010-2014	59	16.9	- little detail in methods section<br>- selection study population not clear<br>- not clear whether study population is representative	
Alexiev 2016	Bulgaria 2010-2014	Cross-sectional study	Not reported	PWID living with HIV	HIV-1 positive PWIDs nationwide, diagnosed between 2010-2014	150	20.6	- little detail in methods section<br>- selection study population not clear<br>- not clear whether study population is representative	
Andersen 2014	Denmark<br>Jan 2011-Jan 2013	Prospective observational study	Exhaustive (screening)	PLHIV	HIV+ patients actively followed at the outpatient clinic of Aarhus University Hospital	574	3	- Not clear how representative the study population is for all HIV patients in Denmark	A low HBV vaccination rate exists in this population, however, no seroconversions were observed during the study period
Larsen 2008	France<br>2004	Cross-sectional study	Mixed	PLHIV	HIV infected patients from 167 randomly selected wards throughout France	1849	7	- Methods serology not reported<br>- The sample population may not be representative of the source population	Participation rate of wards was not as high as expected (60.3%) which may present a bias
Larsen 2008	France<br>2004	Cross-sectional study	Mixed	MSM living with HIV	HIV infected MSM from 167 randomly selected wards throughout France	558	9.2	- Methods serology not reported<br>- The sample population may not be representative of the source population	Participation rate of wards was not as high as expected (60.3%) which may present a bias
Larsen 2008	France<br>2004	Cross-sectional study	Mixed	PWID living with HIV	HIV infected Intravenous drug users from 167 randomly selected wards throughout France	348	7.5	- Methods serology not reported<br>- The sample population may not be representative of the source population	Participation rate of wards was not as high as expected (60.3%) which may present a bias
Larsen 2008	France<br>2004	Cross-sectional study	Mixed	Transfusion recipients/haemophiliacs living with HIV	HIV infected transfusion recipients/haemophiliacs from 167 randomly selected wards throughout France	50	5.9	- Methods serology not reported<br>- The sample population may not be representative of the source population	Participation rate of wards was not as high as expected (60.3%) which may present a bias
Jansen 2015	Germany 1996-2012	Prospective observational study	Not reported	MSM living with HIV	HIV-1 positive MSM from multiple centres nationwide	1838	1.7	- Selection of study population not clear	Almost half of the population was vaccinated against HBV
Elefsiniotis 2006	Greece<br>NR	Retrospective observational study	Not reported	PLHIV	HIV-infected patients followed up at the department of infectious diseases at Athens	737	12.1	- The selection of the study population is not described<br>- The methods are not clearly described<br>- The study population is unlikely to be	The time period in which the study was performed and when/how often markers were measured is not reported

					University hospital since 1995			representative of the population of HIV+ patients in Greece	
Elefsiniotis 2006	Greece NR	Retrospective observational study	Not reported	MSM living with HIV	HIV-infected homosexual men followed up at the department of Infectious diseases at Athens University hospital since 1995	453	17.2	- The selection of the study population is not described<br>- The methods are not clearly described<br>- The study population is unlikely to be representative of the population of HIV+ patients in Greece	The time period in which the study was performed and when/how often markers were measured is not reported
Cicconi 2007	Italy 1997	Prospective observational study	Not reported	PLHIV	HIV-1 positive persons naive to antiretroviral drugs, in the ICONA study in 69 treatment centres across Italy	5272	3.7	- Methods serology not reported<br>- The methods are not clearly described	Methods of the ICONA study (time period, sampling) were not described in detail.
Sanarico 2016	Italy Jan- Dec 2013	Cross-sectional study	Not reported	PLHIV in prison	HIV+ Prisoners in detention centres of 7 Italian towns	69	8.8	- Data collection is not adequately described<br>- It is not clear whether the study population is representative of the source population	Prisoners previously diagnosed with HIV with sample left over are included in the study, however the original sampling approach is not described
Monarca 2015	Italy July 2013	Cross-sectional study	Exhaustive (screening)	PLHIV in prison	HIV+ prisoners at 25 correctional institutions across Italy	338	6.8	- Methods serology not reported<br>- Limited information on selection of study population<br>- Representative of study population unclear<br>- Limited information on study population	The survey was conducted in almost a quarter of all Italian prisoners. The study was focused on HIV prevalence.
Zhang 2006	Netherlands 1998-2012	Prospective observational study	Not reported	PLHIV	HIV-1 patients in the ATHENA cohort	12800	5	- Methods serology not reported<br>- The methods are not clearly described	Methods of the ATHENA study (sampling, number and location of centres) were not described in detail
Ruta 2005	Romania NR	Prospective observational study	Not reported	PLHIV	HIV-infected adolescents living in Constanta county	161	43.4	- The methods are not clearly described<br>- The study population is not representative of all HIV+ patients in Romania	The study population (adolescents) are not representative for all HIV+ patients. Little detail is given on sampling and no study period is reported
Cachafeiro 2011	Spain 2004-2008	Prospective observational study	Not reported	PLHIV	HIV+ patients from the CoRIS cohort who are HAART naive	4419	5.8	- The methods are not clearly described	The sampling methods of CoRIS were not reported. Prevalence of HBV is based on results at first testing
González-García 2005	Spain May-Sept 2002	Cross-sectional study	Other	PLHIV	HIV+ patients attending 39 HIV centres throughout Spain	1260	4.9	- Methods serology not reported<br>- Study population may not be representative of HIV+ patients in Spain	Selection of participants was by attendance during the study period. Therefore, patients with more frequent visits could be overrepresented
Llenas-García 2012	Spain 1992-2009	Retrospective observational study	Exhaustive (screening)	Migrants living with HIV	HIV+ migrants attending a clinic in Madrid	371	5.4	- Methods serology not reported<br>-It is not clear whether the population is representative for HIV+ migrants in the whole of Spain	As the prevalence of HBV in migrants is largely dependent on their country of origin, this could differ widely per region, depending on the origin of migrants there
Cachafeiro 2011	Spain 2004-2008	Prospective observational study	Not reported	MSM living with HIV	HIV+ patients who use Intravenous drugs from the CoRIS cohort who are HAART naive	721	7.8	- The methods are not clearly described	The sampling methods of CoRIS were not reported. Prevalence of HBV is based on results at first testing
Cachafeiro 2011	Spain 2004-2008	Prospective observational study	Not reported	MSM living with HIV	HIV+ MSM from the CoRIS cohort who are HAART naive	1852	5.8	- The methods are not clearly described	The sampling methods of CoRIS were not reported. Prevalence of HBV is based on results at first testing

Price 2012	UK<br>1996-2009	Prospective observational study	Not reported	PLHIV	HIV+ patients attending treatment centres participating in UK CHIC	25973	5.1	- Methods serology not reported<br>- Limited description of study population<br>- No clear if study population is representive of the source population	

Table S4: Prevalence of HBV among people in prison

Reference	Country and study period	Study design	Sampling approach	Subgroup	Study population	Sample N	Prevalence HBV (%)	Critical appraisal	General comments
Alexiev 2016	Bulgaria 2010-2014	Cross-sectional study	Not reported	PLHIV in prison	HIV-1 positive prisoners nationwide, diagnosed between 2010-2014	59	16.9	- little detail in methods section<br>- selection study population not clear<br>- not clear whether study population is representative	
Jacomet 2015	France June 2010-Dec 2013	Prospective observational study	Exhaustive (screening)	People in prison	Prisoners of the Clermont-Ferrand and Riom prisons	357	0.6	- unclear whether study popualtion is representative	participation rate was a little over 50%
Treso 2011	Hungary June 2007-June 2009	Cross-sectional study	Exhaustive (screening)	People in prison who got a tattoo in prison	Prisoners who got a tattoo in prison in 20 Hungarian prisons	222	2.3	- No major comments	
Treso 2011	Hungary June 2007-June 2009	Cross-sectional study	Exhaustive (screening)	People in prison who have had unprotected sex	Prisoners who have had unprotected sex in 20 Hungarian prisons	927	1.4	- No major comments	
Treso 2011	Hungary June 2007-June 2009	Cross-sectional study	Exhaustive (screening)	People in prison with a tattoo	Prisoners with a tattoo 20 Hungarian prisons	993	1.4	- No major comments	
Treso 2011	Hungary June 2007-June 2009	Cross-sectional study	Exhaustive (screening)	PWID in prison	PWID Prisoners in 20 Hungarian prisons	209	1.4	- No major comments	
Sanarico 2016	Italy<br>Jan- Dec 2013	Cross-sectional study	Not reported	PLHIV in prison	HIV+ Prisoners in detention centres of 7 Italian towns	69	8.8	- Data collection is not adequately described<br>- It is not clear whether the study population is representative of the source population	Prisoners previously diagnosed with HIV with sample left over are included in the study, however the original sampling approach is not described
Monarca 2015	Italy<br>July 2013	Cross-sectional study	Exhaustive (screening)	PLHIV in prison	HIV+ prisoners at 25 correctional institutions across Italy	338	6.8	- Methods serology not reported<br>- Limited information on selection of study population<br>- Representative of study population unclear<br>- Limited information on study population	The survey was conducted in almost a quarter of all Italian prisoners. The study was focused on HIV prevalence.

Table S5: Incidence of HBV among PLHIV

Reference	Country and study period	Study design	Sampling approach	Subgroup	Study population	Sample N	Incidence HBV	Critical appraisal	General comments
Andersen 2014	Denmark<br>Jan 2011-Jan 2013	Prospective observational study	Exhaustive (screening)	PLHIV	HIV+ patients actively followed at the outpatient clinic of Aarhus University Hospital	574	0.01 cases per 100 person-years	- Not clear how representative the study population is for all HIV patients in Denmark	
Jansen 2015	Germany 1996-2012	Prospective observational study	Not reported	MSM living with HIV	HIV-1 positive MSM from multiple centres nationwide	1838	2.51 cases per 100 person-years	- Selection of study population not clear	

Cicconi 2008	Italy 1997	Prospective observational study	Not reported	PLHIV	HIV-1 positive persons naive to antiretroviral drugs, participating in the ICONA study in 69 treament centres across Italy	1037	1.22 cases per 100 person-years	- Methods serology not reported<br>- The methods are not clearly described	
Cicconi 2008	Italy 1997	Prospective observational study	Not reported	MSM living with HIV	HIV-1 positive MSM naive to antiretroviral drugs, participating in the ICONA study in 69 treament centres across Italy	273	1.73 cases per 100 person-years	- Methods serology not reported<br>- The methods are not clearly described	
Cicconi 2008	Italy 1997	Prospective observational study	Not reported	STI- infected PLHIV	HIV-1 positive persons naive to antiretroviral drugs, participating in the ICONA study in 69 treament centres across Italy, with STIs at enrolment	112	1.25 cases per 100 person-years	- Methods serology not reported<br>- The methods are not clearly described	
Heuft 2014	Netherlands 1983-2012	Retrospective observational study	Not reported	MSM living with HIV	HIV+ MSM in the OLVG hospital cohort of Amsterdam	381	1.1 cases per 100 person-years	- Methods serology not reported<br>- Selection of study population not clear	
Ruta 2005	Romania NR	Prospective observational study	Not reported	PLHIV	HIV-infected adolescents living in Constanta county	161	2.49 cases per 100 person-years	- The methods are not clearly described<br>- The study population is not representative of all HIV+ patients in Romania	
Price 2012	UK 1996-2009	Prospective observational study	Not reported	PLHIV	HIV+ patients attending treatment centres participating in UK CHIC	25973	1.7 cases per 100 person-years	- Methods serology not reported<br>- Limited description of study population<br>- No clear if study population is representive of the source population	

Hepatitis C

Table S6: Prevalence of HCV among PLHIV

Reference	Country and study period	Study design	Sampling approach	Subgroup	Study population	Sample N	Prevalence anti-HCV (%)	Prevalence HCV-RNA (%)	Critical appraisal	General comments
Alexiev 2016	Bulgaria 2010-2014	Cross-sectional study	Not reported	PLHIV	HIV-1 positive patients nationwide, diagnosed between 2010-2014	794	25.6	20	- little detail in methods section<br>- selection study population not clear<br>- not clear whether study population is representative	
Alexiev 2016	Bulgaria 2010-2014	Cross-sectional study	Not reported	MSM living with HIV	HIV-1 positive MSM nationwide, diagnosed between 2010-2014	287	3	1.5	- little detail in methods section<br>- selection study population not clear<br>- not clear whether study population is representative	
Alexiev 2016	Bulgaria 2010-2014	Cross-sectional study	Not reported	PLHIV in prison	HIV-1 positive prisoners nationwide, diagnosed between 2010-2014	59	82	60.1	- little detail in methods section<br>- selection study population not clear	

									- not clear whether study population is representative	
Alexiev 2016	Bulgaria 2010-2014	Cross-sectional study	Not reported	PWID living with HIV	HIV-1 positive PWIDs nationwide, diagnosed between 2010-2014	150	87.4	71.6	- little detail in methods section<br>- selection study population not clear<br>- not clear whether study population is representative	
Andersen 2014	Denmark Jan 2011-Jan 2013	Prospective observational study	Exhaustive (screening)	PLHIV	HIV+ patients actively followed at the outpatient clinic of Aarhus University Hospital	574	7	4	- Not clear how representative the study population is for all HIV patients in Denmark	
Andersen 2014	Denmark Jan 2011-Jan 2013	Prospective observational study	Exhaustive (screening)	MSM living with HIV	HIV+ MSM actively followed in the HIV outpatient clinic at Aarhus University Hospital	207	4		- Not clear how representative the study population is for all HIV patients in Denmark	
Ghosn 2006	France 1996-2005	Prospective observational study	Exhaustive (screening)	PLHIV	HIV patients from the multicentre prospective ANRS PRIMO cohort	402	5.7	2.5	- Original selection criteria study population not described<br>- unclear whether study population is representative	Less than 1% of this population are injecting drug users; most of them became HIV+ through the sexual route.
Larsen 2008	France 2004	Cross-sectional study	Mixed	PLHIV	HIV infected patients from 167 randomly selected wards throughout France	1849	24.3		- Methods serology not reported<br>- The sample population may not be representative of the source population	Participation rate of wards was not as high as expected (60.3%) which may present a bias
Larsen 2008	France 2004	Cross-sectional study	Mixed	MSM living with HIV	HIV infected MSM from 167 randomly selected wards throughout France	558	3.1		- Methods serology not reported<br>- The sample population may not be representative of the source population	Participation rate of wards was not as high as expected (60.3%) which may present a bias
Larsen 2008	France 2004	Cross-sectional study	Mixed	PWID living with HIV	HIV infected Intravenous drug users from 167 randomly selected wards throughout France	348	92.8		- Methods serology not reported<br>- The sample population may not be representative of the source population	Participation rate of wards was not as high as expected (60.3%) which may present a bias
Larsen 2008	France 2004	Cross-sectional study	Mixed	Transfusion recipients/haemophiliacs living with HIV	HIV infected transfusion recipients/haemophiliacs from 167 randomly selected wards throughout France	50	47.1		- Methods serology not reported<br>- The sample population may not be representative of the source population	Participation rate of wards was not as high as expected (60.3%) which may present a bias
Jansen 2015	Germany 1996-2012	Prospective observational study	Not reported	MSM living with HIV	HIV-1 positive MSM from multiple centres nationwide	1838	8.2	4	- Selection of study population not clear	HCV prevalence may be underestimated because of delayed antibody response to acute infection, but may be overestimated as positive samples were not confirmed with immunoblot.
Elefsiniotis 2006	Greece NR	Retrospective observational study	Not reported	PLHIV	HIV-infected patients followed up at the department of infectious diseases at Athens	737	8.2	8.2	- The selection of the study population is not described<br>- The methods are not clearly described<br>- The study population is	The time period in which the study was performed and when/how often markers were measured is not reported

					University hospital since 1995				unlikely to be representative of the population of HIV+ patients in Greece	
Elefsiniotis 2006	Greece<br>NR	Retrospective observational study	Not reported	MSM living with HIV	HIV-infected homosexual men followed up at the department of Infectious diseases at Athens University hospital since 1995	453	8.6	8.6	- The selection of the study population is not described<br>- The methods are not clearly described<br>- The study population is unlikely to be representative of the population of HIV+ patients in Greece	The time period in which the study was performed and when/how often markers were measured is not reported
Surah<br>2013	Ireland Feb-Mar 2011	Cross-sectional study	Exhaustive (screening)	PLHIV	Adult HIV+ patients attending specialist clinics in Dublin	111	26		- Methods serology not reported<br>-Data are not analysed in depth<br>- The study population is unlikely to be representative of the population of HIV+ patients in Ireland	HCV prevalence is not the focus of this study, therefore the methods and analysis of these data are not well described
Cicconi 2007	Italy<br>1997	Prospective observational study	Not reported	PLHIV	HIV-1 positive persons naive to antiretroviral drugs, in the ICONA study in 69 treatment centres across Italy	5272	40.7		- Methods serology not reported<br>- The methods are not clearly described	Methods of the ICONA study (time period, sampling) were not described in detail.<br>It was reported that HCV RNA was also measured in a sample of anti-HCV positive patients and only a small proportion were HCV-RNA negative
Sanarico 2016	Italy<br>Jan-Dec 2013	Cross-sectional study	Not reported	PLHIV in prison	HIV+ Prisoners in detention centres of 7 Italian towns	69	78.3	65.2	- Data collection is not adequately described<br>- It is not clear whether the study population is representative of the source population	- Prisoners previously diagnosed with HIV with sample left over are included in the study, however the original sampling approach is not described
Monarca 2015	Italy<br>July 2013	Cross-sectional study	Exhaustive (screening)	PLHIV in prison	HIV+ prisoners at 25 correctional institutions across Italy	338	55.9		- Methods serology not reported<br>- Limited information on selection of study population<br>- Representative of study population unclear<br>- Limited information on study population	The survey was conducted in almost a quarter of all Italian prisoners. The study was focused on HIV prevalence.
Zhang<br>2006	Netherlands<br>1998-2012	Prospective observational study	Not reported	PLHIV	HIV-1 patients in the ATHENA cohort	12800	3.7		- Methods serology not reported<br>- The methods are not clearly described	Methods of the ATHENA study (sampling, number and location of centres) were not described in detail
van Rooijen 2016	Netherlands<br>2007-2011	Prospective observational study	Exhaustive (screening)	MSM living with HIV	All eligible HIV+ MSM who visited the STI clinic of Amsterdam	869	6.4		- It is unlikely that the study population is representative of the source population	48.6% of HIV+ MSM who opted-out of anti-HCV testing reported being HCV-positive.
Urbanus 2014	Netherlands<br>2010	Cross-sectional study	Exhaustive (screening)	MSM living with HIV	HIV+ MSM visiting the STI clinic of Amsterdam	146	10.3	6.2	- Sample may not be representative of all HIV+ MSM in the Netherlands	Due to the anonymous nature of STI clinic visits, no correction was possible for multiple

										measurements per person in the analysis. In the article, the prevalence is reported for different years between 1995-2010; here, the lastest data were extracted only.
Heiligenberg 2012	Netherlands Oct 2007-June 2008	Retrospective observational study	Other	MSM living with HIV	HIV infected MSM attending outpatient clinics of two hospitals in Amsterdam and Rotterdam	649		4.7	- The population is not representative of the source population	Not all the patients vsiting the outpatient clinic were invited to participate in the study due to logistic restrictions. Patients who spontaneously reported STI symptoms were excluded and referred to the STI clinic because the aim was to include those patients who at present would not have been screened for STI.
van Rooijen 2016	Netherlands 2007-2011	Prospective observational study	Exhaustive (screening)	MSM sex workers living with HIV	All eligible HIV+ MSM who visited the STI clinic of Amsterdam, who engaged in commercial sex work in the last 6 months	23	13		- It is unlikely that the study population is representative of the source population	48.6% of HIV+ MSM who opted-out of anti-HCV testing reported being HCV-positive.
Urbanus 2014	Netherlands 2010	Cross-sectional study	Exhaustive (screening)	MSM with chlamydia living with HIV	HIV+ MSM visiting the STI clinic of Amsterdam, who tested positive for chlamydia	92	25		- Sample may not be representative of all HIV+ MSM in the Netherlands	Due to the anonymous nature of STI clinic visits, no correction was possible for multiple measurements per person in the analysis. In the article, the prevalence is reported for different years between 1995-2010; here, the latest data were extracted only.
Grzeszczuk 2015	Poland 2008-2013	Cross-sectional study	Not reported	PLHIV	Adult HIV-1 infected patients treated at a clinic in Bialystok	457	71.1		- The methods are not clearly described<br>- The study population is not representative of all HIV+ patients in Poland	
Grzeszczuk 2015	Poland 2008-2013	Cross-sectional study	Not reported	PLHIV in prison	Adult HIV-1 infected patients treated at a clinic in Bialystok, who have been imprisoned	107	93.5		- The methods are not clearly described<br>- Selection of study population not clear<br>- The study population is not representative of all HIV+ patients in Poland	
Grzeszczuk 2015	Poland 2008-2013	Cross-sectional study	Not reported	PWID living with HIV	Adult HIV-1 infected patients treated at a clinic in Bialystok who use Intravenous drugs	264	97.7		- The methods are not clearly described<br>- Selection of study population not clear<br>- The study population is not representative of all HIV+ patients in Poland	
Ruta 2005	Romania NR	Prospective observational study	Not reported	PLHIV	HIV-infected adolescents living in Constanta county	161	1.8		- The methods are not clearly described<br>- The study population is	The study population (adolescents) are not representative for all HIV+ patients. Little detail is given

									not representative of all HIV+ patients in Romania	on sampling and no study period is reported
Skamperle 2014	Slovenia 2013	Cross-sectional study	Exhaustive (screening)	PLHIV	All Individuals confirmed as HIV+ in Slovenia by the end of 2013	579	7.6	5.7	- Description of methodology, background and interpretation of results are limited	MSM accounted for the majority of HIV+ individuals in the study (63%). The article is an update of a previous publication, therefore few methodological details are given, and the theoretical background and interpretation of results are limited
Berenguer 2016	Spain<br>June-July 2015	Cross-sectional study	Random sampling	PLHIV	HIV-infected patients in active follow-up in 41 centres throughout Spain	1867	37.7	22.1	- Methods serology not reported	
Rivero-Juarez 2015	Spain<br>June and Dec 2014	Cross-sectional study	Exhaustive (screening)	PLHIV	HIV infected patients in 17 hospitals in Andalusia	13.506	20.5		- No major comments	
Cachafeiro 2011	Spain<br>2004-2008	Prospective observational study	Not reported	PLHIV	HIV+ patients from the CoRIS cohort who are HAART naive	4419	21.8		- The methods are not clearly described	The sampling methods of CoRIS were not reported. Prevalence of HCV is based on results at first testing
González-García 2005	Spain<br>May-Sept 2002	Cross-sectional study	Other	PLHIV	HIV+ patients attending 39 HIV centres throughout Spain	1260	61	54.2	- Methods serology not reported<br>- Study population may not be representative of HIV+ patients in Spain	Selection of participants was by attendance during the study period. Therefore, patients with more frequent visits could be overrepresented
Llenas-García 2012	Spain<br>1992-2009	Retrospective observational study	Exhaustive (screening)	Migrants living with HIV	HIV+ migrants attending a clinic in Madrid	371	7.7		- Methods serology not reported<br>-It is not clear whether the population is representative for HIV+ migrants in the whole of Spain	As the prevalence of HCV in migrants is largely dependent on their country of origin, this could differ widely per region, depending on the origin of migrants there
Berenguer 2016	Spain<br>June-July 2015	Cross-sectional study	Random sampling	MSM living with HIV	HIV-infected MSM in active follow-up in 41 centres throughout Spain	655	4.3	1.6	- Methods serology not reported	
Cachafeiro 2011	Spain<br>2004-2008	Prospective observational study	Not reported	MSM living with HIV	HIV+ patients who use Intravenous drugs from the CoRIS cohort who are HAART naive	721	89.5		- The methods are not clearly described	The sampling methods of CoRIS were not reported. Prevalence of HCV is based on results at first testing
Berenguer 2016	Spain<br>June-July 2015	Cross-sectional study	Random sampling	PWID living with HIV	HIV-infected patients in active follow-up in 41 centres throughout Spain, using Intravenous drugs	573	91.4	53.8	- Methods serology not reported	
Cachafeiro 2011	Spain<br>2004-2008	Prospective observational study	Not reported	MSM living with HIV	HIV+ MSM from the CoRIS cohort who are HAART naive	1852	3.5		- The methods are not clearly described	The sampling methods of CoRIS were not reported. Prevalence of HCV is based on results at first testing
Stenkvist 2014	Sweden Sept 2010	Cross-sectional study	Exhaustive (screening)	PLHIV	HIV+ adults living in Sweden	4765	14	11	- Methods serology not reported	The nearly universal (90%) anti-HCV testing minimizes the risk of selection bias. However, since female sex and HIV transmission routes other than PWID and MSM were overrepresented among untested patients, the

										prevalence found might be an overestimation.
Stenkvist 2014	Sweden Sept 2010	Cross-sectional study	Exhaustive (screening)	MSM living with HIV	HIV+ MSM living in Sweden	1620	3.7		- Methods serology not reported	The nearly universal (90%) anti-HCV testing minimizes the risk of selection bias. However, since female sex and HIV transmission routes other than PWID and MSM were overrepresented among untested patients, the prevalence found might be an overestimation.
Stenkvist 2014	Sweden Sept 2010	Cross-sectional study	Exhaustive (screening)	PWID living with HIV	HIV+ Intravenous drug users living in Sweden	410	98		- Methods serology not reported	The nearly universal (90%) anti-HCV testing minimizes the risk of selection bias. However, since female sex and HIV transmission routes other than PWID and MSM were overrepresented among untested patients, the prevalence found might be an overestimation.
Turner 2010	UK 1996-2007	Prospective observational study	Not reported	PLHIV	HIV+ adults attending treatment centres participating in UK CHIC	20365	8.9		- Methods serology not reported<br>- The methods are not clearly described	The methods of the UK CHIC study (sampling, location centres) were not described in detail
Scott 2010	UK Jan - June 2007	Cross-sectional study	Exhaustive (screening)	MSM living with HIV	HIV+ MSM attending one of three sexual health clinics in England	339	0.88	0.88	- Methods serology not reported<br>- Study population is unlikely to be representative of all HIV+ MSM in UK	Here the prevalence of undiagnosed HCV was reported. This is lower than the true prevalence of HCV infection in MSM since individuals known to have HCV were excluded
Turner 2010	UK 1996-2007	Prospective observational study	Not reported	MSM living with HIV	HIV+ MSM attending treatment centres participating in UK CHIC	12059	7.2		- Methods serology not reported<br>- The methods are not clearly described	The methods of the UK CHIC study (sampling, location centres) were not described in detail
Dougan 2007	UK Jan 2002-Dec 2003	Cross-sectional study	Convenience sampling	MSM living with HIV	HIV+ MSM in England and Wales	242	4.5		- Methods serology not reported<br>- Methods not clearly described<br>- The study population may not be representative of all HIV+ MSM in UK	Individuals may be incorrectly matched or individuals may not be matched if the information has not been recorded correctly or was missing, which might lead to underestimation of the results. It was not clear why testing was performed in individuals (as part of routine screening or on the basis of clinical symptoms), therfore sampling method was not clear and it is not clear whether the sample is representative. The exclusion of individuals with other potential exposure sources may also reduce representativeness.

Turner 2010	UK 1996-2007	Prospective observational study	Not reported	PWID living with HIV	HIV+ Intravenous drug users attending treatment centres participating in UK CHIC	622	83.7		- Methods serology not reported<br>- The methods are not clearly described	The methods of the UK CHIC study (sampling, location centres) were not described in detail
Balogun 2009	UK 1998, 1999, 2000, 2001	Cross-sectional study	Not reported	PWID living with HIV	HIV+ (ex)PWID who attended GUM clinics in England, Northern Ireland and Wales	201	38.3		- selection study population not clear<br>- study population not clearly described<br>- population not completely representative of source population (also ex-PWID included)	RNA positivity rates of anti-HCV positive samples are low for one of the study years, indicating a problem with storage. A sampling bias exists by use of the GUM clinic setting. The authors suggest that only around 20% of (ex)PWIDs attending drug clinics are current injectors

Table S7: Prevalence of HCV among people in prison

**Reference**	**Country and study period**	**Study design**	**Sampling approach**	**Subgroup**	**Study population**	**Sample N**	**Prevalence anti-HCV (%)**	**Prevalence HCV-RNA (%)**	**Critical appraisal**	**General comments**
Alexiev 2016	Bulgaria 2010-2014	Cross-sectional study	Not reported	PLHIV in prison	HIV-1 positive prisoners nationwide, diagnosed between 2010-2014	59	82	60.1	- little detail in methods section<br>- selection study population not clear<br>- not clear whether study population is representative	
Jacomet 2015	France June 2010-Dec 2013	Prospective observational study	Exhaustive (screening)	People in prison	Prisoners of the Clermont-Ferrand and Riom prisons	357	4.7	1.5	- unclear whether study popualtion is representative	participation rate was a little over 50%
Treso 2011	Hungary June 2007-June 2009	Cross-sectional study	Exhaustive (screening)	People in prison who got a tattoo in prison	Prisoners who got a tattoo in prison in 20 Hungarian prisons	222	4.5		- No major comments	
Treso 2011	Hungary June 2007-June 2009	Cross-sectional study	Exhaustive (screening)	People in prison who have had unprotected sex	Prisoners who have had unprotected sex in 20 Hungarian prisons	927	4.2		- No major comments	
Treso 2011	Hungary June 2007-June 2009	Cross-sectional study	Exhaustive (screening)	People in prison with a tattoo	Prisoners with a tattoo 20 Hungarian prisons	993	4.6		- No major comments	
Treso 2011	Hungary June 2007-June 2009	Cross-sectional study	Exhaustive (screening)	PWID in prison	PWID Prisoners in 20 Hungarian prisons	209	22.5		- No major comments	
Sanarico 2016	Italy Jan-Dec 2013	Cross-sectional study	Not reported	PLHIV in prison	HIV+ Prisoners in detention centres of 7 Italian towns	69	78.3	65.2	- Data collection is not adequately described<br>- It is not clear whether the study population is representative of the source population	- Prisoners previously diagnosed with HIV with sample left over are included in the study, however the original sampling approach is not described
Monarca 2015	Italy July 2013	Cross-sectional study	Exhaustive (screening)	PLHIV in prison	HIV+ prisoners at 25 correctional institutions across Italy	338	55.9		- Methods serology not reported<br>- Limited information on selection of study population<br>- Representative of study population unclear<br>- Limited information on study population	The survey was conducted in almost a quarter of all Italian prisoners. The study was focused on HIV prevalence.

Babudieri 2005	Italy Nov 2011-Feb 2002	Cross-sectional study	Convenience sampling	People in prison who have had blood transfusions	Prisoners who have had blood transfusions from 8 prisons in different areas of Italy	76	48.7		- The study population may not be representative of all prisoners in Italy	
Babudieri 2005	Italy Nov 2011-Feb 2002	Cross-sectional study	Convenience sampling	People in prison who have had unprotected sex	Prisoners who have had unprotected sex from 8 prisons in different areas of Italy	227	43.2		- The study population may not be representative of all prisoners in Italy	
Babudieri 2005	Italy Nov 2011-Feb 2002	Cross-sectional study	Convenience sampling	People in prison with tattoos	Prisoners with tattoos from 8 prisons in different areas of Italy	463	51.2		- The study population may not be representative of all prisoners in Italy	
Babudieri 2005	Italy Nov 2011-Feb 2002	Cross-sectional study	Convenience sampling	PWID in prison	Prisoners who inject drugs in 8 prisons in different areas of Italy	296	74.7		- The study population may not be representative of all prisoners in Italy	
Dalgard 2009	Norway 2002	Cross-sectional study	Respondent-driven	PWID in prison	All users of a Neede syringe programme in Oslo for PWID with a history of incarceration	239	86		- The sample population may not be representative of the source population	
Grzeszczuk 2015	Poland 2008-2013	Cross-sectional study	Not reported	PLHIV in prison	Adult HIV-1 infected patients treated at a clinic in Bialystok, who have been imprisoned	107	93.5		- The methods are not clearly described<br>- Selection of study population not clear<br>- The study population is not representative of all HIV+ patients in Poland	
Vallejo 2015	Spain 2001-2006	Prospective observational study	Mixed	PWID in prison	Heroin injectors who have been imprisoned, recruited from the streets of Barcelona, Madrid and Seville, participating in the Itinere cohort	251	84.9		- Sample may not be representative of all recent heroin injectors	Small sample size and low follow-up rate. Participants may not be representative of all recent heroin injectors. 2/3 were HCV positive for HCV at baseline and around half of them were lost to folllow-up.
Cullen 2015	UK 2011	Cross-sectional study	Exhaustive (screening)	PWID in prison	Participants of UAM survey, recruiting at PWID services across England, Wales and Northern Ireland, who reported injecting during the preceding year and who have ever been to prison	1204	49.3		- unclear whether population is representative	

Table S8: Incidence of HCV among PLHIV

Reference	Country and study period	Study design	Sampling approach	Subgroup	Study population	Sample N	Incidence HCV	Critical appraisal	General comments
Apers 2013	Belgium 2001-2011	Retrospective observational study	Exhaustive (screening)	MSM living with HIV	HIV+ MSM attending the STI clinic of the Institute of Tropical Medicine, Antwerp	1105	1.36 cases per 100 person-years	- The sample population may not be representative of HIV+ MSM in Belgium	
Bottieau 2010	Belgium 2009	Retrospective observational study	Other	MSM living with HIV	HIV+ MSM attending the HIV/STI clinic of the Institute of Tropical Medicine, Antwerp	922	2.3-2.9 % per year	- Data collection may be inadequate<br>- The study population may not be representative of HIV+ MSM in Belgium	Data for 2001-2009 is presented in the article, but reported here for 2009 only. Periodic HCV screening was not systematically performed. HIV+ MSM attending the clinic were tested for HCV

									after each STI episode or after sexual contact with an HCV-infected partner. Those considered by the treating physician as being at high risk for HCV infection (for example, those with multiple sexual partners, GHB users and frequent visitors to known high-risk discotheques or saunas) were tested at least once a year for HCV.
Andersen 2014	Denmark Jan 2011-Jan 2013	Prospective observational study	Exhaustive (screening)	PLHIV	HIV+ patients actively followed at the outpatient clinic of Aarhus University Hospital	574	0.27 cases per 100 person-years	- Not clear how representative the study population is for all HIV patients in Denmark	
Barfod 2011	Denmark 2006-2009	Retrospective observational study	Not reported	MSM living with HIV	HIV infected MSM under treatment at a hospital department in Copenhagen	871	0.37 % per year	- Methods serology not reported<br>- Selection of study population not clear<br>- The denominator is incorrect	The denominator counts patients up until mid-2008, and since the nominator includes patients to the end of 2009, the incidence rate may be artificially high. The estimated yearly increase in patients fulfilling the denominator criteria is very unlikely to be more than 50, so the real incidence rate can be estimated to be no lower than 0.34% per year
Boesecke 2015	EU/EEA wide 2002-2013	Prospective observational study	Exhaustive (screening)	PLHIV	HIV patients from northern European centres participating in EuroSIDA	1578	4.4 12 year cumulative incidence (%)	- Methods serology not described	Only results for Northern Europe are shown here as this is the only region which contains solely EU/EEA countries (Denmark, Finland, Iceland, Ireland, Netherlands, Norway, Sweden, UK)
Ghosn 2006	France 1996-2005	Prospective observational study	Exhaustive (screening)	PLHIV	HIV patients from the multicentre prospective ANRS PRIMO cohort	402	0.43 cases per 100 person-years	- Original selection criteria study population not described<br>- unclear whether study population is representative	Less than 1% of this population of Primary HIV infection are injecting drug users; most of them became HIV+ through the sexual route.
Larsen 2011	France 2007	Surveillance study	Other	MSM living with HIV	HIV+ MSM attending HIV clinics throughout France participating in HEPAIG study	NR	0.36 % per year	- Methods serology not described<br>- The denominator is not reported	Incidence was based on the number of notified cases. Annual incidence was reported as cases per 10000 and computed in % per year here. Results for 2006 were also reported in the article. Study might have missed out the HIV-infected MSM patients who did not seek follow-up medical care
Jansen 2015	Germany 1996-2012	Prospective observational study	Not reported	MSM living with HIV	HIV-1 positive MSM from multiple centres nationwide	1838	1.54 cases per 100 person-years	- Selection of study population not clear	HCV incidence may be underestimated because of delayed antibody response to acute infection, but may be overestimated as positive samples were not confirmed with immunoblot
Puoti 2016	Italy 1997-2016	Prospective observational study	Not reported	PLHIV	Persons living with HIV enrolled in the ICONA study	4059	0.6 cases per 100 person-years	- Serological methods are not adequately described<br>- The methods are not clearly described<br>- Unclear if the study population is representative of the source population<br>- Theoretical background is limited<br>- The study population is not described	

Puoti<br>2016	Italy<br>1997-2016	Prospective observational study	Not reported	MSM living with HIV	MSM living with HIV enrolled in the ICONA study	NR	0.7 cases per 100 person-years	- Serological methods are not adequately described<br>- The methods are not clearly described<br>- Unclear if the study population is representative of the source population<br>- Theoretical background is limited<br>- The study population is not described	
Puoti<br>2016	Italy<br>1997-2016	Prospective observational study	Not reported	PWID living with HIV	PWID living with HIV enrolled in the ICONA study	NR	7.2 cases per 100 person-years	- Serological methods are not adequately described<br>- The methods are not clearly described<br>- Unclear if the study population is representative of the source population<br>- Theoretical background is limited<br>- The study population is not described	
Hullegie 2016	Netherlands Jan 2014-Jan 2015	Surveillance study	Exhaustive (screening)	MSM living with HIV	HIV+ MSM participating in the Dutch Acute HCV HIV study	8849	1.1 cases per 100 person-years	- little description relevance of the study<br>- study population not clearly described<br>- methods serology not described	The population included represents 86% of all HIV+ MSM in the Netherlands
van Rooijen 2016	Netherlands 2007-2011	Prospective observational study	Exhaustive (screening)	MSM living with HIV	All eligible HIV+ MSM who visited the STI clinic of Amsterdam	869	2.35 cases per 100 person-years	- It is unlikely that the study population is representative of the source population	Unable to detect acute infections which might count as incidence.
Sobrino-Vegas 2014	Spain<br>2004-2011	Prospective observational study	Not reported	PLHIV	HIV+ patients from the CoRIS cohort	2122	0.93 cases per 100 person-years	- The methods are not clearly described	Sampling methods of CoRIS were not reported. HCV testing was performed according to clinical criteria, rather than following a pre-specified screening scheme, so it is possible that those with available follow-up serologies are those in which a higher probability of HCV infection was suspected; thus rates of HCV diagnoses may be overestimated
Sobrino-Vegas 2014	Spain<br>2004-2011	Prospective observational study	Not reported	MSM living with HIV	HIV+ MSM from the CoRIS cohort	1421	0.75 cases per 100 person-years	- The methods are not clearly described	Sampling methods of CoRIS were not reported. HCV testing was performed according to clinical criteria, rather than following a pre-specified screening scheme, so it is possible that those with available follow-up serologies are those in which a higher probability of HCV infection was suspected; thus rates of HCV diagnoses may be overestimated
Martin 2016	UK<br>2004-2011	Prospective observational study	Exhaustive (screening)	MSM living with HIV	HIV+ MSM attending treatment clinics participating in UK CHIC	16533	1.07 cases per 100 person-years	-Methods serology not reported<br>-limited information on selection of study population ("Individuals were included in the analysis if they had ever attended one of the 11 centers since 2004")<br>-limited information on data collection<br>-study population is not described	Focus of the article is on the modelling section
Giraudon 2008	UK<br>Jan 2002-June 2006	Cross-sectional study	Other	MSM living with HIV	HIV+ MSM attending clinics in London and East Sussex	42985	0.9 cases per 100 person-years	- Methods serology not reported<br>- Study population not representative for all HIV+ MSM in the UK	Response bias may have led to an overestimation of incidence as non-responding clincs may be more likely to have fewer cases<br>Clinics were more likely to carry out routine<br>screening of all HIV+ MSM over time

Turner 2006	UK<br>1999-2005	Cross-sectional study	Exhaustive (screening)	MSM living with HIV	HIV positive MSM attending a HIV-outpatient clinic in London	308	0.9 cases per 100 person-years	- Methods serology not described<br>- Results are not clearly described<br>- It is not clear if the population in representative for the UK	

Table S9: Incidence of HCV among people in prison

Reference	Country and study period	Study design	Sampling approach	Subgroup	Study population	Sample N	Incidence HCV	Critical appraisal	General comments
Marco 2014	Spain<br>1992-2012	Retrospective observational study	Exhaustive (screening)	People in prison	Inmates of a prison in a province of Barcelona	2377	1.17 cases per 100 person-years	- No clear description of serological testing	Only 24.8 % (29/117) of the infected population stayed within the prison during the follow-up period, the rest had permission for release, so we can not determine if HCV occurred within or outside the prison.
Marco 2014	Spain<br>1992-2012	Retrospective observational study	Exhaustive (screening)	PWID in prison	Inmates of a prison in a province of Barcelona with a history of intravenous drug use	168	6.66 cases per 100 person-years	- No clear description of serological testing	Only 24,8 % (29/117) of the infected population stayed within the prison during the follow-up period, the rest had permission for release, so it can not be determined if HCV occurred within or outside the prison.
Taylor 2013	UK<br>June 2010-March 2011	Cross-sectional study	Exhaustive (screening)	People in prison	Prisoners in all closed prisons in Scotland	4904	1 cases per 100 person-years	- The methods may not be appropriate to accurately measure incidence	The incidence was estimated based on RNA positive/anti-HCV negative samples. New infections in people who had previously cleared the virus could not be identified. Prisoners incarcerated less than 75 days were excluded as the recent infection could be attributed to ouside factors.
Taylor 2013	UK<br>June 2010-March 2011	Cross-sectional study	Exhaustive (screening)	PWID in prison	Prisoners who inject drugs in all closed prisons in Scotland	479	2.0-2.9 %	- The methods may not be appropriate to accurately measure incidence	The incidence was estimated based on RNA positive/anti-HCV negative samples. New infections in people who had previously cleared the virus could not be identified. Prisoners incarcerated less then 75 days were excluded as the recent infection could be attributed to ouside factors.
